# C–H Trifluoromethylthiolation of aldehyde hydrazones

**DOI:** 10.3762/bjoc.20.242

**Published:** 2024-11-12

**Authors:** Victor Levet, Balu Ramesh, Congyang Wang, Tatiana Besset

**Affiliations:** 1 INSA Rouen Normandie, Univ Rouen Normandie, CNRS, Normandie Univ, COBRA UMR 6014, INC3M FR 3038, F-76000 Rouen, France; 2 Beijing National Laboratory for Molecular Sciences CAS key Laboratory of Molecular Recognition and Function CAS Research/Education Center for Excellence in Molecular Sciences Institute of Chemistry, Chinese Academy of Sciences, Beijing 100190, China

**Keywords:** C–H bond functionalization, C–S bond formation, hydrazones, synthetic method, trifluoromethylthiolation

## Abstract

The selective C–H trifluoromethylthiolation of aldehyde hydrazones afforded interesting fluorinated building blocks, which could be used as a synthetic platform. Starting from readily available (hetero)aromatic and aliphatic hydrazones, the formation of a C–SCF_3_ bond was achieved under oxidative and mild reaction conditions in the presence of the readily available AgSCF_3_ salt via a one-pot sequential process (28 examples, up to 91% yield). Mechanistic investigations revealed that AgSCF_3_ was the active species in the transformation.

## Introduction

Fluorinated molecules are of paramount importance [1–12] from industrial applications [13–15] to our daily lives thanks to the specific features [16] of the fluorine atom or the fluorinated groups. Aiming at pushing beyond the frontiers of knowledge in this very active research field, emergent fluorinated groups [17–20] such as the SCF_3_ moiety [21–51], an interesting fluorinated moiety with unique electron-withdrawing character and lipophilicity [52–53], have recently garnered interest from the scientific community. Various reagents and chemical transformations have been elaborated in this context over the years [21–51]. Despite these recent advances, the design of highly functionalized trifluoromethylthiolated molecules, which could be used as synthetic handles for synthesizing more complex molecules, is still appealing. In this context, we turned our attention to the trifluoromethylthiolated hydrazones, an interesting building block. Indeed, aldehyde hydrazones have been well studied and used in various transformations [54–64]. In consequence, a large number of transition-metal-catalyzed or radical-mediated processes for C–H functionalization of aldehyde hydrazones has flourished over the years.

**Scheme 1:**
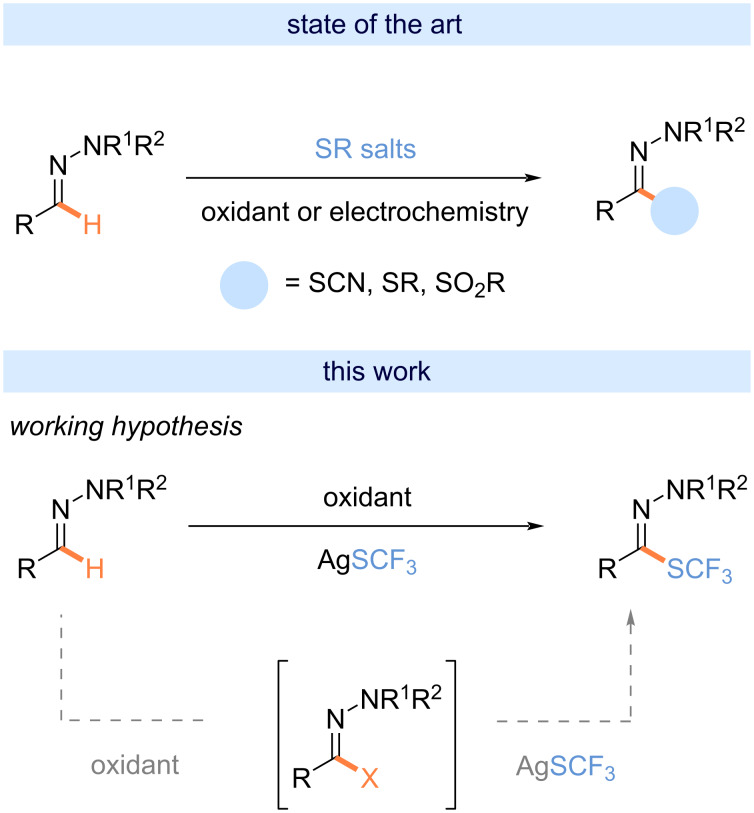
State of the art and this work.

An ideal scenario for a direct and sustainable synthetic route towards trifluoromethylthiolated hydrazones will be the direct C–H functionalization of the corresponding aldehyde hydrazone, an uncharted transformation to date. Forging a C–S bond by the direct C–H-bond functionalization of hydrazones is still underdeveloped. Except for transformations leading to the corresponding sulfur-containing heteroarenes, only a few methods have been developed (Scheme 1). In 1988, Lee and co-workers reported the synthesis of SR-containing hydrazones in a two-step process (chlorination [65] then reaction with thiols) from readily available aldehyde-derived hydrazones [66]. Wang et al. developed a method to access thiocyanated derivatives including an aldehyde hydrazone (a unique example) in 70% yield thanks to the in situ generation of SCN-succinimide from NCS and NH_4_SCN (Scheme 1) [67]. In the same vein, the group of Monteiro [68], then Hajra [69], independently, reported the synthesis of 5-thioxo-1,2,4-triazolium inner salts by the nucleophilic thiocyanation of *N,N*-dialkylhydrazonoyl bromides, in situ generated from aldehyde-derived hydrazones in the presence of an oxidant (NBS, (NH_4_)_2_S_2_O_8_), Scheme 1). In 2024, the synthesis of 2‑imino-1,3,4-thiadiazoles was achieved by cyclization of aryl hydrazones with aryl isothiocyanates promoted by elemental sulfur [70]. In the course of their studies for the thiocyanation of ketene dithioacetals, Yang, Wang and co-workers developed an electrochemical oxidization-based synthetic strategy to circumvent the need for external oxidants. In this context, a unique example of the thiocyanation of a hydrazone was depicted [71]. A key feature of the approach is to circumvent the need for external oxidants. In the same vein, the group of Hajra [72] and Yang [73], independently, investigated the electrochemical C–H sulfonylation of a library of aldehyde hydrazones using sodium sulfinates.

These seminal works brought interesting proofs of concept for the synthesis of SR-containing hydrazones. Inspired by these previous works and taking benefit from our in-home expertise to forge N–SCF_3_ bond (after chlorination/anion metathesis with AgSCF_3_ from the corresponding R^1^R^2^NH) [74], we assumed that a one-pot two-step process could be an efficient strategy for the trifluoromethylthiolation of hydrazones. Herein, the synthesis of trifluoromethylthiolated hydrazones from aldehyde hydrazones is depicted.

## Results and Discussion

At the outset of the study, the morpholine hydrazone derived from 4-nitrobenzaldehyde was selected as a model substrate (Table 1). The latter was engaged in a two-step process: 1) halogenation to provide the corresponding *N,N*-hydrazonoyl bromide, which will then undergo an anion metathesis upon the addition of AgSCF_3_ to the reaction mixture. When the reaction was conducted in the presence of NBS in acetonitrile for 10 min, followed by the addition of AgSCF_3_, the desired product was isolated in 91% yield. A total selectivity for the formation of the *Z* isomer was observed as ascertained by 2D NMR (for more details, see Supporting Information File 1) [75]. Different reagents for the bromination or chlorination were also evaluated (Table 1, entries 1–3) and NBS was the most efficient one (Table 1, entry 1).

**Table 1:** Optimization of the reaction conditions.^a^

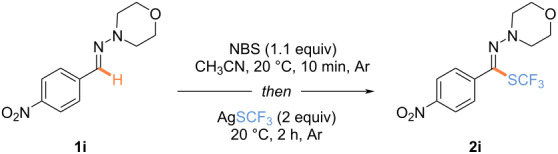		

Entry	Deviation from reaction conditions	Yield (%)^b^

1	none	91
2	*N*-bromophthalimide instead of NBS	86^c^
3	NCS instead of NBS	ND

^a^Reaction conditions: hydrazone **1i** (0.15 mmol, 1.0 equiv), oxidant (0.165 mmol, 1.1 equiv), in CH_3_CN (0.4 M), 20 °C, 10 min, then AgSCF_3_ (0.3 mmol, 2.0 equiv), under argon. ^b^Isolated yields are given. ^c^The product was isolated with an inseparable impurity. ND = not determined.

**Scheme 2:**
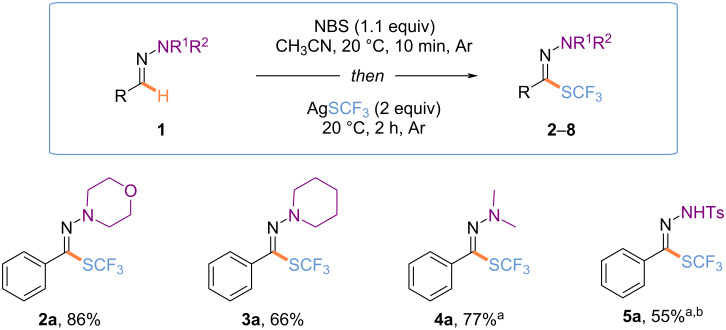
Reaction conditions: hydrazone (0.3 mmol, 1.0 equiv), NBS (0.33 mmol, 1.1 equiv), in CH_3_CN (0.4 M), 20 °C, 10 min; then, AgSCF_3_ (0.6 mmol, 2.0 equiv), under argon. Isolated yields are given. ^a^Products **4a** and **5a** were isolated with an inseparable impurity. ^b^Reaction performed at 0 °C for the 1st step, and 20 °C for the 2nd one.

With the best reaction conditions in hand, the nature of the hydrazone part was first investigated (Scheme 2). Under standard reaction conditions, electron-enriched hydrazones provided the expected products in high yields (**2a**, **3a**, **4a**). Note, that in the case of the *N*-tosylhydrazone, further optimization reactions were required (for more details, see Supporting Information File 1), and reducing the temperature for the halogenation reaction was beneficial to the outcome of the reaction, affording **5a** in 55% yield. However, some other hydrazones **6a**–**8a** were reluctant (for more details, see Supporting Information File 1) [75].

**Scheme 3:**
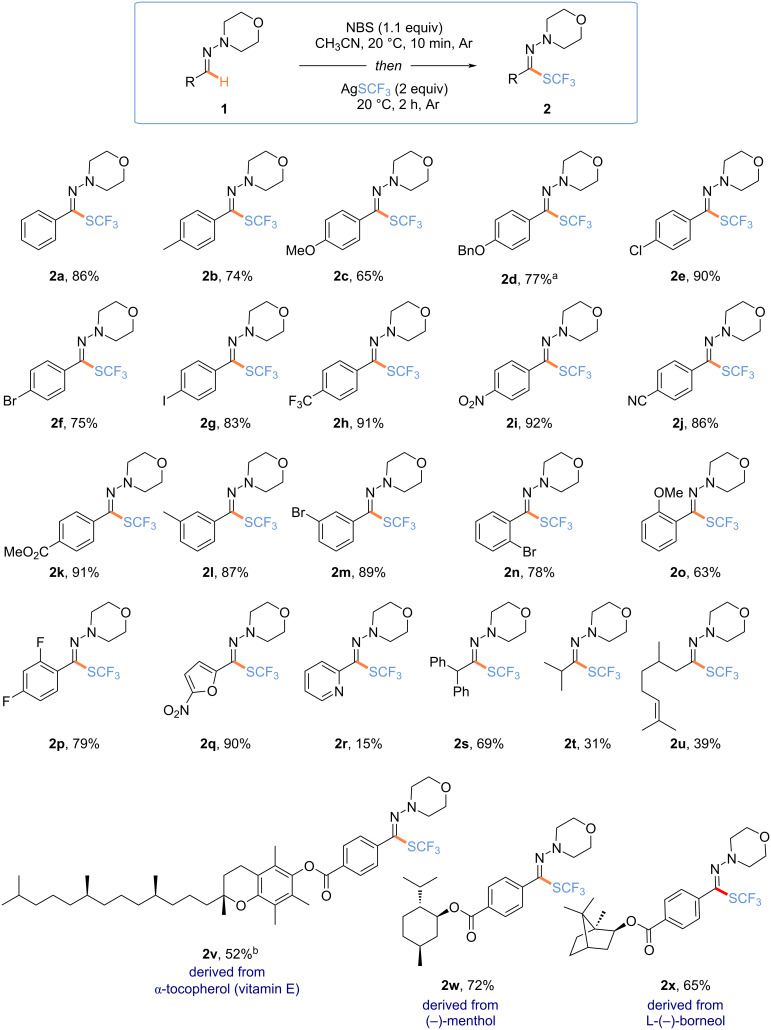
Scope of the reaction. Reaction conditions: **1** (0.3 mmol, 1.0 equiv), NBS (0.33 mmol, 1.1 equiv) in CH_3_CN (0.4 M), 20 °C, 10 min; then, AgSCF_3_ (0.6 mmol, 2.0 equiv), 2 h, under argon. ^a^0.15 mmol reaction scale. ^b^Product **2v** was isolated with an inseparable impurity.

Then, the scope of the reaction was investigated using the hydrazones derived from morpholine (Scheme 3). Hydrazones derived from aromatic aldehydes (**1a**–**p**) were first investigated. It turned out that *para*-substituted compounds with electron-rich groups (e.g., OMe, OBn), halogens (Cl, Br, I), and electron-withdrawing groups (e.g., CF_3_) were smoothly trifluoromethylthiolated. In the same vein, *meta-* and *ortho*-substituted derivatives (**1l**–**o**) were converted into the corresponding fluorinated analogs. The functionalization of the 2,4-difluorophenyl derivative (**1p**) and the heteroaromatic compounds such as furan (**1q**) as well as pyridine (**1r**) derivatives went smoothly, with the lower yield obtained in the case of **2r** being explained by a tedious purification. Interestingly, the methodology was successfully applied to the functionalization of aliphatic hydrazones **1s** and **1t** and even the hydrazone derived from citronellal **1u**. The method was functional group-tolerant to various functional groups (nitro, CN, ester, alkenes) and halogens allowing an array of post-functionalization reactions. Finally, the trifluoromethylthiolation of molecules derived from compounds of interest was achieved to illustrate the synthetic utility of the method. Hence, the desired products **2v**–**x** were efficiently isolated.

**Scheme 4:**
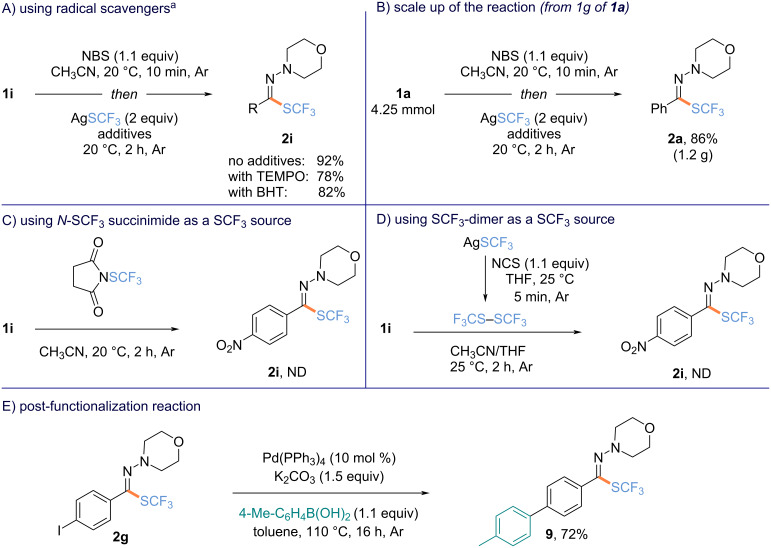
Mechanistic investigations and post-functionalization reactions. ^a19^F NMR yields using α,α,α-trifluoroacetophenone as an internal standard. ND = not detected.

To get more insights into the transformation, additional experiments were conducted. First, the reaction was repeated in the presence of radical scavengers, namely 2,2,6,6-tetramethylpiperidin-1-yl)oxyl (TEMPO) or di-*tert*-butylhydroxytoluene (BHT), and no significant impact on the outcome of the reaction was noticed (Scheme 4A). Pleasingly, the scale up of the reaction was smoothly conducted. Under standard reaction conditions, product **2a** (1.2 g) was afforded starting from **1a** (1 g), showcasing the robustness of the transformation (Scheme 4B). Intrigued about the nature of the active source of SCF_3_ in the transformation, experiments with different SCF_3_ sources were conducted. First, we hypothesized that trifluoromethylthiolated succinimide, which might be in situ generated from NBS and AgSCF_3_, could be the active species. When the reaction was carried out in the presence of this electrophilic source and **1a**, no expected product was detected (Scheme 4C). Having in mind that in the presence of an oxidant, the SCF_3_ dimer (SCF_3_)_2_ might be generated, an additional test was realized. In the presence of NCS in THF, AgSCF_3_ was converted into the corresponding dimer in 5 min (monitored by ^19^F NMR). Then, the reaction was conducted in the presence of **1a** in a THF/MeCN mixture (1:1) [75], but no product was detected (Scheme 4D). Based on these experiments and literature data [66], a two-step one-pot process was suggested based on 1) the bromination of the hydrazone **1** followed by 2) the anion metathesis in the presence of AgSCF_3_. Finally, to further illustrate the synthetic utility of the trifluoromethylthiolated hydrazones, product **2g** was further functionalized. In the presence of 4-methylboronic acid, the arylation of **2g** occurred and the expected product was isolated in 72% yield with the SCF_3_-hydrazone motif remaining untouched (Scheme 4E) [42].

## Conclusion

In summary, a one-pot two-step process has been developed for the trifluoromethylthiolation of aldehyde hydrazones. A myriad of (hetero)aromatic and aliphatic hydrazones were efficiently functionalized including analogs of compounds of interest (28 examples, up to 91% yield) using readily available reagents, namely NBS and the nucleophilic reagent AgSCF_3_. This approach provides a straightforward access to an unprecedented class of trifluoromethylthiolated derivatives. This method offers new avenues for synthesizing a plethora of valuable SCF_3_-containing molecules using the synthetic potential of hydrazones in organic synthesis.

## Experimental

**General procedure for the preparation of trifluoromethylthiolated products 2–6:** An oven-dried 10 mL reaction tube equipped with a stirring bar was charged with the hydrazone derivative (0.3 mmol, 1.0 equiv) and CH_3_CN (0.7 mL). The mixture was stirred until the solubilization of the reagent. Then, recrystallized NBS (58.7 mg, 0.33 mmol, 1.1 equiv) was added, and the reaction mixture was stirred for 5–10 minutes, after which, AgSCF_3_ (125.0 mg, 0.6 mmol, 2.0 equiv) was added. The reaction was stirred for another 2 hours at room temperature. α,α,α-Trifluoroacetophenone (42 μL, 0.3 mmol, 1.0 equiv) was added as an internal standard for determining the ^19^F NMR yield. The mixture was then filtered on a pad of celite and rinsed with CH_2_Cl_2_. The solution was then washed with brine twice (20 mL) and the organic layers were collected separately, dried over MgSO_4_, and concentrated in vacuo. The crude was purified by column chromatography on silica gel, flash chromatography to afford the desired product **2**–**6**.

## Supporting Information

File 1 Full experimental procedures, characterization of products, details of mechanistic studies, and spectral data.

## Data Availability

All data that supports the findings of this study is available in the published article and/or the supporting information of this article.
