# Supplementary material for: C–H Trifluoromethylthiolation of aldehyde hydrazones
Source: Beilstein J Org Chem. 2024 Nov 12;20:2883–90. doi: 10.3762/bjoc.20.242 (PMC11571951; doi:10.3762/bjoc.20.242)
Supplement: File 1 — Full experimental procedures, characterization of products, details of mechanistic studies, and spectral data. [file Beilstein_J_Org_Chem-20-2883-s001.pdf]

BEILSTEIN JOURNAL OF ORGANIC CHEMISTRY

Supporting Information

for

C–H Trifluoromethylthiolation of aldehyde hydrazones

Victor Levet, Balu Ramesh, Congyang Wang and Tatiana Besset

Beilstein J. Org. Chem. **2024,** *20,* 2883–2890. doi:10.3762/bjoc.20.242

Full experimental procedures, characterization of products, details of mechanistic studies, and spectral data

License and Terms: This is a supporting information file under the terms of the Creative Commons Attribution License (https://creativecommons.org/licenses/by/4.0). Please note that the reuse, redistribution and reproduction in particular requires that the author(s) and source are credited and that individual graphics may be subject to special legal provisions.
The license is subject to the *Beilstein Journal of Organic Chemistry* terms and conditions: (https://www.beilstein-journals.org/bjoc/terms)

Table of contents

1. General information S2
2. Materials S2
3. Screening of oxidant S3
4. General procedures for the synthesis of the aldehyde hydrazone derivatives S3
5. General procedure for the preparation of trifluoromethylthiolated products S5
6. Purification and characterization of the aldehyde hydrazone derivatives S6
7. Purification and characterization of the trifluoromethylthiolated products S8
8. Reluctant substrates S17
9. Mechanistic studies S18
   - Study on the role of the $(SCF_3)_2$ dimer S18
   - Test with SCF_3 succinimide as a SCF_3 source S19
   - Experiments with radical scavengers: S19
10. Post-functionalization reaction S20
11. NMR Spectra S21
12. References S68

1. General information

All reactions were carried out using oven-dried glassware and magnetic stirring under argon unless otherwise stated. Reaction temperatures are reported as the temperature of the bath surrounding the vessel. Analytical thin-layer chromatography was performed on silica gel aluminum plates with F-254 indicator and visualized by UV light (254 nm) and/or chemical staining with a $KMnO_4$ solution or vanillin solution. Flash column chromatography was performed using 0.040–0.063 nm silica. Flash column chromatography was performed using 0.040–0.063 nm silica gel. ^1H NMR spectra were recorded on a Bruker Ascend Evo 400 spectrometer at 400.2 MHz, ^{13}C {^1H} NMR spectra at 100.6 MHz, ^{19}F {^1H} NMR spectra at 376.6 MHz Chemical shifts (δ) are quoted in ppm relative to TMS (^1H) and $CFCl_3$ (^{19}F). Coupling constants (*J*) are quoted in Hz. The following abbreviations were used to show the multiplicities: s: singlet, d: doublet, t: triplet, q: quadruplet, dd: doublet of doublet, td: triplet of doublet, m: multiplet, qd: quartet of doublet, hept: heptet. The residual solvent signals were used as references ($CDCl_3$: δ H = 7.26 ppm, δ C = 77.00 ppm or relative to external $CFCl_3$: δ F = 0 ppm). High-resolution mass spectrometry (HRMS) was carried out on an electronic (EI^+) or electrospray ionization (ESI^+) source and with a micro-TOF analyzer. Infrared spectra were recorded on a PerkinElmer Spectrum 100, the wave numbers (ν) of recorded IR-signals (ATR) are quoted in cm^{-1}. Melting points were measured on a STUART SMP3 melting point apparatus in open capillaries. Melting points were recorded on a Stuart Scientific Analogue SMP3.

2. Materials

Anhydrous acetonitrile (CH_3CN), methanol (CH_3OH) and dichloroethane (DCE) were purchased from Acros Organics (Solvents Extra Dry over Molecular Sieves, AcroSeal®). Dichloromethane (CH_2Cl_2) was distilled over CaH_2 and tetrahydrofuran was distilled over sodium and benzophenone prior to use. *N*-Aminomorpholine was purchased from Sigma Aldrich. $AgSCF_3$ was synthesized following the literature procedure.[1] *N*-Bromosuccinimide was recrystallized from 95 °C water, according to the literature.[2] Known hydrazones (*E*)-1-phenyl-*N*-(piperidin-1-yl)methanimine, (*E*)-2-benzylidene-1,1-dimethylhydrazine, and (*E*)-benzylidenehydrazine were obtained from the commercially available benzaldehyde following the literature procedure.[3]

3. Screening of oxidant

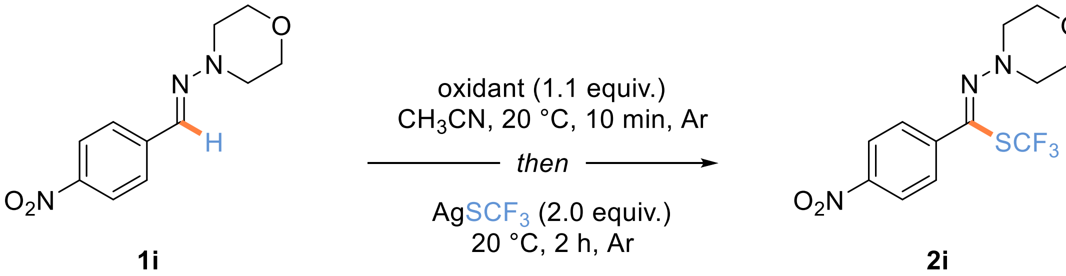

Entry	Oxidant	^{19}F NMR yield (%)
1	NBS	99 (91)
2	NCS	ND
3	*N*-Bromophthalimide	95 (86)[2]
5	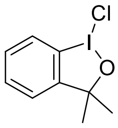	ND
6	SO_2Cl_2	traces

Reaction conditions: hydrazone **1i** (0.15 mmol, 1.0 equiv), oxidant (0.165 mmol, 1.1 equiv), in CH_3CN (0.4 M), 20 °C, 10 min, then $AgSCF_3$ (0.6 mmol, 2.0 equiv), under Ar, 2 h. ^{19}F NMR yields were determined using α,α,α-trifluoroacetophenone as an internal standard. Isolated yields were reported in parentheses. [2]The product was isolated in the presence of an inseparable impurity. ND = Not determined.

4. General procedures for the synthesis of the aldehyde hydrazone derivatives

General procedure A

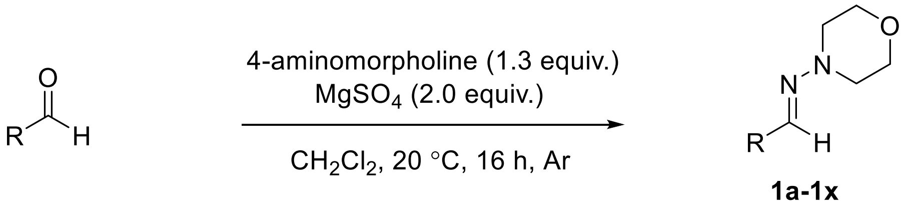

The aminomorpholine hydrazone derivatives **1a–u** were obtained from the commercially available corresponding aldehyde derivatives following the literature procedures:[4,5] An oven-dried 25 mL flask, equipped with a stirring bar was charged with the aldehyde (2 mmol, 1.0 equiv), $MgSO_4$ (480 mg, 4 mmol, 2.0 equiv) and CH_2Cl_2 (3 mL) in an Ar atmosphere. The reaction mixture was stirred at 20 °C and the morpholine hydrazone was added slowly (250 µL, 2.6 mmol, 1.3 equiv). The reaction was stirred at 20 °C overnight under Ar. The crude mixture

was filtered over celite pad and purified by silica gel flash column chromatography to afford the desired product.

The aldehydes such as (*R*)-2,5,7,8-tetramethyl-2-((4*R*,8*R*)-4,8,12-trimethyltridecyl)chroman-6-yl 4-formylbenzoate, (2*R*,5*S*)-2-isopropyl-5-methylcyclohexyl 4-formylbenzoate, and (1*S*,2*S*,4*S*)-1,7,7-trimethylbicyclo[2.2.1]heptan-2-yl 4-formylbenzoate were synthesized following the literature procedures.[5] Later, the synthesized aldehydes were utilized to prepare the known aminomorpholine hydrazone **1v**,[5] and unknown aminomorpholine hydrazones **1w** and **1x** from the following literature procedures.[4,5]

General procedure B

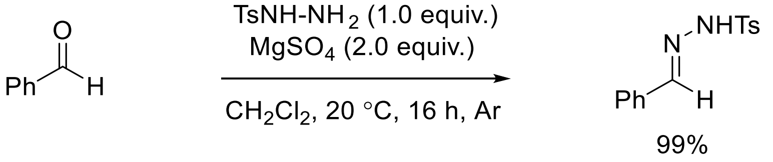

This procedure was adapted from Wang et al:[6] in an oven-dried 25 mL flask, equipped with a stirring bar, benzaldehyde (1.02 mL, 10 mmol, 1.0 equiv) was added slowly to a solution of tosylhydrazone (1.86 g, 10 mmol, 1.0 equiv) in MeOH (5 mL). The reaction mixture was stirred at 20 °C for 16 h under Ar. The precipitate was filtered over celite pad and washed abundantly with petroleum ether (3 × 15 mL), to yield the desired product (*E*)-*N'*-benzylidene-4-methylbenzenesulfonohydrazide (2.71 g, 99%) as a white powder.

General procedure C

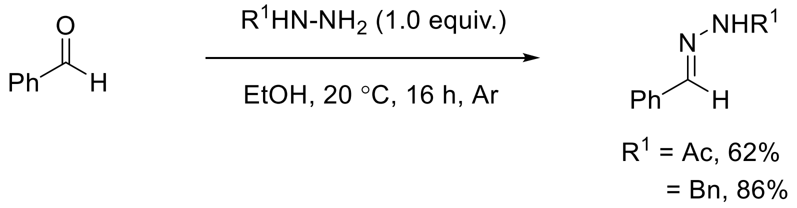

This procedure was adapted from Leighton et al:[7] in an oven-dried 25 mL flask, equipped with a stirring bar, the aldehyde (2 mmol, 1.0 equiv) was added to a solution of the acyl hydrazide (2 mmol, 1.0 equiv) in EtOH (4 mL). The reaction mixture was stirred at 20 °C for 16 h under Ar. The crude mixture was filtered over celite pad and purified by silica gel flash column chromatography with 9:1 mixture of acetone/CH_2Cl_2, to yield the desired product (*E*)-*N'*-benzylideneacetohydrazide (0.198 g, 62%) as a white powder or (*E*)-1-benzyl-2-benzylidenehydrazine (0.38 g, 86%) as a white powder.

5. General procedure for the preparation of trifluoromethylthiolated products

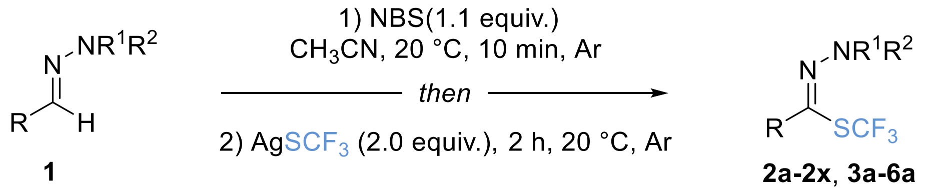

An oven-dried 10 mL reaction tube equipped with a stirring bar was charged with the hydrazone derivative **1** (0.3 mmol, 1.0 equiv) and CH_3CN (0.7 mL). The mixture was stirred until the solubilization of the reagent. Then, recrystallized NBS (58.7 mg, 0.33 mmol, 1.1 equiv) was added, and the reaction mixture was stirred for 5–10 minutes. After which, $AgSCF_3$ (125.0 mg, 0.6 mmol, 2.0 equiv) was added. The reaction was stirred for another 2 hours at 20 °C under Ar. α,α,α-trifluoroacetophenone (42 μL, 0.3 mmol, 1.0 equiv) was added as an internal standard for determining the ^{19}F NMR yield. The mixture was then filtered over celite pad and rinsed with CH_2Cl_2 (25 mL). The organic layer was then washed with brine (2 × 20 mL), dried over $MgSO_4$, and concentrated in a vacuum. The crude was purified by flash column chromatography on silica gel to afford the desired product.

6. Purification and characterization of the aldehyde hydrazone derivatives

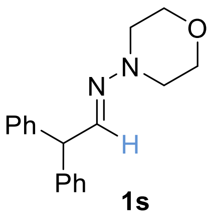

1s

(*E*)-*N*-Morpholino-2,2-diphenylethan-1-imine (1s): The starting material was synthesized using the general procedure A. The product was purified by silica gel flash column chromatography eluting with petroleum ether/ethyl acetate (9:1). The desired product was isolated as a colorless oil (404 mg, 72%). R_f (petroleum ether/ethyl acetate = 9:1): 0.52. **^{1}H NMR** (400.2 MHz, $CDCl_3$) δ 7.26 – 7.12 (m, 11H), 4.86 (d, J = 7.4 Hz, 1H), 3.78 – 3.73 (m, 4H), 2.98 – 2.93 (m, 4H). **^{13}C NMR** (100.6 MHz, $CDCl_3$) δ 141.9, 141.4, 128.6, 128.5, 126.6, 66.4, 54.1, 52.2. **IR** (neat, cm^{-1}) ν: 2851, 1580, 1447, 1276, 1112, 990, 698, 596, 396. **HRMS** (EI^+) calcd for $C_{18}H_{20}F_3N_2O$ m/z 280.1576 $[M]^+$, Found 280.1568 (Δ = –2.65 ppm).

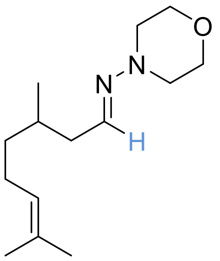

1u

(*E*)-3,7-Dimethyl-*N*-morpholinooct-6-en-1-imine (1u): The starting material was synthesized using the general procedure A. The product was purified by silica gel flash column chromatography eluting with petroleum ether/ethyl acetate (95:5). The desired product was isolated as a colorless oil (448 mg, 94%). R_f (petroleum ether/ethyl acetate = 95:5): 0.56. **^{1}H NMR** (400.2 MHz, $CDCl_3$) δ 6.97 (t, J = 5.8 Hz, 1H), 5.15 – 5.02 (m, 1H), 3.85 – 3.78 (m, 4H), 2.97 – 2.90 (m, 4H), 2.30 – 2.21 (m, 1H), 2.14 – 2.04 (m, 1H), 2.03 – 1.91 (m, 2H), 1.72 – 1.63 (m, 4H), 1.59 (s 3H), 1.43 – 1.32 (m, 1H), 1.27 – 1.14 (m, 1H), 0.92 (d, J = 6.7 Hz, 3H). **^{13}C NMR** (100.6 MHz, $CDCl_3$) δ 141.7, 131.3, 124.5, 66.4, 52.6, 40.1, 36.8, 31.4, 25.7, 25.4, 19.4, 17.6. **IR** (neat, cm^{-1}) ν: 2956, 2851, 1453, 1376, 1272, 1117, 1091, 988, 863, 734, 664, 393. **HRMS** (EI^+) calcd for $C_{14}H_{26}N_2O$ m/z 238.2045 $[M]^+$, Found 238.2036 (Δ = –3.74 ppm).

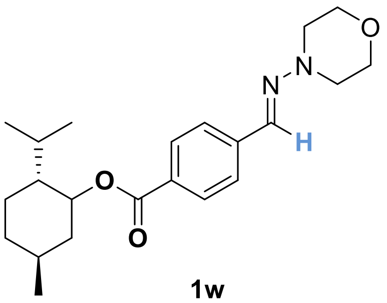

1w

(2*R*,5*S*)-2-Isopropyl-5-methylcyclohexyl 4-((*E*)-(morpholinoimino)methyl)benzoate (1w): The starting material was synthesized using the general procedure A. The product was purified by silica gel flash column chromatography (height 13 cm, width 3.5 cm) eluting with petroleum ether/ethyl acetate (95:5). The desired product was isolated as a white solid (650 mg, 87%).

mp: 91 – 93 °C. R_f (petroleum ether/ethyl acetate = 95:5): 0.38. **^{1}H NMR** (400.2 MHz, $CDCl_3$) δ 8.04 –7.98 (m, 2H), 7.66 – 7.61 (m, 2H), 7.56 (s, 1H), 4.92 (td, J = 12.0, 4.4 Hz, 1H), 3.91 – 3.85 (m, 4H), 3.25 – 3.19 (m, 4H), 2.16 – 2.09 (m, 1H), 2.00 – 1.91 (m, 1H), 1.76 – 1.68 (m, 2H), 1.60 – 1.49 (m, 2H), 1.19 – 1.04 (m, 2H), 0.98 – 0.86 (m, 7H), 0.79 (d, J = 8 Hz, 3H). **^{13}C NMR** (100.6 MHz, $CDCl_3$) δ 165.9, 140.1, 134.1, 130.1, 129.8, 125.8, 74.8, 66.3, 51.5, 47.2, 41.0, 34.3, 31.4, 26.5, 23.7, 22.0, 20.7, 16.5. **IR** (neat, cm^{-1}) ν: 2923, 1707, 1453, 1265, 1117, 1004, 902, 769, 699, 512, 399. **HRMS** (ESI^+) calcd for $C_{22}H_{33}N_2O_3$ *m/z* 373.2485 $[M+H]^+$, Found 373.2487 (Δ = 0.34 ppm).

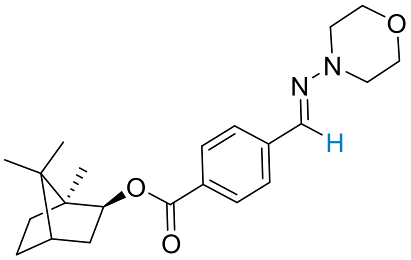

1x

(1*S*,2*S*,4*S*)-1,7,7-Trimethylbicyclo[2.2.1]heptan-2-yl 4-((*E*)-(morpholinoimino)methyl)benzoate (1x): The starting material was synthesized using the general procedure A. The product was purified by silica gel flash column chromatography (height 13 cm, width 3.5 cm) eluting with petroleum ether/ethyl acetate (95:5). The desired product was isolated as a colorless oil (620 mg, 84%). R_f (petroleum ether/ethyl acetate = 95:5): 0.38. **^{1}H NMR** (400.2 MHz, $CDCl_3$) δ 8.05 – 7.99 (m, 2H), 7.67 – 7.62 (m, 2H), 7.57 (s, 1H), 5.13 – 5.08 (m, 1H), 3.92 – 3.86 (m, 4H), 3.25 – 3.20 (m, 4H), 2.52 – 2.42 (m, 1H), 2.18 – 2.09 (m, 1H), 1.86 – 1.75 (m, 1H), 1.74 (m, 1H), 1.46 – 1.36 (m, 1H), 1.35 – 1.27 (m, 1H), 1.12 (dd, J = 12, 4 Hz, 1H), 0.97 (s, 3H), 0.91 (s, 6H). **^{13}C NMR** (100.6 MHz, $CDCl_3$) δ 166.6, 140.2, 134.0, 130.1, 129.8, 125.8, 80.5, 66.4, 51.5, 49.1, 47.9, 45.0, 36.9, 28.1, 27.4, 19.7, 18.9, 13.6. **IR** (neat, cm^{-1}) ν: 2922, 1700, 1588, 1450, 1360, 1271, 1172, 1114, 1004, 769, 624, 519, 407. **HRMS** (ESI^+) calcd for $C_{22}H_{31}N_2O_3$ *m/z* 371.2329 $[M+H]^+$, Found 371.2332 (Δ = 0.81 ppm).

7. Purification and characterization of the trifluoromethylthiolated products

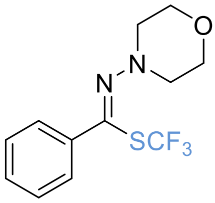

2a

Trifluoromethyl (*Z*)-*N*-morpholinobenzimidothioate (2a): The product was purified by silica gel flash column chromatography eluting with petroleum ether/ethyl acetate (9:1). The desired product was isolated as a clear solid (74.9 mg, 86%). mp: 59 – 61 °C. R_f (petroleum ether/ethyl acetate = 9:1): 0.46. **^{1}H NMR** (400.2 MHz, $CDCl_3$) δ 7.55 – 7.35 (m, 5H), 3.94 – 3.84 (m, 4H), 3.02 – 2.92 (m, 4H). **^{19}F NMR** (376.6 MHz, $CDCl_3$) δ –36.9 (s, 3F). **^{13}C NMR** (100.6 MHz, $CDCl_3$) δ 159.2, 134.1, 130.3, 129.0 (q, J = 310.8), 128.5, 128.1, 65.9, 54.2. **IR** (neat, cm^{-1}) ν: 2971, 2859, 1698, 1572, 1443, 1263, 1139, 1103, 973, 862, 861, 697, 451. **HRMS** (EI^+) calcd for $C_{12}H_{13}F_3N_2OS$ *m/z* 290.0701 $[M]^+$, Found 290.0701 (Δ = 0.1 ppm).

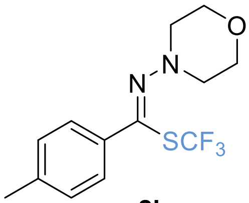

2b

Trifluoromethyl (*Z*)-4-methyl-*N*-morpholinobenzimidothioate (2b): The product was purified by silica gel flash column chromatography eluting with petroleum ether/ethyl acetate (9:1). The desired product was isolated as a light orange oil (67.7 mg, 74%). R_f (petroleum ether/ethyl acetate = 9:1): 0.58. **^{1}H NMR** (400.2 MHz, $CDCl_3$) δ 7.43 – 7.36 (m, 2H), 7.24 – 7.17 (m, 2H), 3.90 – 3.82 (m, 4H), 2.99 – 2.91 (m, 4H), 2.38 (s, 3H). **^{19}F NMR** (376.6 MHz, $CDCl_3$) δ –36.9 (s, 3F). **^{13}C NMR** (100.6 MHz, $CDCl_3$) δ 159.4, 140.6, 131.3, 128.9 (q, J = 310.5 Hz), 128.7, 128.4, 65.9, 54.2, 21.4. **IR** (neat, cm^{-1}) ν: 2855, 1583, 1456, 1431, 1262, 1140, 1099, 978, 863, 755, 698, 629. **HRMS** (EI^+) calcd for $C_{13}H_{15}F_3N_2OS$ *m/z* 304.0857 $[M]^+$, Found 304.0865 (Δ = 2.69 ppm).

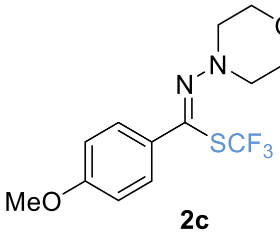

2c

Trifluoromethyl (*Z*)-4-methoxy-*N*-morpholinobenzimidothioate (2c): The product was purified by silica gel flash column chromatography eluting with petroleum ether/ethyl acetate (8:2). The desired product was isolated as an amorphous orange solid (62.3 mg, 65%). R_f (petroleum ether/ethyl acetate = 8:2): 0.39. **^{1}H NMR** (400.2 MHz, $CDCl_3$) δ 7.49 – 7.43 (m, 2H), 6.94 – 6.89 (m, 2H), 3.90 – 3.80 (m, 4H), 3.83 (s, 3H), 2.96 – 2.92 (m, 4H). **^{19}F NMR** (376.6 MHz, $CDCl_3$) δ –36.9 (s, 3F). **^{13}C NMR** (100.6 MHz, $CDCl_3$) δ 161.3, 158.9, 130.7, 129.2 (q, J = 310.5), 126.6, 113.5, 65.9, 55.3, 54.3. **IR** (neat, cm^{-1}) ν: 2963, 2841, 1607, 1509, 1246, 1133, 1100, 975, 834, 754, 642, 501. **HRMS** (EI^+) calcd for $C_{13}H_{15}F_3N_2O_2S$ *m/z* 320.0806 $[M]^+$, Found 320.0817 (Δ = 3.42 ppm).

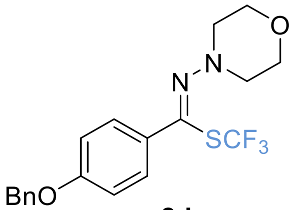

2d

Trifluoromethyl (*Z*)-4-(benzyloxy)-*N*-morpholinobenzimidothioate (2d): This compound was synthesized on 0.15 mmol scale. The product was purified by silica gel flash column chromatography eluting with petroleum ether/ethyl acetate (8:2). The desired product was isolated as a white solid (45.7 mg, 77%). mp: 107 – 109 °C. R_f (petroleum ether/ethyl acetate = 8:2): 0.54. **^{1}H NMR** (400.2 MHz, $CDCl_3$) δ 7.51 – 7.31 (m, 7H), 7.03 – 6.96 (m, 2H), 5.10 (s, 2H), 3.93 – 3.81 (m, 4H), 2.99 – 2.93 (m, 4H). **^{19}F NMR** (376.6 MHz, $CDCl_3$) δ –36.8 (s, 3F). **^{13}C NMR** (100.6 MHz, $CDCl_3$) δ 160.4, 158.7, 136.4, 130.1, 128.7 (q, J = 310.8 Hz), 128.6, 128.1, 127.4, 126.9, 114.5, 70.0, 65.9, 54.3. **IR** (neat, cm^{-1}) ν: 2961, 2854, 1582, 1456, 1431, 1260, 1199, 977, 862, 754, 698, 630. **HRMS** (EI^+) calcd for $C_{19}H_{19}F_3N_2O_2S$ *m/z* 396.1119 $[M]^+$, Found 396.1124 (Δ = 1.15 ppm).

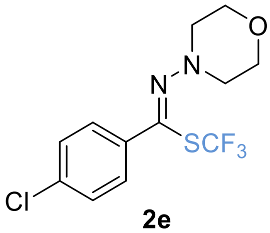

2e

Trifluoromethyl (*Z*)-4-chloro-*N*-morpholinobenzimidothioate (2e): The product was purified by silica gel flash column chromatography eluting with petroleum ether/ethyl acetate (9:1). The desired product was isolated as a light yellow oil (87.7 mg, 90%). R_f (petroleum ether/ethyl acetate = 9:1): 0.47. **^{1}H NMR** (400.2 MHz, $CDCl_3$) δ 7.47 (d, J = 8.4 Hz, 2H), 7.39 (d, J = 8.4 Hz, 2H), 3.89 – 3.83 (m, 4H), 3.01 – 2.96 (m, 4H). **^{19}F NMR** (376.6 MHz, $CDCl_3$) δ –36.7 (s, 3F). **^{13}C NMR** (100.6 MHz, $CDCl_3$) δ 156.4, 136.6, 132.9, 129.8, 128.9 (q, J = 310.9 Hz), 128.5, 65.9, 54.3. **IR** (neat, cm^{-1}) ν: 2965, 2894, 1590, 1489, 1455, 1244, 1135, 1100, 975, 826, 713, 631, 448. **HRMS** (EI^+) calcd for $C_{12}H_{12}ClF_3N_2OS$ *m/z* 324.0311 $[M]^+$, Found 324.0310 (Δ = –0.33 ppm).

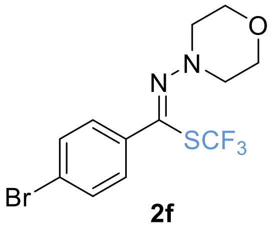

2f

Trifluoromethyl (*Z*)-4-bromo-*N*-morpholinobenzimidothioate (2f): The product was purified by silica gel flash column chromatography eluting with petroleum ether/ethyl acetate (8:2). The desired product was isolated as a yellow solid (82.0 mg, 75%). mp: 42 – 44 °C. R_f (petroleum ether/ethyl acetate = 8:2): 0.33. **^{1}H NMR** (400.2 MHz, $CDCl_3$) δ 7.55 (d, J = 8.4 Hz, 2H), 7.40 (d, J = 8.4 Hz, 2H), 3.89 – 3.82 (m, 4H), 3.01 – 2.95 (m, 4H). **^{19}F NMR** (376.6 MHz, $CDCl_3$) δ –36.7 (s, 3F). **^{13}C NMR** (100.6 MHz, $CDCl_3$) δ 156.1, 133.4, 131.4, 130.0, 128.9 (q, J = 310.9), 124.9, 65.9, 54.3. **IR** (neat, cm^{-1}) ν: 2964, 2894, 1455, 1393, 1244, 1135, 1098, 826, 724, 628, 496. **HRMS** (EI^+) calcd for $C_{12}H_{12}BrF_3N_2OS$ *m/z* 367.9806 $[M]^+$, Found 367.9811 (Δ = 1.41 ppm).

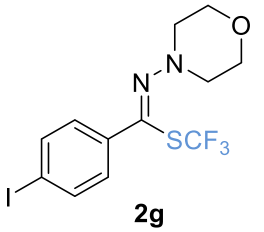

2g

Trifluoromethyl (*Z*)-4-iodo-*N*-morpholinobenzimidothioate (2g): The product was purified by silica gel flash column chromatography eluting with petroleum ether/ethyl acetate (8:2). The desired product was isolated as a white and slightly orange solid (103.0 mg, 83%). mp: 89 – 91 °C. R_f (petroleum ether/ethyl acetate = 8:2): 0.54. ^{1}**H NMR** (400.2 MHz, $CDCl_3$) δ 7.79 – 7.73 (m, 2H), 7.29 – 7.23 (m, 2H), 3.93 – 3.81 (m, 4H), 3.05 – 2.96 (m, 4H). ^{19}**F NMR** (376.6 MHz, $CDCl_3$) δ –36.7 (s, 3F). ^{13}**C NMR** (100.6 MHz, $CDCl_3$) δ 156.0, 137.3, 134.0, 130.0, 128.8 (q, *J* = 311.0 Hz), 96.8, 65.9, 54.3. **IR** (neat, cm^{-1}) ν: 2966, 2838, 1698, 1591, 1455, 1261, 1138, 1099, 976, 941, 822, 721, 625, 518, 460, 449. **HRMS** (EI^+) calcd for $C_{12}H_{12}IF_3N_2OS$ *m/z* 415.9667 $[M]^+$, Found 415.9668 (Δ = 0.25 ppm).

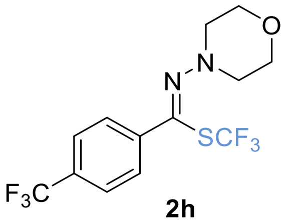

2h

Trifluoromethyl (*Z*)-*N*-morpholino-4-(trifluoromethyl)benzimidothioate (2h): The product was purified by silica gel flash column chromatography eluting with petroleum ether/ethyl acetate (9:1). The desired product was isolated as an amorphous yellow solid (97.8 mg, 91%). mp: 42 – 44 °C. R_f (petroleum ether/ethyl acetate = 9:1): 0.48. ^{1}**H NMR** (400.2 MHz, $CDCl_3$) δ 7.70 – 7.63 (m, 4H), 3.91 – 3.83 (m, 4H), 3.08 – 3.01 (m, 4H). ^{19}**F NMR** (376.6 MHz, $CDCl_3$) δ –36.7 (s, 3F), –63.4 (s, 3F). ^{13}**C NMR** (100.6 MHz, $CDCl_3$) δ 153.7, 138.2, 132.6 (q, *J* = 32.8 Hz), 128.8, 128.8, (q, *J* = 310.8 Hz), 125.2 (q, *J* = 3.7 Hz), 123.7 (q, *J* = 272.3 Hz), 65.9, 54.3. **IR** (neat, cm^{-1}) ν: 2968, 2857, 1618, 1589, 1407, 1323, 1126, 1100, 977, 844, 713, 689, 634, 500. **HRMS** (EI^+) calcd for $C_{13}H_{12}F_6N_2OS$ *m/z* 358.0575 $[M]^+$, Found 358.0576 (Δ = 0.31 ppm).

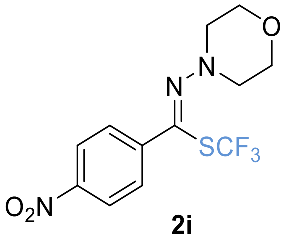

2i

Trifluoromethyl (*Z*)-*N*-morpholino-4-nitrobenzimidothioate (2i): The product was purified by silica gel flash column chromatography eluting with petroleum ether/ethyl acetate (9:1). The desired product was isolated as an amorphous orange solid (92.4 mg, 92%). mp: 43 – 45 °C. R_f (petroleum ether/ethyl acetate = 9:1): 0.48. ^{1}**H NMR** (400.2 MHz, $CDCl_3$) δ 8.26 (d, *J* = 8.8 Hz, 2H), 7.79 (d, *J* = 8.8 Hz, 2H), 3.90 – 3.82 (m, 4H), 3.29 – 3.20 (m, 4H). ^{19}**F NMR** (376.6 MHz, $CDCl_3$) δ –37.0 (s, 3F). ^{13}**C NMR** (100.6 MHz, $CDCl_3$) δ 148.3, 144.4, 141.9, 129.0, 128.6 (q, *J* = 311.3 Hz), 123.4, 65.9, 54.4. **IR** (neat, cm^{-1}) ν: 2967, 2858, 1599, 1520, 1341, 1139, 976, 908, 849, 730, 752, 693, 439. **HRMS** (EI^+) calcd for $C_{12}H_{12}F_3N_3O_3S$ *m/z* 335.0551 $[M]^+$, Found 335.0561 (Δ = 2.72 ppm).

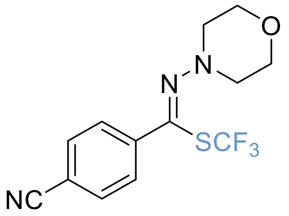

2j

Trifluoromethyl (*Z*)-4-cyano-*N*-morpholinobenzimidothioate (2j): The product was purified by silica gel flash column chromatography eluting with petroleum ether/ethyl acetate (9:1). The desired product was isolated as an amorphous orange solid (81.2 mg, 86%). R_f (petroleum ether/ethyl acetate = 9:1): 0.52. **^{1}H NMR** (400.2 MHz, $CDCl_3$) δ 7.75 – 7.66 (m, 4H), 3.91 – 3.84 (m, 4H), 3.21 – 3.14 (m, 4H). **^{19}F NMR** (376.6 MHz, $CDCl_3$) δ –36.9 (s, 3F). **^{13}C NMR** (100.6 MHz, $CDCl_3$) δ 147.5, 139.8, 132.0, 128.8, 128.6 (q, J = 311.5 Hz), 118.1, 113.5, 65.9, 54.4. **IR** (neat, cm^{-1}) ν: 2946, 2837, 2231, 1587, 1459, 1237, 1153, 1106, 977, 842, 556, 465, 414. **HRMS** (EI^+) calcd for $C_{13}H_{12}F_3N_3OS$ *m/z* 315.0653 $[M]^+$, Found 315.0659 (Δ = 1.86 ppm).

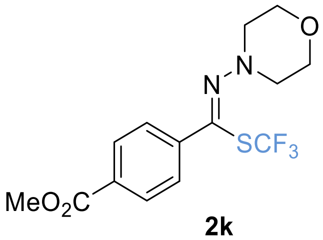

2k

Methyl (*Z*)-4-((morpholinoimino)((trifluoromethyl)thio)methyl)benzoate (2k): The product was purified by silica gel flash column chromatography eluting with petroleum ether/ethyl acetate (8:2). The desired product was isolated as an orange solid (95.0 mg, 91%). mp: 49 – 51 °C. R_f (petroleum ether/ethyl acetate = 8:2): 0.54. **^{1}H NMR** (400.2 MHz, $CDCl_3$) δ 8.10 – 8.05 (m, 2H), 7.63 – 7.57 (m, 2H), 3.93 (s, 1H), 3.89 – 3.83 (m, 4H), 3.06 – 2.99 (m, 4H). **^{19}F NMR** (376.6 MHz, $CDCl_3$) δ –36.8 (s, 3F). **^{13}C NMR** (100.6 MHz, $CDCl_3$) δ 166.3, 154.8, 138.7, 131.6, 129.4, 128.8 (q, J = 311.1 Hz), 128.5, 65.9, 54.3, 52.3. **IR** (neat, cm^{-1}) ν: 2955, 2861, 1726, 1590, 1438, 1278, 1140, 1102, 947, 862, 713, 632, 439. **HRMS** (EI^+) calcd for $C_{14}H_{15}F_3N_2O_3S$ *m/z* 348.0755 $[M]^+$, Found 348.0758 (Δ = 0.72 ppm).

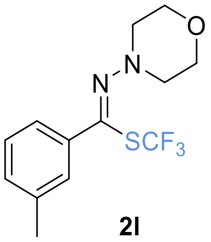

2l

Trifluoromethyl (*Z*)-3-methyl-*N*-morpholinobenzimidothioate (2l): The product was purified by silica gel flash column chromatography eluting with petroleum ether/ethyl acetate (9:1). The desired product was isolated as a light orange amorphous solid (79.4 mg, 87%). R_f (petroleum ether/ethyl acetate = 9:1): 0.56. **^{1}H NMR** (400.2 MHz, $CDCl_3$) δ 7.32 – 7.25 (m, 4H), 3.89 – 3.84 (m, 4H), 2.98 – 2.92 (m, 4H), 2.38 (s, 3H). **^{19}F NMR** (376.6 MHz, $CDCl_3$) δ –36.9 (s, 3F). **^{13}C NMR** (100.6 MHz, $CDCl_3$) δ 159.8, 137.9, 133.7, 130.6, 129.4 (q, J = 310.7), 128.9, 128.0, 125.6, 65.9, 54.2, 21.2. **IR** (neat, cm^{-1}) ν: 2853, 1566, 1451, 1152, 1100, 973, 861, 702, 626, 450. **HRMS** (EI^+) calcd for $C_{13}H_{15}F_3N_2OS$ *m/z* 304.0857 $[M]^+$, Found 304.0861 (Δ = 1.26 ppm).

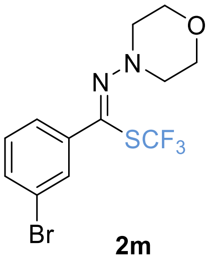

2m

Trifluoromethyl (*Z*)-3-bromo-*N*-morpholinobenzimidothioate (2m): The product was purified by silica gel flash column chromatography eluting with petroleum ether/ethyl acetate (8:2). The desired product was isolated as a yellow oil (98.5 mg, 89%). R_f (petroleum ether/ethyl acetate = 8:2): 0.41. **^{1}H NMR** (400.2 MHz, $CDCl_3$) δ 7.68 (s, 1H), 7.60 – 7.57 (m, 1H), 7.47 (d, *J* = 8.0, 1H), 7.28 (t, *J* = 8.0, 1H), 3.89 – 3.82 (m, 4H), 3.04 – 2.98 (m, 4H). **^{19}F NMR** (376.6 MHz, $CDCl_3$) δ –36.7 (s, 3F). **^{13}C NMR** (100.6 MHz, $CDCl_3$) δ 154.9, 136.3, 131.3, 130.4, 129.6, 128.8 (q, *J* = 311.1 Hz), 137.1, 122.1, 65.9, 54.3. **IR** (neat, cm^{-1}) ν: 2965, 2855, 1581, 1562, 1455, 1262, 1135, 1103, 863, 692, 655, 500. **HRMS** (EI^+) calcd for $C_{12}H_{12}BrF_3N_2OS$ *m/z* 367.9806 $[M]^+$, Found 367.9814 (Δ = 2.31 ppm).

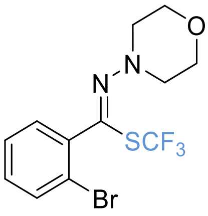

2n

Trifluoromethyl (*Z*)-2-bromo-*N*-morpholinobenzimidothioate (2n): The product was purified by silica gel flash column chromatography eluting with petroleum ether/ethyl acetate (8:2). The desired product was isolated as a light-yellow oil (86.1 mg, 78%). R_f (petroleum ether/ethyl acetate = 8:2): 0.39. **^{1}H NMR** (400.2 MHz, $CDCl_3$) δ 7.63 – 7.61 (m, 1H), 7.41 – 7.36 (m, 2H), 7.35 – 7.29 (m, 1H), 3.90 – 3.82 (m, 4H), 3.00 – 2.92 (m, 4H). **^{19}F NMR** (376.6 MHz, $CDCl_3$) δ –38.4 (s, 3F). **^{13}C NMR** (100.6 MHz, $CDCl_3$) δ 161.5, 133.9, 132.7, 131.5, 130.5, 129.0 (q, *J* = 311.0 Hz), 126.9, 122.9, 65.8, 53.9. **IR** (neat, cm^{-1}) ν: 2964, 2891, 1601, 1583, 1434, 1262, 1234, 1136, 1104, 975, 943, 860, 760, 690, 650, 469, 459. **HRMS** (EI^+) calcd for $C_{12}H_{12}BrF_3N_2OS$ *m/z* 367.9806 $[M]^+$, Found 367.9811 (Δ = 1.48 ppm).

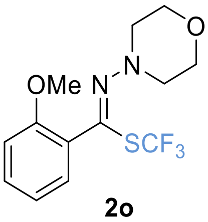

2o

Trifluoromethyl (Z)-2-methoxy-*N*-morpholinobenzimidothioate (2o): The product was purified by silica gel flash column chromatography eluting with petroleum ether/ethyl acetate (8:2). The desired product was isolated as a colorless solid (60.5 mg, 63%). mp: 69 – 71 °C. R_f (petroleum ether/ethyl acetate = 8:2): 0.39. **^{1}H NMR** (400.2 MHz, $CDCl_3$) δ 7.44 – 7.37 (m, 1H), 7.25 (d, *J* = 7.1 Hz, 1H), 6.98 (td, J = 7.5, 0.7 Hz, 1H), 6.91 (d, J = 8.4 Hz, 1H), 3.87 – 3.84 (m, 4H), 3.82 (s, 3H), 2.95 – 2.93 (m, 4H). **^{19}F NMR** (376.6 MHz, $CDCl_3$) δ –39.4 (s, 3F). **^{13}C NMR** (100.6 MHz, $CDCl_3$) δ 160.9, 157.3, 131.7, 130.0 129.3 (q, *J* = 310.5 Hz), 122.3, 120.0, 110.8, 66.0, 55.6, 54.1. **IR** (neat, cm^{-1}) ν: 2962, 2857, 1593, 1492, 1258, 1131, 1105, 1022, 974, 753, 571, 384. **HRMS** (EI^+) calcd for $C_{13}H_{15}F_3N_2O_2S$ *m/z* 320.0806 $[M]^+$, Found 320.0797 (Δ = –2.92 ppm).

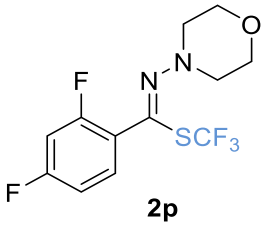

2p

Trifluoromethyl (*Z*)-2,4-difluoro-*N*-morpholinobenzimidothioate (2p): The product was purified by silica gel flash column chromatography (height 12 cm, width 2 cm) eluting with *n*-pentane/ethyl acetate (97:03 to 95:05). The desired product was isolated as a colorless oil (77.3 mg, 79%). R_f (*n*-pentane/ethyl acetate = 95:05): 0.39. ^{1}**H NMR** (400.2 MHz, $CDCl_3$) δ 7.39 – 7.31 (m, 1H), 6.98 – 6.85 (m, 2H), 3.89 – 3.83 (m, 4H), 2.95 – 2.90 (m, 4H). ^{19}**F NMR** (376.6 MHz, $CDCl_3$) δ –38.7 (d, *J* = 3.9 Hz, 3F), –105.7 (d, *J* = 9.6 Hz, 1F), –110.3 (dq, *J* = 7.9, 4.0 Hz, 1F). ^{13}**C NMR** (100.6 MHz, $CDCl_3$) δ 164.3 (dd, *J* = 253.3, 12.5 Hz), 160.5 (dd, *J* = 254.0, 12.5 Hz), 156.8, 131.7 (dd, *J* = 10.5, 3.4 Hz), 129.1 (q, *J* = 310.8 Hz), 117.9 (dd, *J* = 15.9, 4.0 Hz), 111.4 (dd, *J* = 22.0, 3.7 Hz), 104.3 (dd, *J* = 25.8, 24.7 Hz), 65.9, 54.1. **IR** (neat, cm^{-1}) ν: 2964, 2856, 1621, 1505, 1136, 1112, 975, 858, 821, 730, 533, 431. **HRMS** (ESI^+) calcd for $C_{12}H_{12}ON_2F_5S$ *m/z* 327.0585 $[M+H]^+$, Found 327.0588 (Δ = 0.95 ppm).

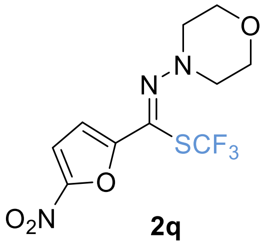

2q

Trifluoromethyl (*Z*)-*N*-morpholino-5-nitrofuran-2-carbimidothioate (2q): The product was purified by silica gel flash column chromatography eluting with petroleum ether/ethyl acetate (8:2). The desired product was isolated as a golden brown solid (87.8 mg, 90%). mp: 101 – 103 °C. R_f (petroleum ether/ethyl acetate = 8:2): 0.39. ^{1}**H NMR** (400.2 MHz, $CDCl_3$) δ 7.33 (d, *J* = 3.9 Hz, 1H), 6.76 (d, *J* = 3.9 Hz, 1H), 3.96 – 3.92 (m, 4H), 3.88 – 3.85 (m, 4H). ^{19}**F NMR** (376.6 MHz, $CDCl_3$) δ –39.8 (s, 3F). ^{13}**C NMR** (100.6 MHz, $CDCl_3$) δ 155.5, 151.5, 128.4 (q, *J* = 312.6 Hz), 113.8, 110.1, 104.3, 66.2, 54.3. **IR** (neat, cm^{-1}) ν: 3106, 2855, 1541, 1477, 1345, 1245, 1152, 1106, 990, 866, 800, 612, 490, 440. **HRMS** (EI^+) calcd for $C_{10}H_{10}F_3N_3O_4S$ *m/z* 325.0344 $[M]^+$, Found 325.0330 (Δ = –4.30 ppm).

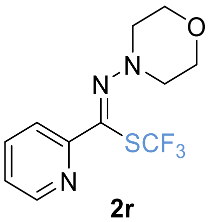

2r

Trifluoromethyl (*Z*)-*N*-morpholinopyridine-2-carbimidothioate (2r): The product was purified by silica gel flash column chromatography eluting with petroleum ether/ethyl acetate (95:5). The desired product was isolated as a colorless oil (13.1 mg, 15%). R_f (petroleum ether/ethyl acetate = 95:5): 0.49. ^{1}**H NMR** (400.2 MHz, $CDCl_3$) δ 8.66 (m, 1H), 7.76 (td, *J* = 7.7, 1.7 Hz, 1H), 7.64 (d, *J* = 7.9 Hz, 1H), 7.35 (m, 1H), 3.90 – 3.83 (m, 4H), 3.16 – 3.10 (m, 4H). ^{19}**F NMR** (376.6 MHz, $CDCl_3$) δ –36.9. ^{13}**C NMR** (100.6 MHz, $CDCl_3$) δ 153.2, 151.1, 149.1, 136.7, 128.0 (q, *J* = 311.2 Hz), 124.4, 123.1, 66.0, 54.3. **IR** (neat, cm^{-1}) ν: 2965, 2849, 1583, 1456, 1262, 1098, 977, 862, 755, 697, 629, 405. **HRMS** (EI^+) calcd for $C_{11}H_{12}F_3N_3OS$ *m/z* 291.0653 $[M]^+$, Found 291.0653 (Δ = 0.01 ppm).

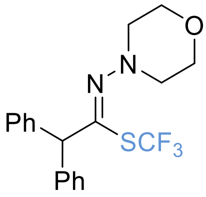

2s

Trifluoromethyl (*Z*)-*N*-morpholino-2,2-diphenylethanimidothioate (2s): The product was purified by silica gel flash column chromatography eluting with petroleum ether/ethyl acetate (95:5). The desired product was isolated as a colorless oil (79.3 mg, 69%). R_f (petroleum ether/ethyl acetate = 95:5): 0.49. **^{1}H NMR** (400.2 MHz, $CDCl_3$) δ 7.37 – 7.23 (m, 10H), 5.46 (s, 1H), 3.83 – 3.76 (m, 4H), 2.85 – 2.77 (m, 4H). **^{19}F NMR** (376.6 MHz, $CDCl_3$) δ –37.5 (s, 3F). **^{13}C NMR** (100.6 MHz, $CDCl_3$) δ 164.3, 139.6, 129.4 (q, J = 310.7 Hz), 128.9, 128.4, 65.7, 55.0, 53.8. **IR** (neat, cm^{-1}) ν: 2964, 2846, 1703, 1450, 1261, 1150, 1109, 979, 881, 864, 731, 634, 419. **HRMS** (EI$^+$) calcd for $C_{19}H_{19}F_3N_2OS$ *m/z* 380.1170 [M]$^+$, Found 380.1174 (Δ = 1.07 ppm).

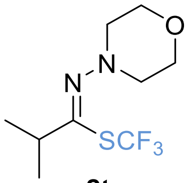

2t

Trifluoromethyl (*Z*)-2-methyl-*N*-morpholinopropanimidothioate (2t): The product was purified by silica gel flash column chromatography eluting with petroleum ether/ethyl acetate (9:1). The desired product was isolated as a colorless oil (23.8 mg, 31%). R_f (petroleum ether/ethyl acetate = 9:1): 0.47. **^{1}H NMR** (400.2 MHz, $CDCl_3$) δ 3.83 – 3.73 (m, 4H), 2.91 (hept, J = 6.7 Hz, 1H). 2.76 – 2.70 (m, 4H), 1.29 (d, J = 6.7 Hz, 6H). **^{19}F NMR** (376.6 MHz, $CDCl_3$) δ –38.6 (s, 3F). **^{13}C NMR** (100.6 MHz, $CDCl_3$) δ 169.4, 128.9 (q, J = 310.9 Hz), 65.9, 53.9, 33.6 (q, J = 2.1 Hz), 21.8. **IR** (neat, cm^{-1}) ν: 2970, 2844, 1604, 1457, 1262, 1106, 973, 861, 693, 565, 458. **HRMS** (EI$^+$) calcd for $C_9H_{15}F_3N_2OS$ *m/z* 256.0857 [M]$^+$, Found 256.0853 (Δ = –1.66 ppm).

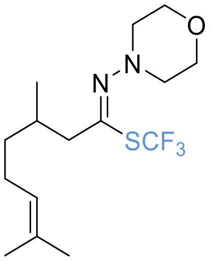

2u

Trifluoromethyl (*Z*)-3,7-dimethyl-*N*-morpholinooct-6-enimidothioate (2u): The product was purified by silica gel flash column chromatography eluting with petroleum ether/ethyl acetate (95:5). The desired product was isolated as a colorless oil (39.6 mg, 39%). R_f (petroleum ether/ethyl acetate = 95:5): 0.56. **^{1}H NMR** (400.2 MHz, $CDCl_3$) δ 5.10 – 5.06 (m, 1H), 3.81 – 3.76 (m, 4H), 2.78 – 2.74 (m, 4H), 2.63 (dd, J = 15.8, 5.8 Hz, 1H), 2.43 (dd, J = 15.8, 7.9 HZ, 1H), 2.12 – 1.91 (m, 3H), 1.68 (s, 3H), 1.59 (s 3H), 1.48 – 1.39 (m, 1H), 1.27 – 1.20 (m, 1H), 0.96 (d, J = 6.6 Hz, 3H). **^{19}F NMR** (376.6 MHz, $CDCl_3$) δ –38.1 (s, 3F). **^{13}C NMR** (100.6 MHz, $CDCl_3$) δ 163.6, 131.8, 130.3 (q, J = 310.5 Hz), 124.3, 65.8, 54.0, 41.4 (q, J = 2.2 Hz), 36.7, 30.9, 25.7, 25.3, 19.1, 17.6. **IR** (neat, cm^{-1}) ν: 2965, 2856, 1597, 1456, 1262, 1156, 1120, 975, 947, 869, 727, 623, 465. **HRMS** (EI$^+$) calcd for $C_{14}H_{25}N_2OS$ *m/z* 269.1688 [M-CF_3]$^+$, Found 269.1696 (Δ = 3.42 ppm).

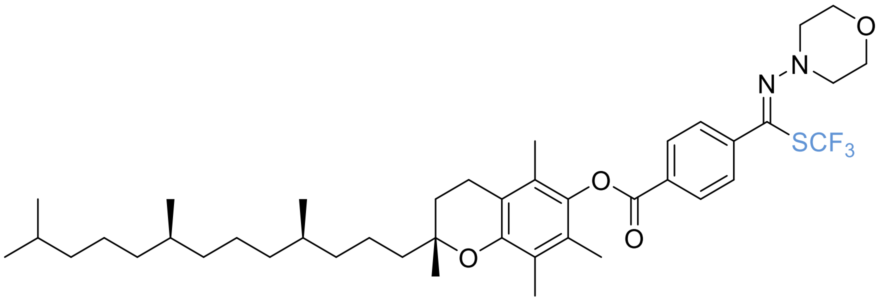

2v

(*R*)-2,5,7,8-Tetramethyl-2-((4*R*,8*R*)-4,8,12-trimethyltridecyl)chroman-6-yl 4-((*Z*)-(morpholinoimino)((trifluoromethyl)thio)methyl)benzoate (2v): The product was purified by silica gel flash column chromatography (height 12 cm, width 2 cm) eluting with *n*-pentane/ethyl acetate (97:03 to 94:06). The desired product was isolated with an inseparable impurity as a yellow oil (115 mg, 52%). R_f (*n*-pentane/ethyl acetate = 94:06): 0.39. **^{1}H NMR** (400.2 MHz, $CDCl_3$) δ 8.29 (d, J = 8.4, 2H), 7.71 (d, J = 8.1 Hz, 2H), 3.92 – 3.87 (m, 4H), 3.12 – 3.06 (m, 4H), 2.63 (t, J = 6.8 Hz, 2H), 2.14 (s, 3H), 2.06 (s, 3H), 2.02 (s, 3H), 1.89 – 1.75 (m, 2H), 1.60 – 1.48 (m, 3H), 1.47 – 1.36 (m, 4H), 1.32 – 1.22 (m, 10H), 1.19 – 1.04 (m, 7H), 0.92 – 0.86 (m, 12H).**^{19}F NMR** (376.6 MHz, $CDCl_3$) δ –36.6 (s, 3F). **^{13}C NMR** (100.6 MHz, $CDCl_3$) δ 164.4, 153.7, 149.6, 140.5, 139.5, 131.0, 129.9, 128.9 (q, J = 311.9 Hz), 128.6, 126.8, 125.0, 123.2, 117.5, 75.1, 65.9, 54.4, 39.3, 37.5, 37.4, 37.4, 37.3, 32.8, 32.8, 32.7, 28.0, 24.8, 24.4, 22.7, 22.6, 21.0, 20.6, 19.7, 19.7, 19.6, 19.6, 13.0, 12.2, 11.8. **IR** (neat, cm^{-1}) ν: 2925, 2857, 1736, 1456, 1378, 1234, 1140, 1104, 977, 862, 628. **HRMS** (ESI^+) calcd for $C_{42}H_{61}O_4N_2F_3SNa$ *m/z* 769.4196 $[M+Na]^+$, Found 769.4200 (Δ = 0.53 ppm).

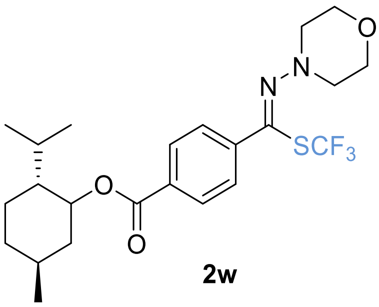

2w

(2*R*,5*S*)-2-Isopropyl-5-methylcyclohexyl 4-((*Z*)-(morpholinoimino)((trifluorometh-yl)thio)methyl)benzoate (2w): The product was purified by silica gel flash column chromatography (height 12 cm, width 2 cm) eluting with *n*-pentane/ethyl acetate (97:03 to 95:05). The desired product was isolated as a yellow oil (101 mg, 72%). R_f (*n*-pentane/ethyl acetate = 95:05): 0.45. **^{1}H NMR** (400.2 MHz, $CDCl_3$) δ 8.07 (d, J = 8.4 Hz, 2H), 7.60 (d, J = 8.2 Hz, 2H), 4.94 (td, J = 8.0, 4.4 Hz, 1H), 3.90 – 3.83 (m, 4H), 3.06 – 3.00 (m, 4H), 2.16 – 2.09 (m, 1H), 1.98 – 1.88 (m, 1H), 1.77 – 1.69 (m, 2H), 1.61 – 1.51 (m, 2H), 1.19 – 1.06 (m, 2H), 0.98 – 0.88 (m, 7H), 0.79 (d, J = 8.1 Hz, 3H). **^{19}F NMR** (376.6 MHz, $CDCl_3$) δ –36.7 (s, 3F). **^{13}C NMR** (100.6 MHz, $CDCl_3$) δ 165.3, 154.9, 138.6, 132.3, 129.3, 128. 9 (q, J = 311.9 Hz), 128.4, 75.3, 65.9, 54.3, 47.2, 40.9, 34.3, 31.4, 26.5, 23.6, 22.0, 20.7, 16.5. **IR** (neat, cm^{-1}) ν: 2957, 2857, 1714, 1456, 1272, 1139, 1101, 978, 864, 711, 631, 466. **HRMS** (ESI^+) calcd for $C_{23}H_{32}O_3N_2F_3S$ *m/z* 473.2080 $[M+H]^+$, Found 473.2082 (Δ = 0.5 ppm).

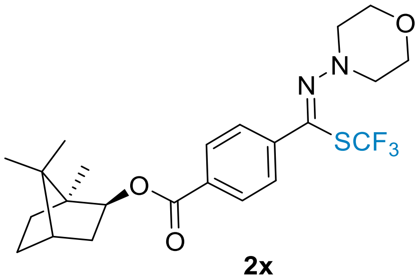

2x

(1*S*,2*S*,4*S*)-1,7,7-Trimethylbicyclo[2.2.1]heptan-2-yl 4-((*Z*)-(morpholinoimino)((trifluoro-methyl)thio)methyl)benzoate (2x): The product was purified by silica gel flash column chromatography (height 12 cm, width 2 cm) eluting with *n*-pentane/ethyl acetate (97:03 to 95:05). The desired product was isolated as a yellow oil (91 mg, 65%). R_f (*n*-pentane/ethyl acetate = 95:05): 0.45. **^{1}H NMR** (400.2 MHz, $CDCl_3$) δ 8.11 – 8.04 (m, 2H), 7.62 (d, J = 8 Hz, 2H), 5.15 – 5.10 (m, 1H), 3.9 – 3.84 (m, 4H), 3.07 – 3.01 (m, 4H), 2.53 – 2.43 (m, 1H), 2.15 – 2.07 (m, 1H), 1.87 – 1.77 (m, 1H), 1.75 (t, J = 4 Hz, 1H), 1.47 – 1.37 (m, 1H), 1.36 – 1.27 (m, 1H), 1.13 (dd, J = 16, 4 Hz, 1H), 0.97 (s, 3H), 0.92 (d, J = 1.8 Hz, 6H). **^{19}F NMR** (376.6 MHz, $CDCl_3$) δ –36.7 (s, 3F). **^{13}C NMR** (100.6 MHz, $CDCl_3$) δ 166.0, 154.7, 138.7, 132.3, 129.3, 128.9 (q, J = 311.9 Hz), 128.5, 81.0, 65.9, 54.4, 49.1, 47.9, 44.9, 36.8, 28.1, 27.4, 19.7, 18.9, 13.6. **IR** (neat, cm^{-1}) ν: 2956, 1717, 1587, 1455, 1301, 1272, 1101, 947, 755, 631, 464. **HRMS** (ESI^+) calcd for $C_{23}H_{30}O_3N_2F_3S$ *m/z* 471.1923 $[M+H]^+$, Found 471.1927 (Δ = 0.67 ppm).

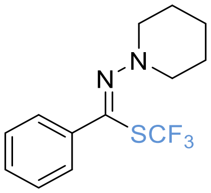

3a

Trifluoromethyl (*Z*)-*N*-(piperidin-1-yl)benzimidothioate (3a): The product was purified by silica gel flash column chromatography (height 12 cm, width 2 cm) eluting with *n*-pentane/ethyl acetate (97:03 to 95:05). The desired product was isolated as a colorless oil (57 mg, 66%). R_f (*n*-pentane/ethyl acetate = 95:05): 0.48. **^{1}H NMR** (400.2 MHz, $CDCl_3$) δ 7.51 – 7.35 (m, 5H), 2.89 – 2.82 (m, 4H), 1.79 – 1.70 (m, 4H), 1.54 – 1.46 (m, 2H). **^{19}F NMR** (376.6 MHz, $CDCl_3$) δ –37.2 (s, 3F). **^{13}C NMR** (100.6 MHz, $CDCl_3$) δ 159.0, 134.3, 130.0, 129.4 (q, J = 311.1 Hz), 128.5, 128.0, 55.3, 25.0, 23.5. **IR** (neat, cm^{-1}) ν: 2940, 2827, 1445, 1132, 1103, 948, 752, 696, 460. **HRMS** (ESI^+) calcd for $C_{13}H_{16}N_2F_3S$ *m/z* 289.0980 $[M+H]^+$, Found 289.0982 (Δ = 0.26 ppm).

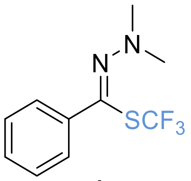

4a

Trifluoromethyl (*Z*)-*N*,*N*-dimethylbenzohydrazonothioate (4a): The product was purified by silica gel flash column chromatography eluting with *n*-pentane/ethyl acetate (100:0 to 98:2). The desired product was isolated with an inseparable impurity as a colorless oil (57.3 mg, 77%). R_f (*n*-pentane/ethyl acetate = 98:2): 0.54. **^{1}H NMR** (400.2 MHz, $CDCl_3$) δ 7.62 – 7.52 (m, 2H), 7.43 – 7.36 (m, 3H), 2.77 (s, 6H). **^{19}F NMR** (376.6 MHz, $CDCl_3$) δ –37.5 (s, 3F). **^{13}C NMR** (100.6 MHz, $CDCl_3$) δ 150.7, 135.4, 129.7, 129. 2 (q, J = 311.0 Hz), 128.2, 128.1, 46.6. **IR** (neat, cm^{-1}) ν: 2925, 2855, 1536, 1465, 1138, 1110, 754, 696, 464, 398. **HRMS** (ESI^+) calcd for $C_{10}H_{12}N_2F_3S$ *m/z* 249.0667 $[M+H]^+$, Found 249.0668 (Δ = 0.28 ppm).

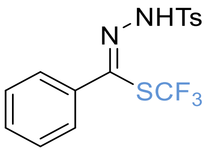

5a

Trifluoromethyl (*Z*)-*N*-tosylbenzohydrazonothioate (5a): The product was purified by silica gel flash column chromatography (height 12 cm, width 2 cm) eluting with *n*-pentane/ethyl acetate (97:03 to 95:05). The desired product was isolated with an inseparable impurity as a white solid (60.8 mg, 55%). mp: 148 – 150 °C. R_f (*n*-pentane/ethyl acetate = 95:05): 0.46. **^{1}H NMR** (400.2 MHz, $CDCl_3$) δ 8.97 (s, 1H), 7.90 – 7.86 (m, 2H), 7.85 – 7.81 (m, 2H), 7.45 – 7.36 (m, 3H), 7.34 (d, J = 8.1 Hz, 2H), 2.43 (s, 3H). **^{19}F NMR** (376.6 MHz, $CDCl_3$) δ –36.8 (s, 3F). **^{13}C NMR** (100.6 MHz, $CDCl_3$) δ 144.9, 135.1, 135.0, 133.2, 130.8, 129.9, 128.6, 127.9, 127.5 (q, J = 311.9 Hz), 127.8, 21.6. **IR** (neat, cm^{-1}) ν: 3176, 2922, 1598, 1385, 1350, 1170, 1134, 1070, 767, 671, 551, 432. **HRMS** (ESI^+) calcd for $C_{15}H_{14}O_2N_2F_3S_2$ *m/z* 375.0443 $[M+H]^+$, Found 375.0443 (Δ = -0.09 ppm).

8. Reluctant substrates

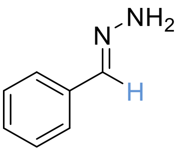

ND

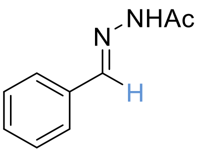

ND

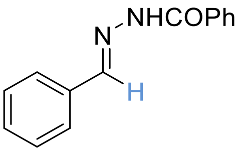

ND

In all cases, the triflouromethylthiolated products were not detected (ND).

9. Mechanistic studies

Study on the role of the $(SCF_3)_2$ dimer

First, the $(SCF_3)_2$ dimer was prepared in 5 minutes according to the literature.[1] Then, the dimer was added as THF solution to the reaction mixture while keeping the same concentration (0.1 M).

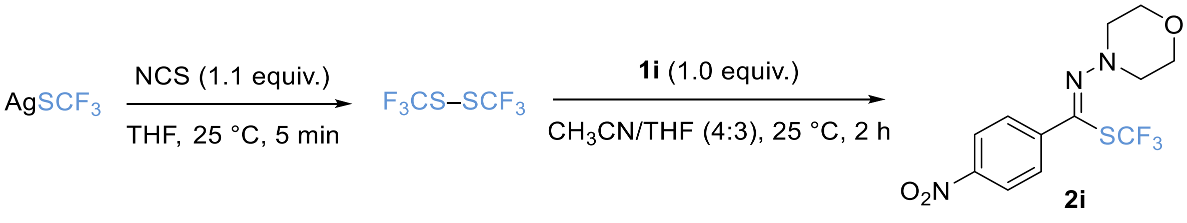

0% ^{19}F NMR yield

Using a CH_3CN/THF (4:3) as a solvent:

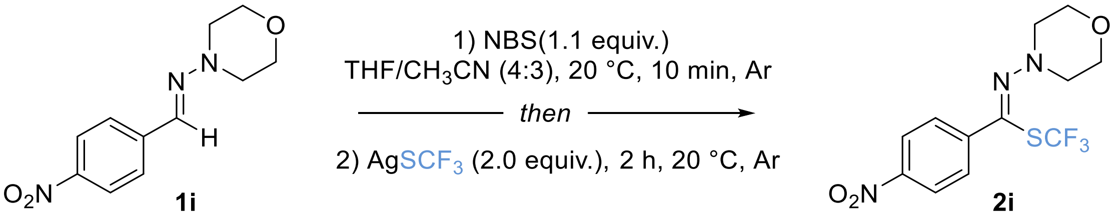

82%, (^{19}F NMR yield)

An oven-dried 10 mL tube A equipped with a stirring bar was charged with $AgSCF_3$ (314 mg, 1.5 mmol), NCS (200 mg, 1.5 mmol) and THF (4 mL), under argon atmosphere. The resulting reaction system was stirred at 25 °C for 5 min. A small portion (0.1 mL) was collected and α,α,α-trifluoroacetophenone (14 µL, 0.1 mmol, 1.0 equiv) was added as an internal standard for determining the dimer concentration of the resulting solution by ^{19}F NMR analysis. Another oven-dried 10 mL tube B equipped with a stirring bar was charged with the *p*-nitrophenylhydrazone derivative **1i** (70.6 mg, 0.3 mmol, 1.0 equiv) and CH_3CN (1.2 mL). Then, 1.6 mL of the reaction mixture of tube A [containing $(SCF_3)_2$ (0.6 mmol, 2 equiv)] was added into the reaction mixture of tube B. The mixture was stirred at room temperature for 2 h. α,α,α-trifluoroacetophenone (42 µL, 0.3 mmol, 1.0 equiv) was added as an internal standard for determining the ^{19}F NMR yield. No product was detected. In comparison, a control experiment using the same mixture of solvents was used and **2i** was obtained in 82% ^{19}F NMR yield.

An oven-dried 10 mL tube equipped with a stirring bar was charged with the *p*-nitrophenylhydrazone derivative **1i** (70.6 mg, 0.3 mmol, 1.0 equiv) and a 4:3 mixture of THF (1.6 mL) and CH_3CN (1.2 mL) was added. Then, the mixture was stirred until the solubilization of the reagent. Then, recrystallized NBS (58.7 mg, 0.33 mmol, 1.1 equiv) was added, and the reaction mixture was stirred for 5–10 minutes. After which, $AgSCF_3$ (125.0 mg, 0.6 mmol, 2.0 equiv) was added. The reaction mixture was stirred for another 2 hours at room temperature. α,α,α-Trifluoroacetophenone (42 µL, 0.3 mmol, 1.0 equiv) was added as an internal standard for determining the ^{19}F NMR yield.

The $(SCF_3)_2$ dimer might not be the active species in the process.

Test with SCF_3 succinimide as a SCF_3 source

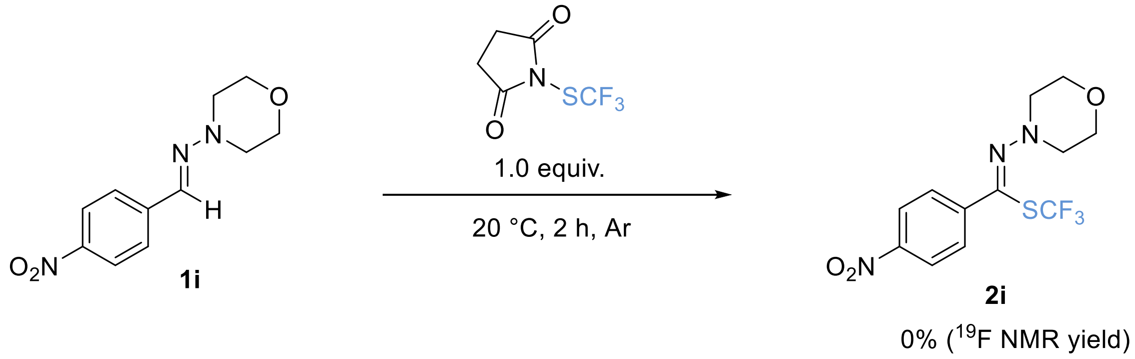

An oven-dried 10 mL reaction tube equipped with a stirring bar was charged with the hydrazone derivative **1i** (0.3 mmol, 1.0 equiv) and CH_3CN (0.7 mL). The mixture was stirred until solubilization of the reagent. Then, N-SCF_3 succinimide (119.5 mg, 0.6 mmol, 2 equiv) was added, and the reaction mixture was stirred for 2 hours at room temperature. α,α,α-Trifluoroacetophenone (42 µL, 0.3 mmol, 1.0 equiv) was added as an internal standard for determining the ^{19}F NMR yield. No product was detected.

Experiments with radical scavengers:

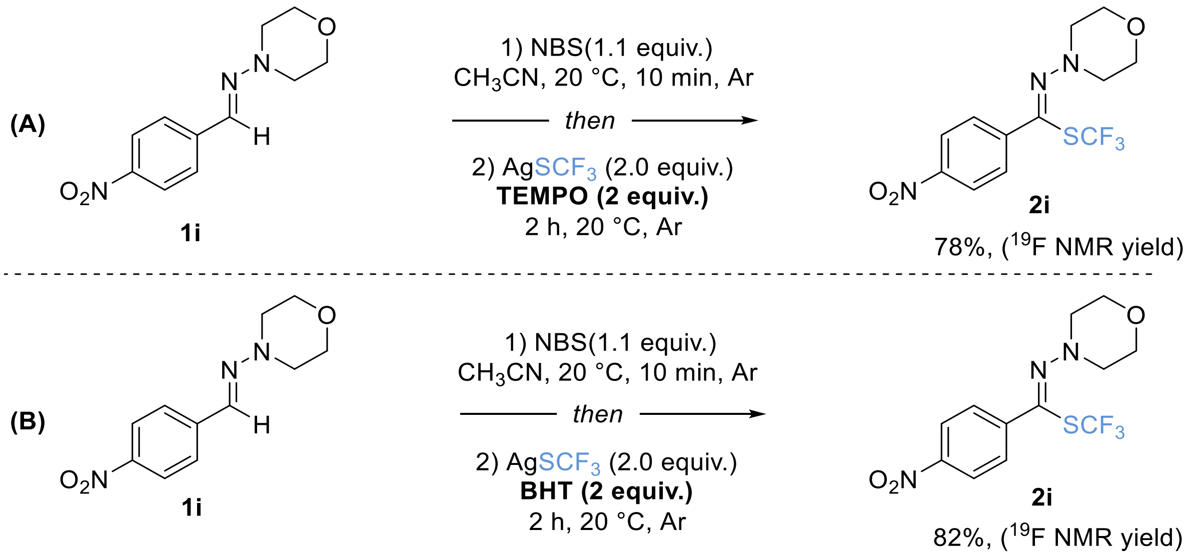

(A) An oven-dried 10 mL reaction tube equipped with a stirring bar was charged with the hydrazone derivative (0.3 mmol, 1.0 equiv) and CH_3CN (0.7 mL). The mixture was stirred until solubilization of the reagent. Then, recrystallized NBS (58.7 mg, 0.33 mmol, 1.1 equiv) was added, and the reaction mixture was stirred for 5–10 minutes. After which, $AgSCF_3$ (125.0 mg, 0.6 mmol, 2.0 equiv) was added alongside TEMPO (93.8 mg, 0.6 mmol, 2 equiv). The reaction mixture was stirred for another 2 hours at room temperature. α,α,α-Trifluoroacetophenone (42 µL, 0.3 mmol, 1.0 equiv) was added as an internal standard and ^{19}F NMR yield was measured in $CDCl_3$. The product **2i** was observed in 78% yield.

(B) An oven-dried 10 mL reaction tube equipped with a stirring bar was charged with the hydrazone derivative (0.3 mmol, 1.0 equiv) and CH_3CN (0.7 mL). The mixture was stirred until solubilization of the reagent. Then, recrystallized NBS (58.7 mg, 0.33 mmol, 1.1 equiv) was added, and the reaction mixture was stirred for 5–10 minutes. After which, $AgSCF_3$ (125.0 mg,

0.6 mmol, 2.0 equiv) was added alongside BHT (125.9 µL, 0.6 mmol, 2 equiv). The reaction mixture was stirred for another 2 hours at room temperature. α,α,α-Trifluoroacetophenone (42 µL, 0.3 mmol, 1.0 equiv) was added as an internal standard and ^{19}F NMR yield was measured in $CDCl_3$. The product **2i** was observed in 82% yield.

The presence of a radical scavenger does not appear to inhibit the reaction.

10. Post-functionalization reaction

Suzuki cross-coupling reaction on the compound 1g

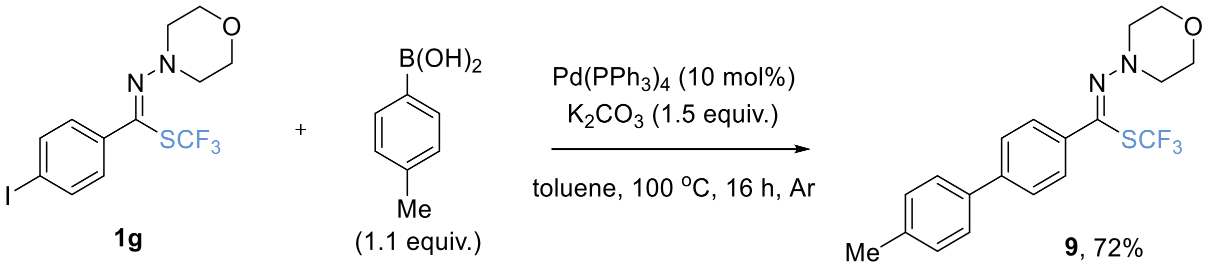

This procedure was adapted from Besset et al:[8] an oven-dried tube was loaded with **2g** (50 mg, 0.1 mmol, 1 equiv), $Pd(PPh_3)_4$ (11.5 mg, 0.01 mmol, 10 mol %), *p*-tolylboronic acid (15 mg, 0.11 mmol, 1.1 equiv), and K_2CO_3 (20.7 mg, 0.15 mmol, 1.5 equiv). Freshly distilled toluene (2 mL) was injected and the reaction mixture was stirred at 100 °C for 16 h under Ar. Then, the solvent was removed under reduced pressure. The reaction mixture was purified by flash column chromatography on silica gel and product **9** was obtained (27.5 mg, 72%).

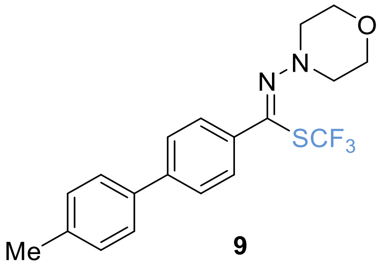

9

Trifluoromethyl (*Z*)-4'-methyl-*N*-morpholino-[1,1'-biphenyl]-4-carbimidothioate (9): The product was purified by silica gel flash column chromatography (height 12 cm, width 2 cm) eluting with *n*-pentane/ethyl acetate (98:02 to 96:04). The desired product was isolated as a yellow solid (27.5 mg, 72%). mp: 104 – 106 °C. R_f (*n*-pentane/ethyl acetate = 96:04): 0.42. **^{1}H NMR** (400.2 MHz, $CDCl_3$) δ 7.63 – 7.54 (m, 4H), 7.51 – 7.47 (m, 2H), 7.25 (d, *J* = 8.1 Hz, 2H), 3.89 – 3.83 (m, 4H), 3.00 – 2.95 (m, 4H), 2.39 (s, 3H). **^{19}F NMR** (376.6 MHz, $CDCl_3$) δ –36.7 (s, 3F). **^{13}C NMR** (100.6 MHz, $CDCl_3$) δ 158.2, 143.2, 137.8, 137.2, 132.9, 132.1 (q, *J* = 309.8 Hz), 129.6, 128.9, 127.0, 126.6, 66.0, 54.3, 21.1. **IR** (neat, cm^{-1}) ν: 2923, 2859, 1583, 1459, 1263, 1138, 1103, 974, 810, 754, 641, 453, 392. **HRMS** (ESI^+) calcd for $C_{19}H_{20}ON_2F_3S$ *m/z* 381.1243 $[M+H]^+$, Found 381.1243 (Δ = -0.10 ppm).

11. NMR Spectra

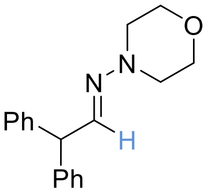

1s

^{1}H NMR (400.2 MHz, $CDCl_3$)

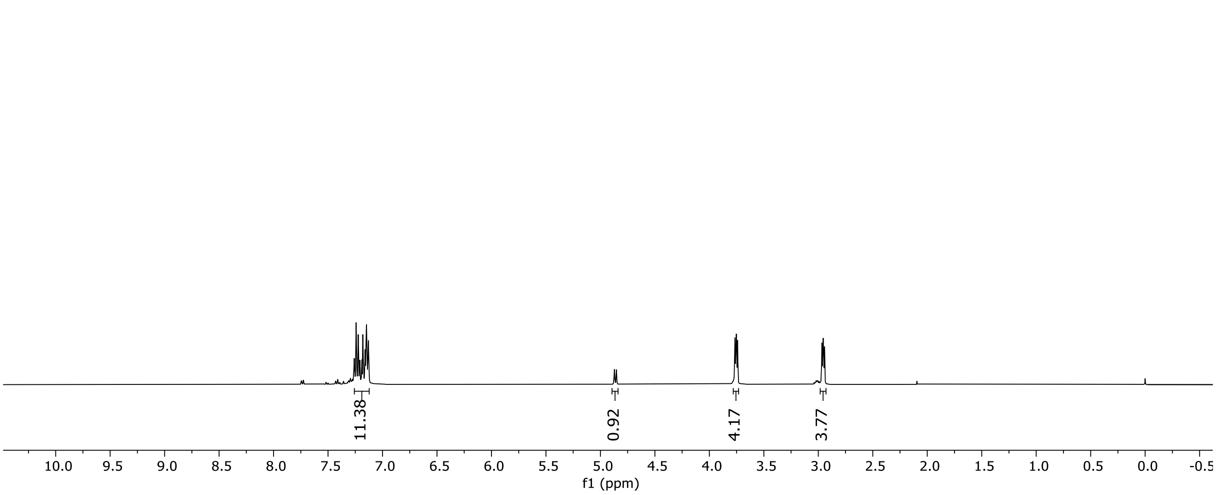

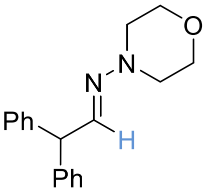

1s

^{13}C NMR (100.6 MHz, $CDCl_3$)

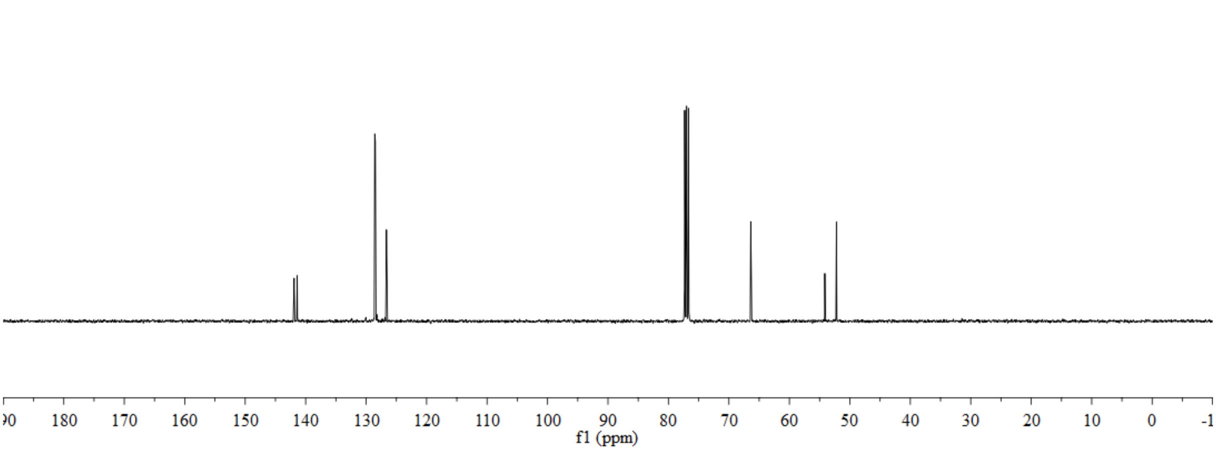

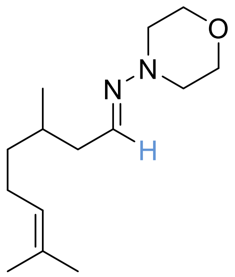

1u

^{1}H NMR (400.2 MHz, $CDCl_3$)

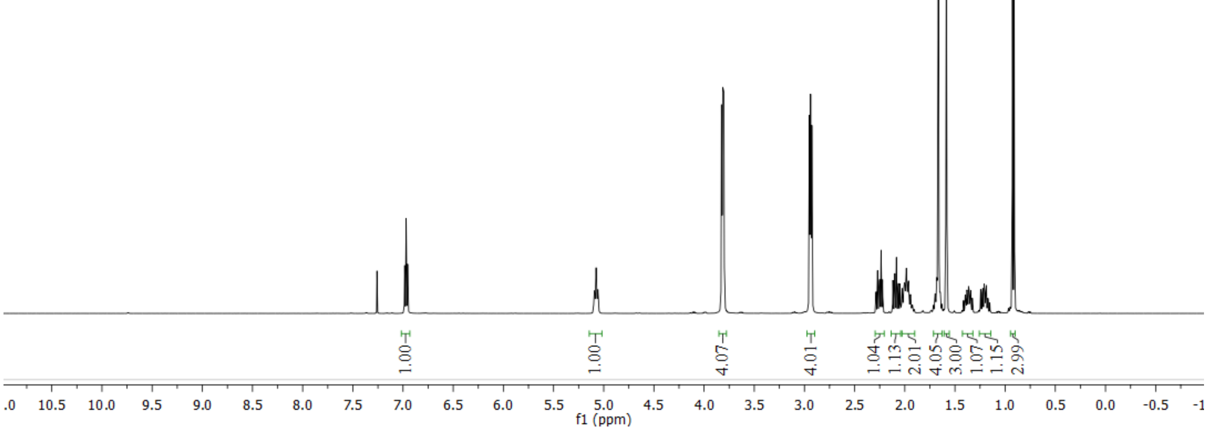

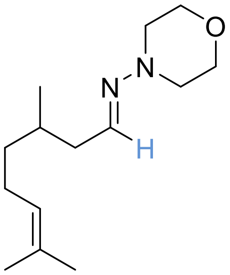

1u

^{13}C NMR (100.6 MHz, $CDCl_3$)

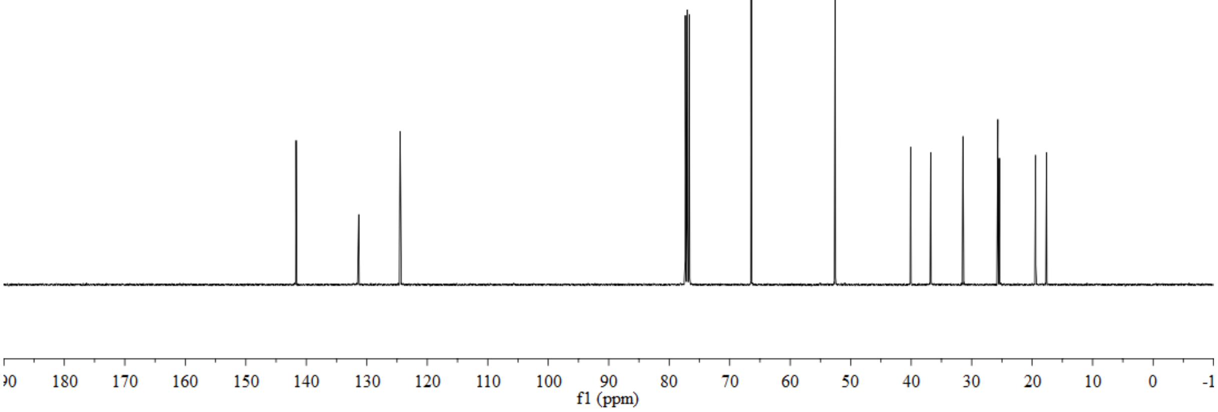

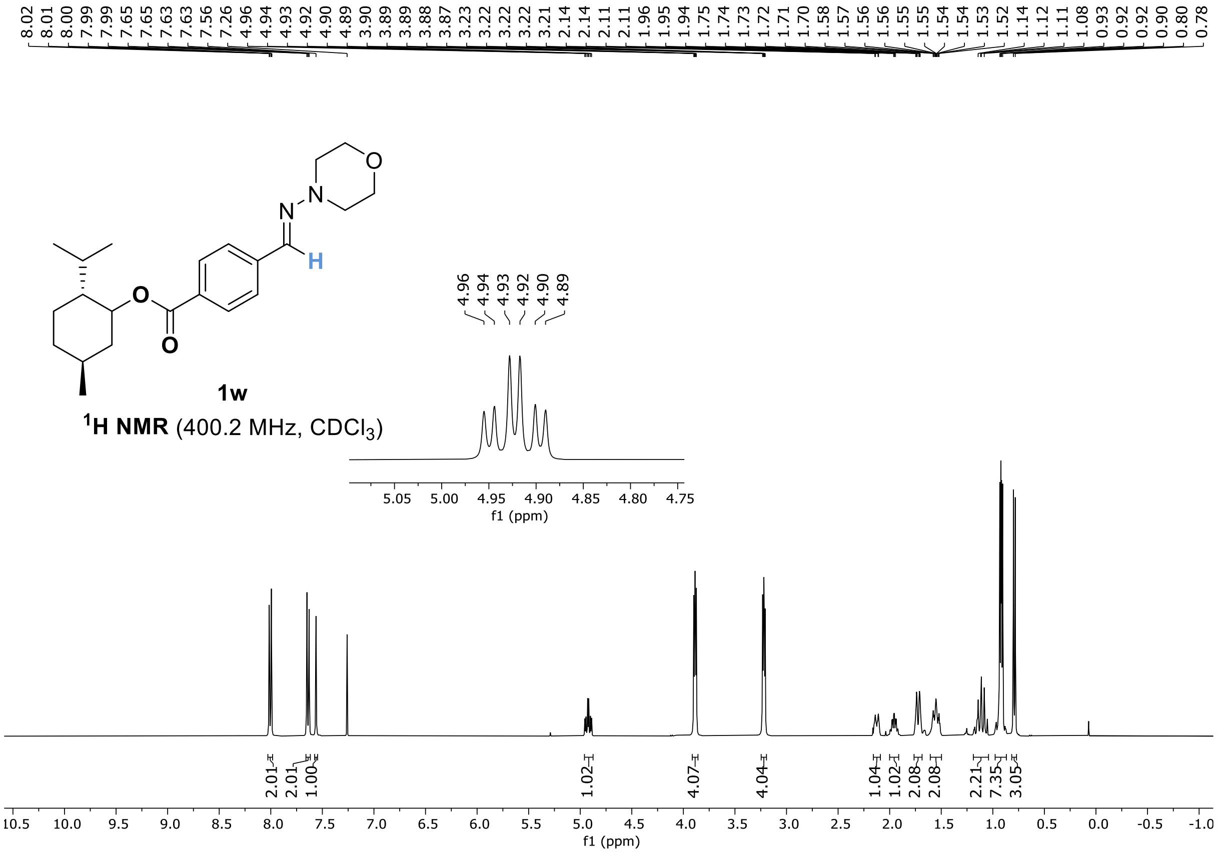

1w

^{1}H NMR (400.2 MHz, $CDCl_3$)

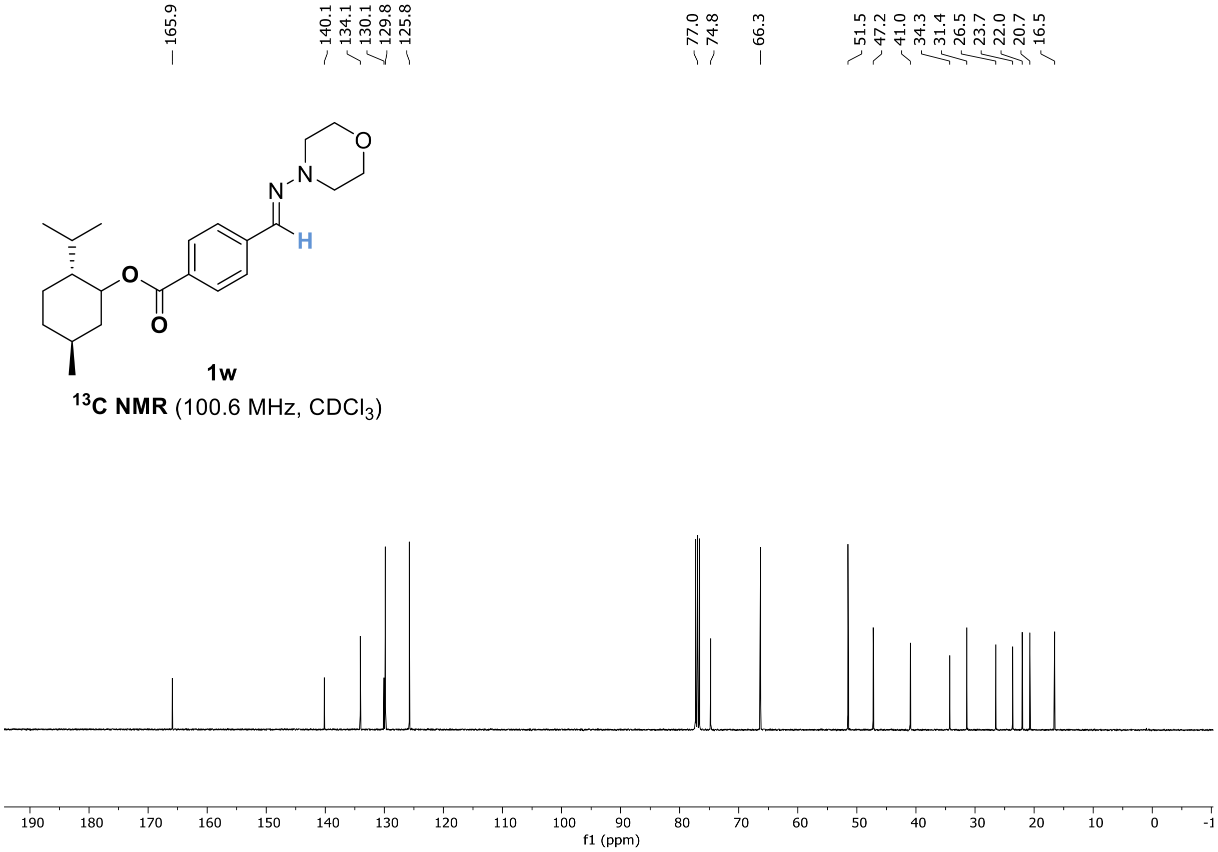

1w

^{13}C NMR (100.6 MHz, $CDCl_3$)

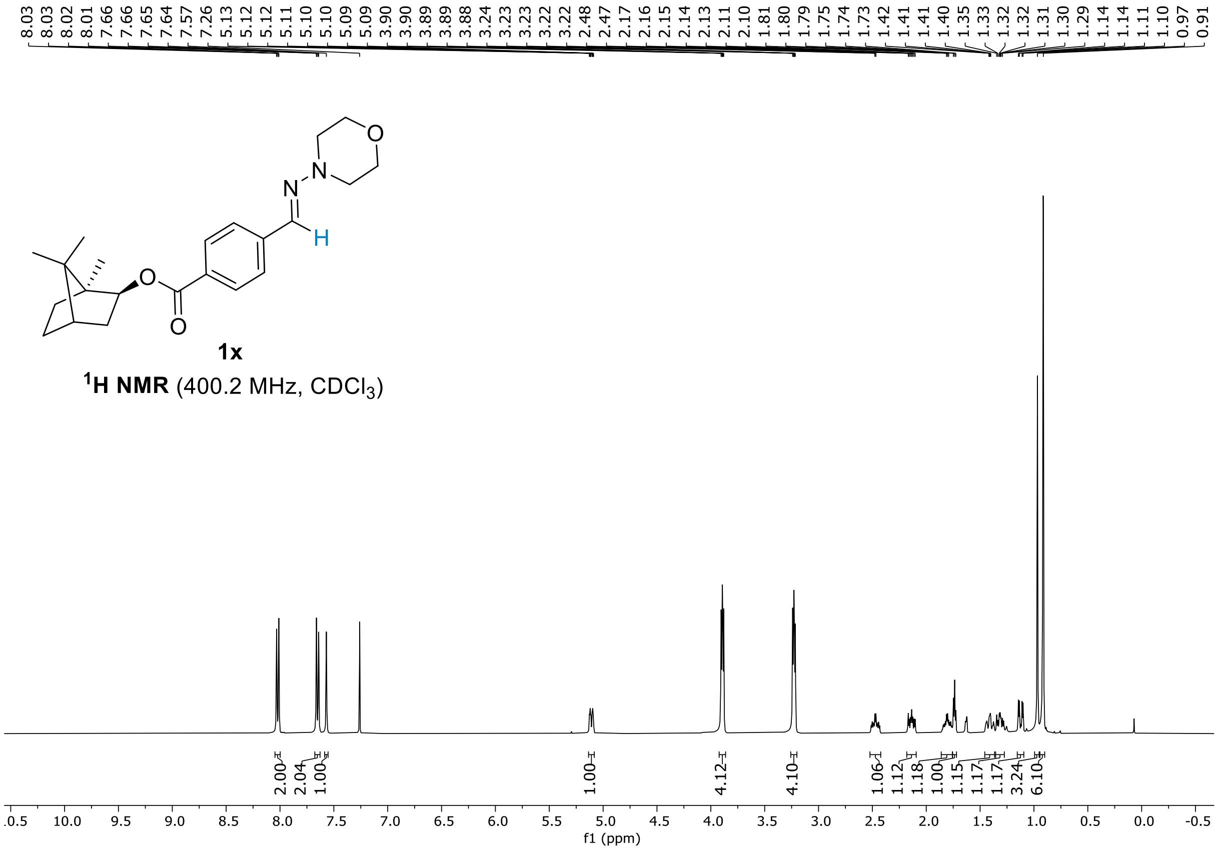
1x
1H NMR (400.2 MHz, CDCl3)
f1 (ppm)

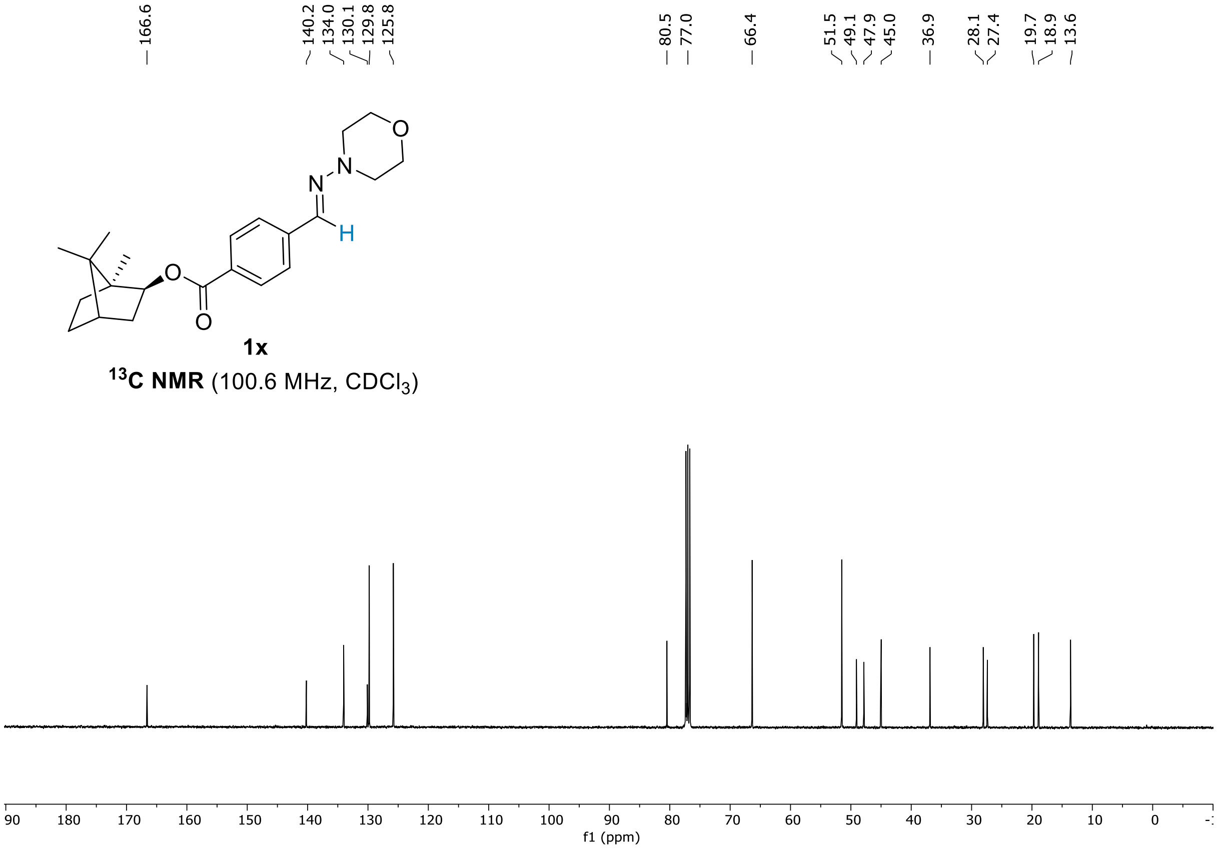
1x
13C NMR (100.6 MHz, CDCl3)
f1 (ppm)

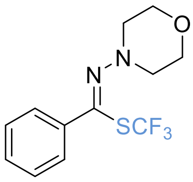

2a

¹H NMR (400.2 MHz, $CDCl_3$)

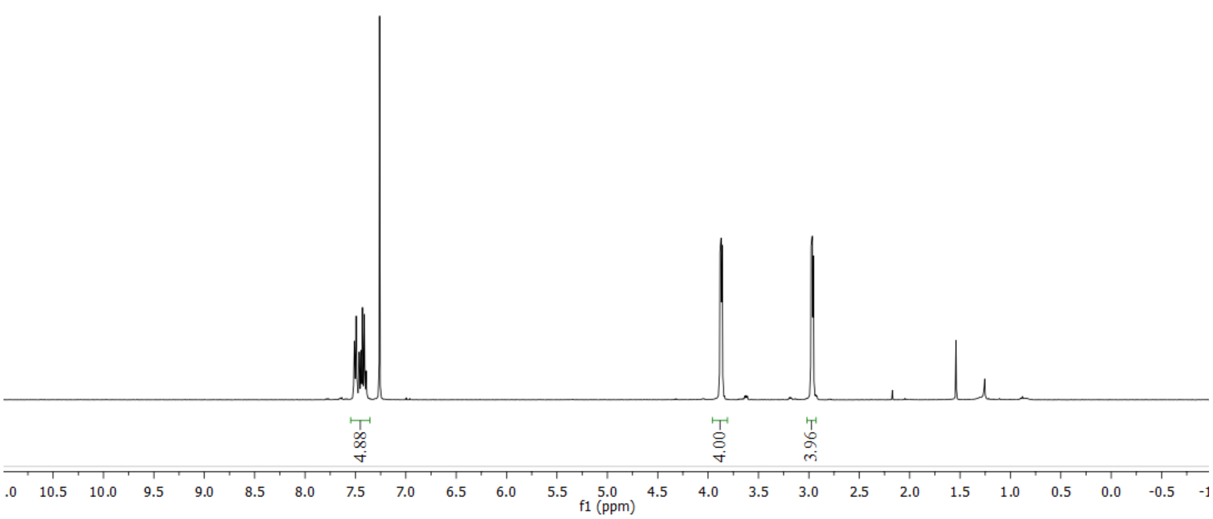

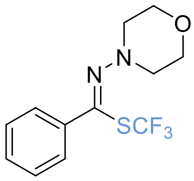

2a

¹⁹F NMR (376.6 MHz, $CDCl_3$)

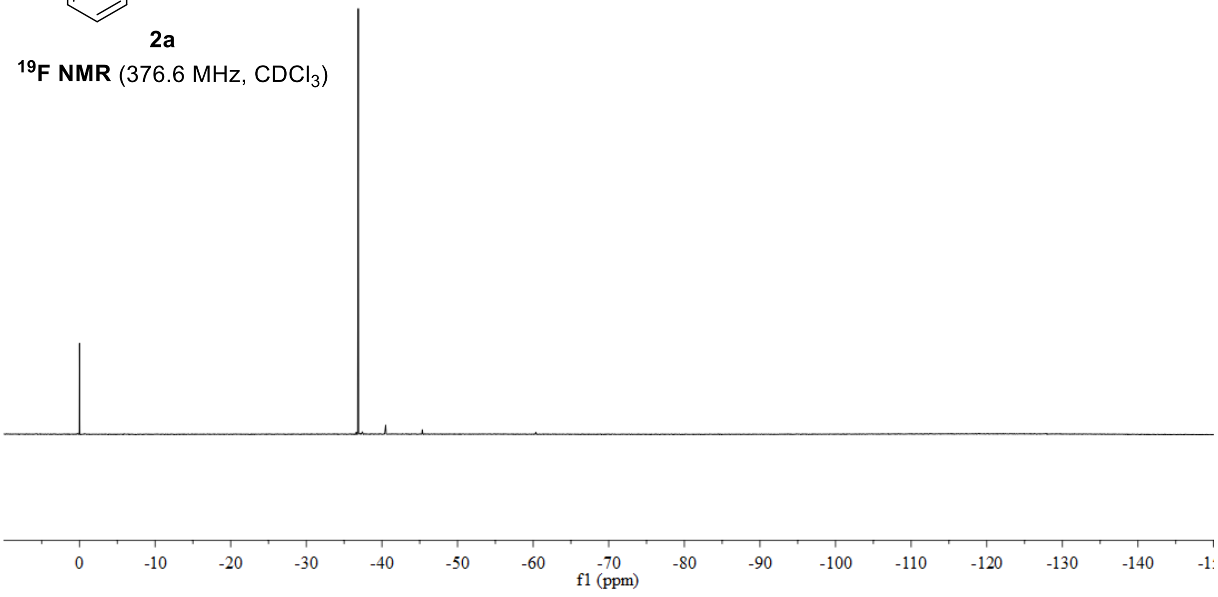

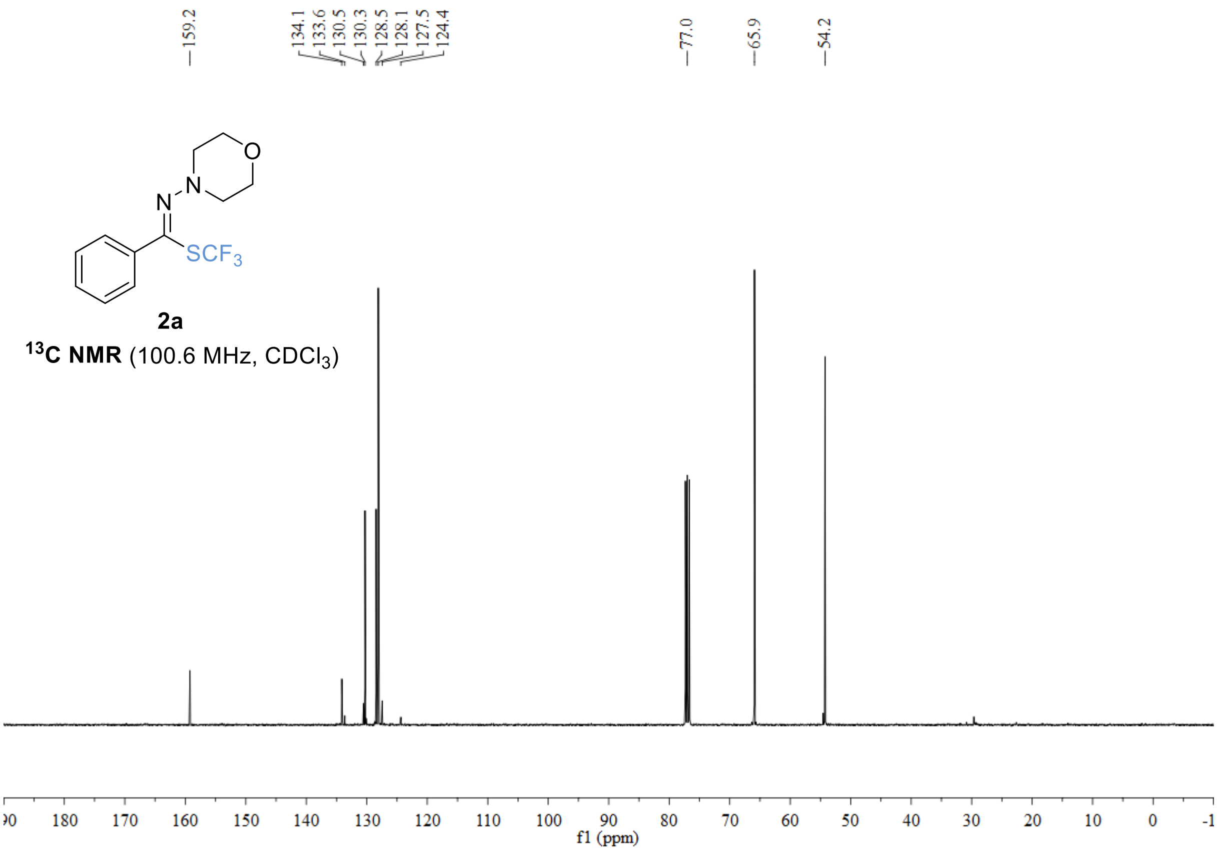
159.2
134.1
133.6
130.5
130.3
128.5
128.1
127.5
124.4
77.0
65.9
54.2
SCF3
N
N
O
2a
13C NMR (100.6 MHz, CDCl3)
f1 (ppm)

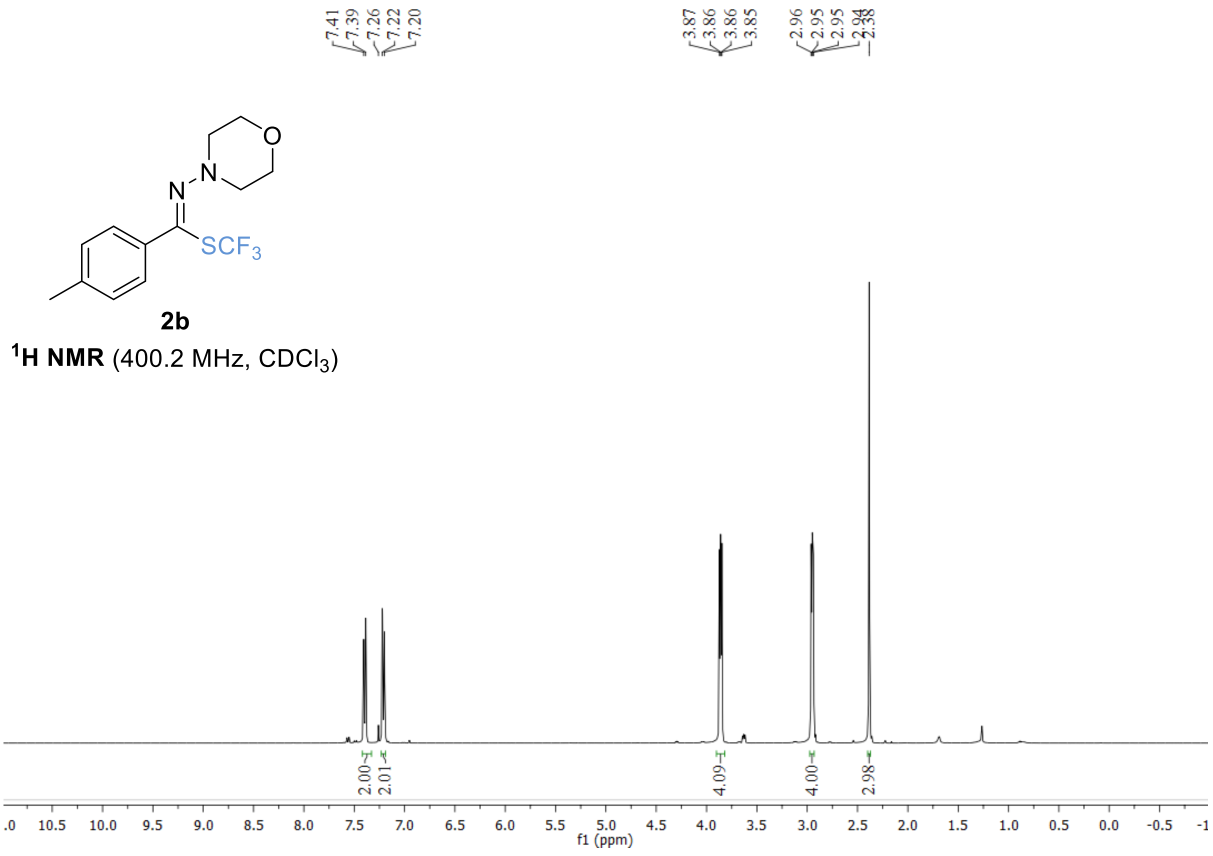
7.41
7.39
7.26
7.22
7.20
3.87
3.86
3.86
3.85
2.96
2.95
2.95
2.94
2.38
SCF3
N
N
O
2b
1H NMR (400.2 MHz, CDCl3)
2.00
2.01
4.09
4.00
2.98
f1 (ppm)

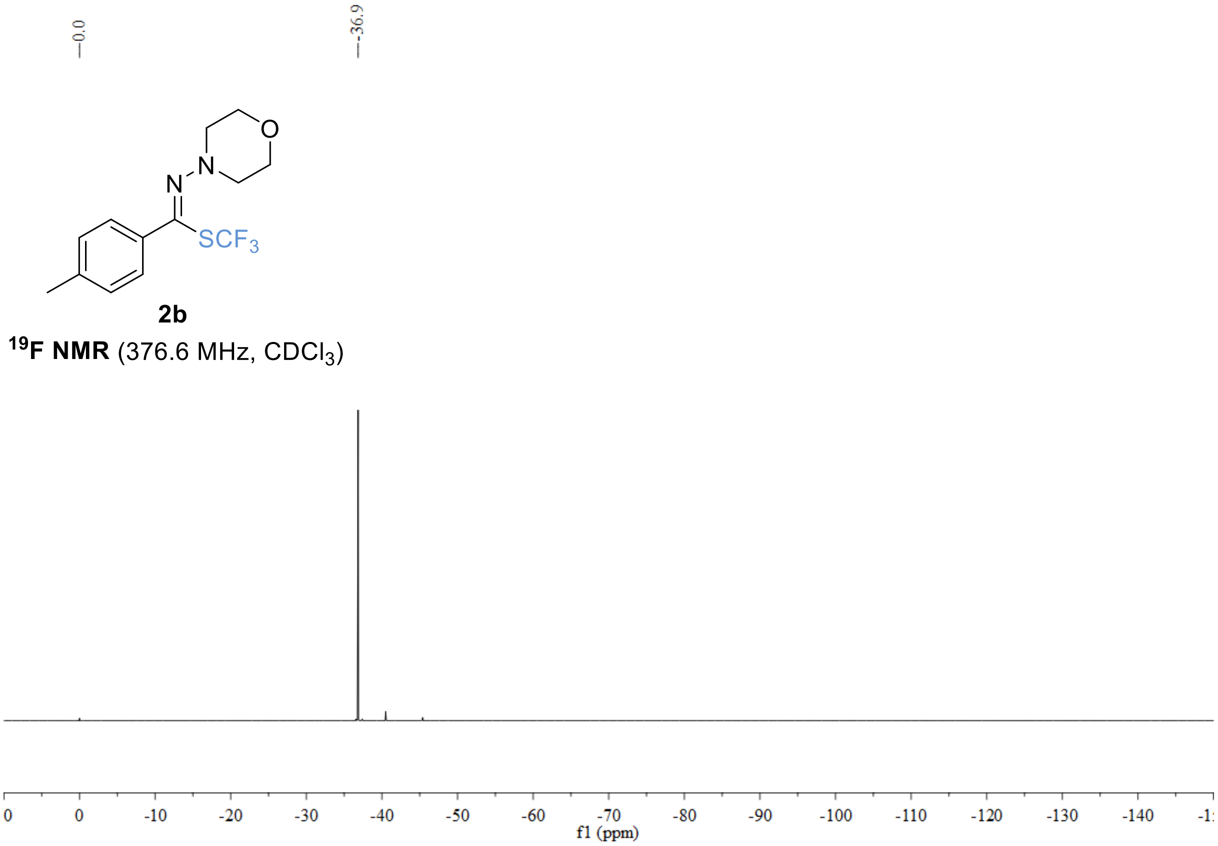

2b

^{19}F NMR (376.6 MHz, $CDCl_3$)

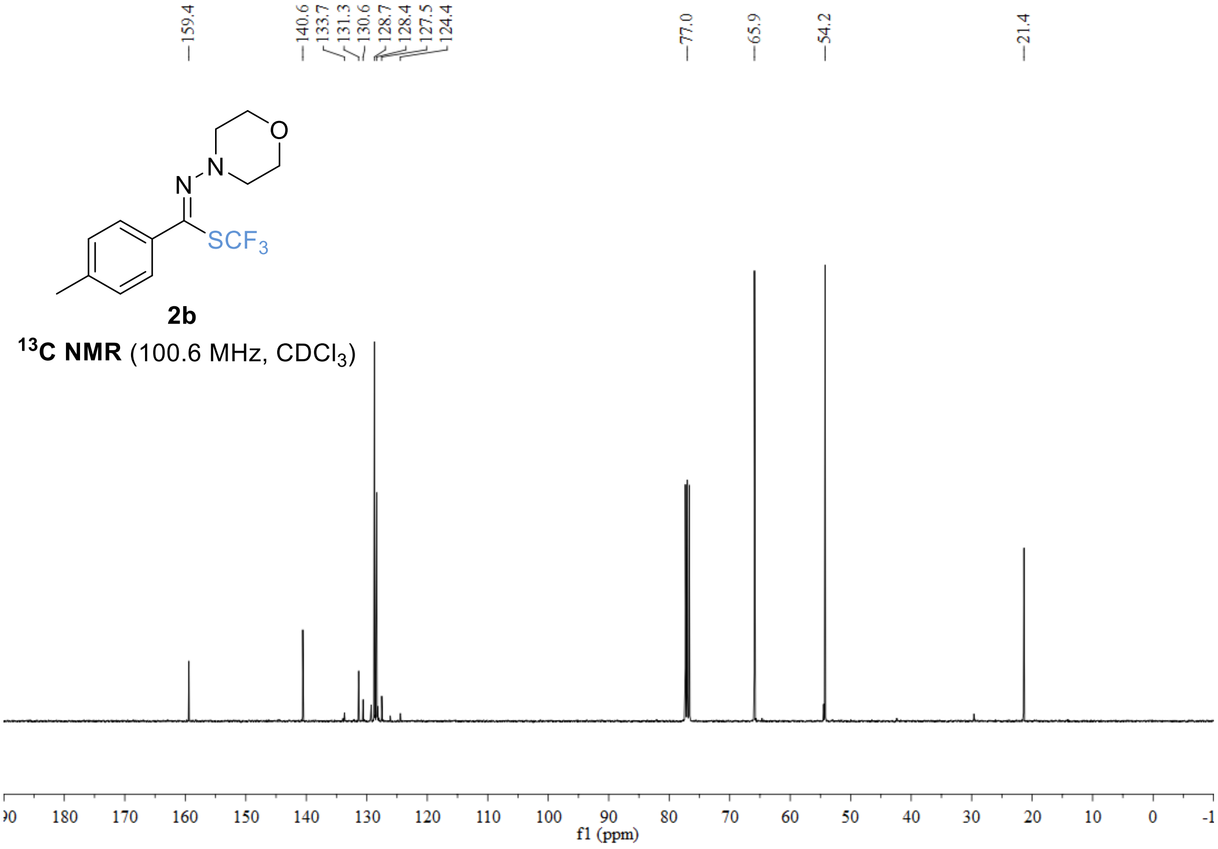

2b

^{13}C NMR (100.6 MHz, $CDCl_3$)

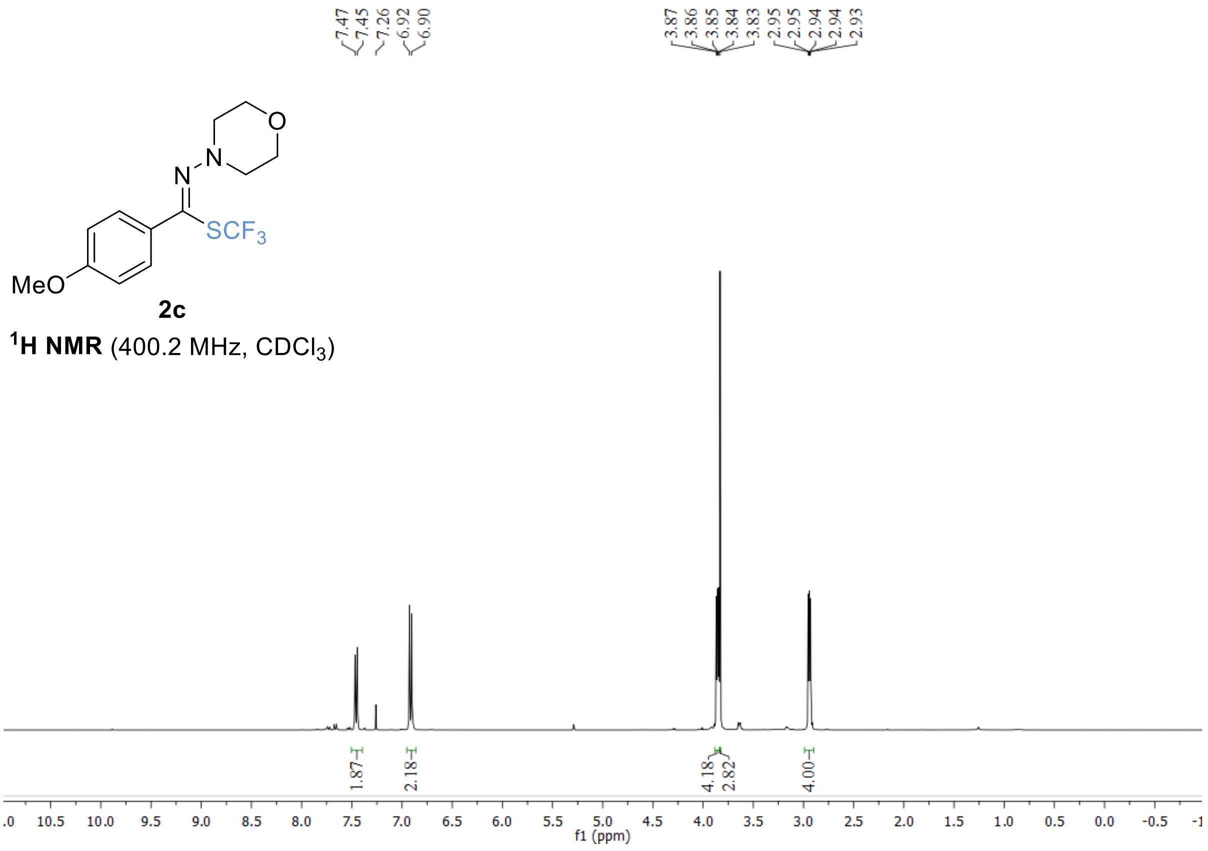
7.47
7.45
7.26
6.92
6.90
3.87
3.86
3.85
3.84
3.83
2.95
2.95
2.94
2.94
2.93
MeO
SCF3
2c
1H NMR (400.2 MHz, CDCl3)
1.87
2.18
4.18
2.82
4.00
f1 (ppm)

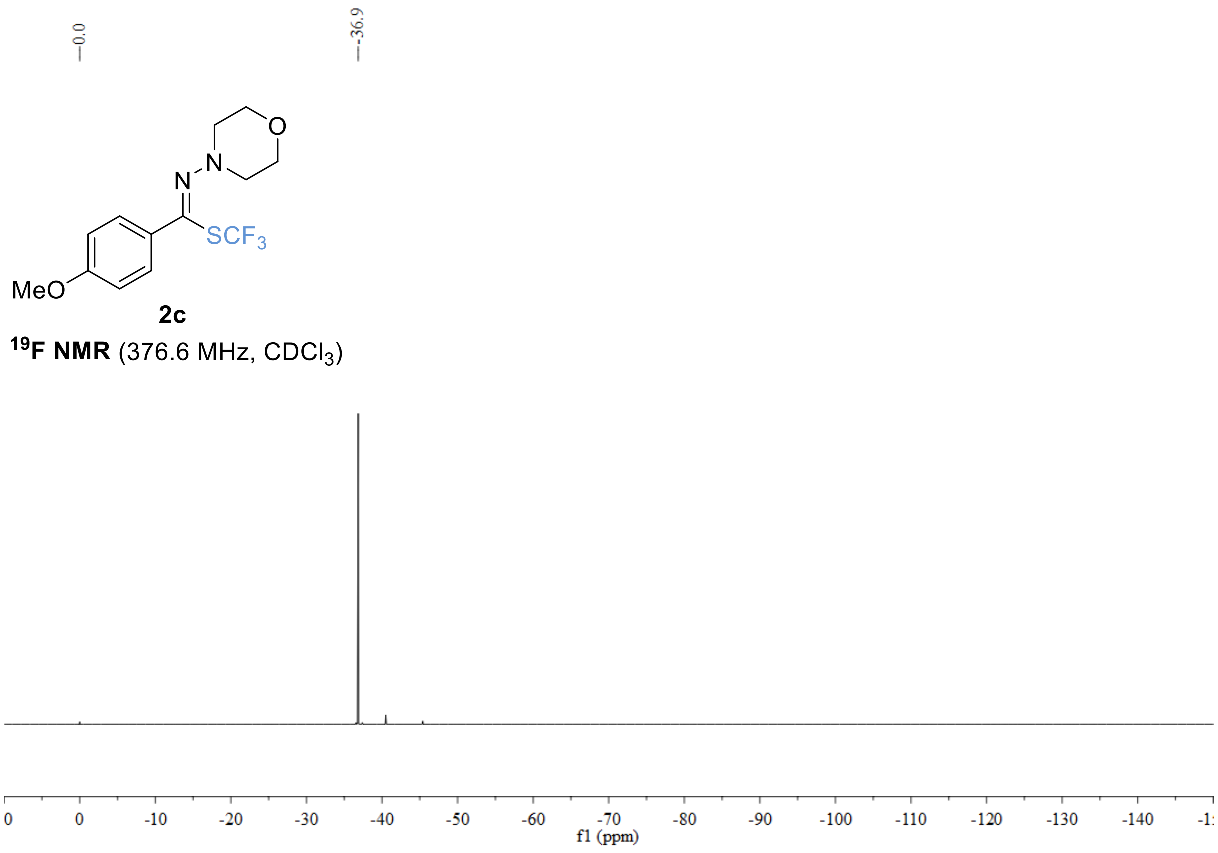
-0.0
-36.9
MeO
SCF3
2c
19F NMR (376.6 MHz, CDCl3)
f1 (ppm)

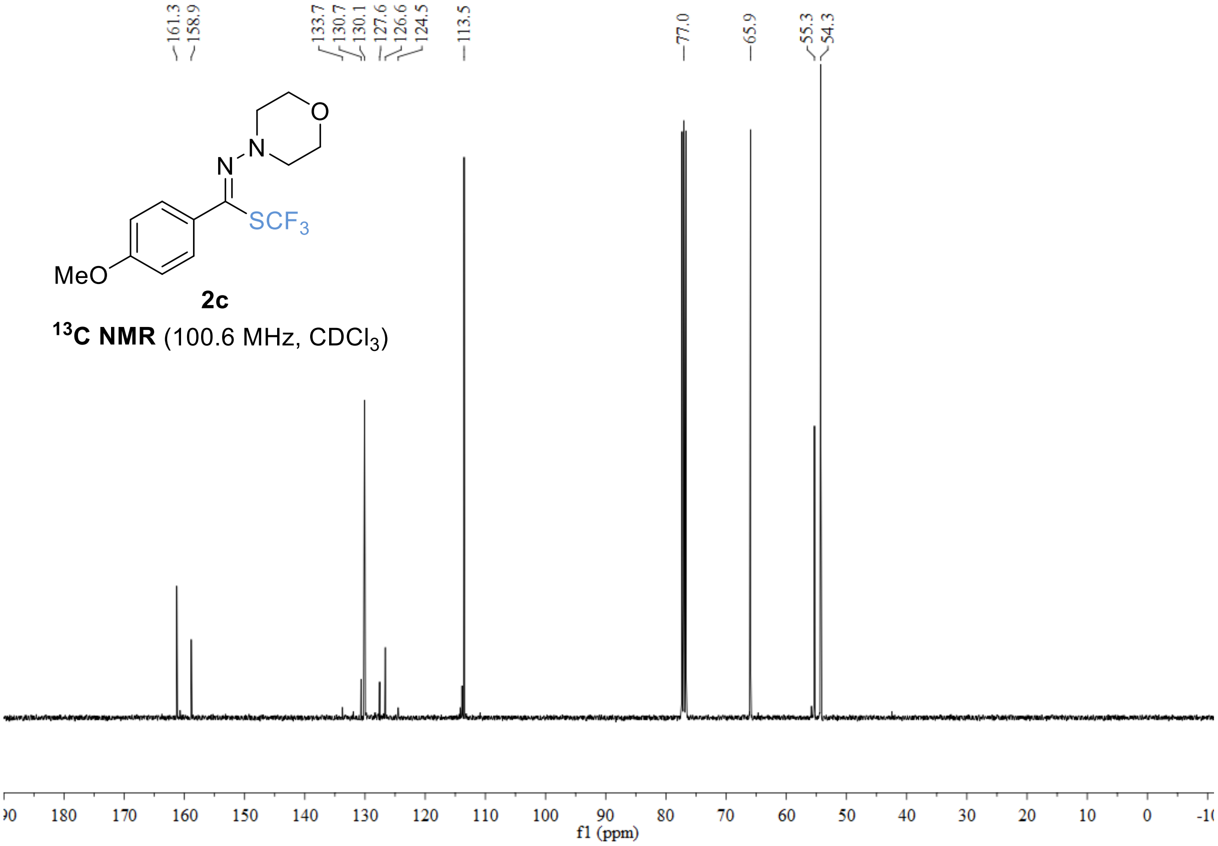

2c

^{13}C NMR (100.6 MHz, $CDCl_3$)

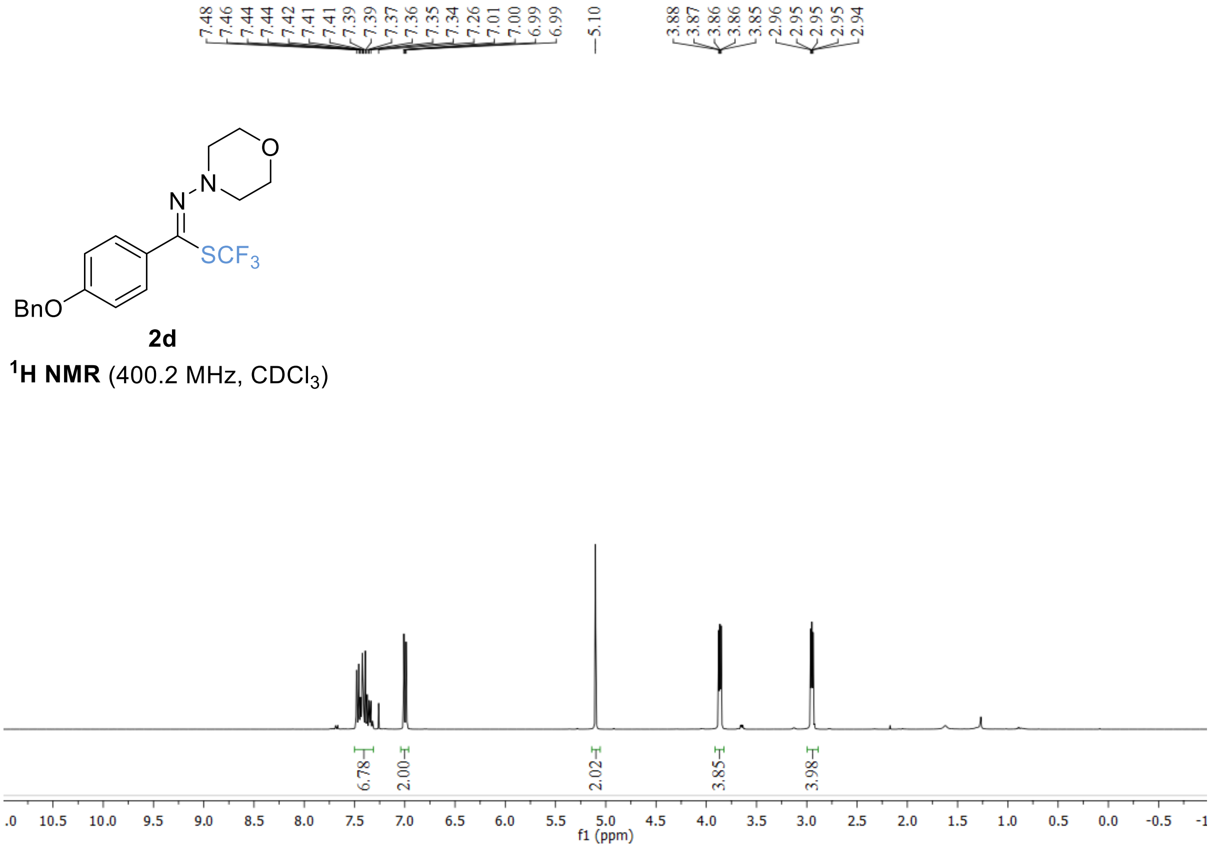

2d

^{1}H NMR (400.2 MHz, $CDCl_3$)

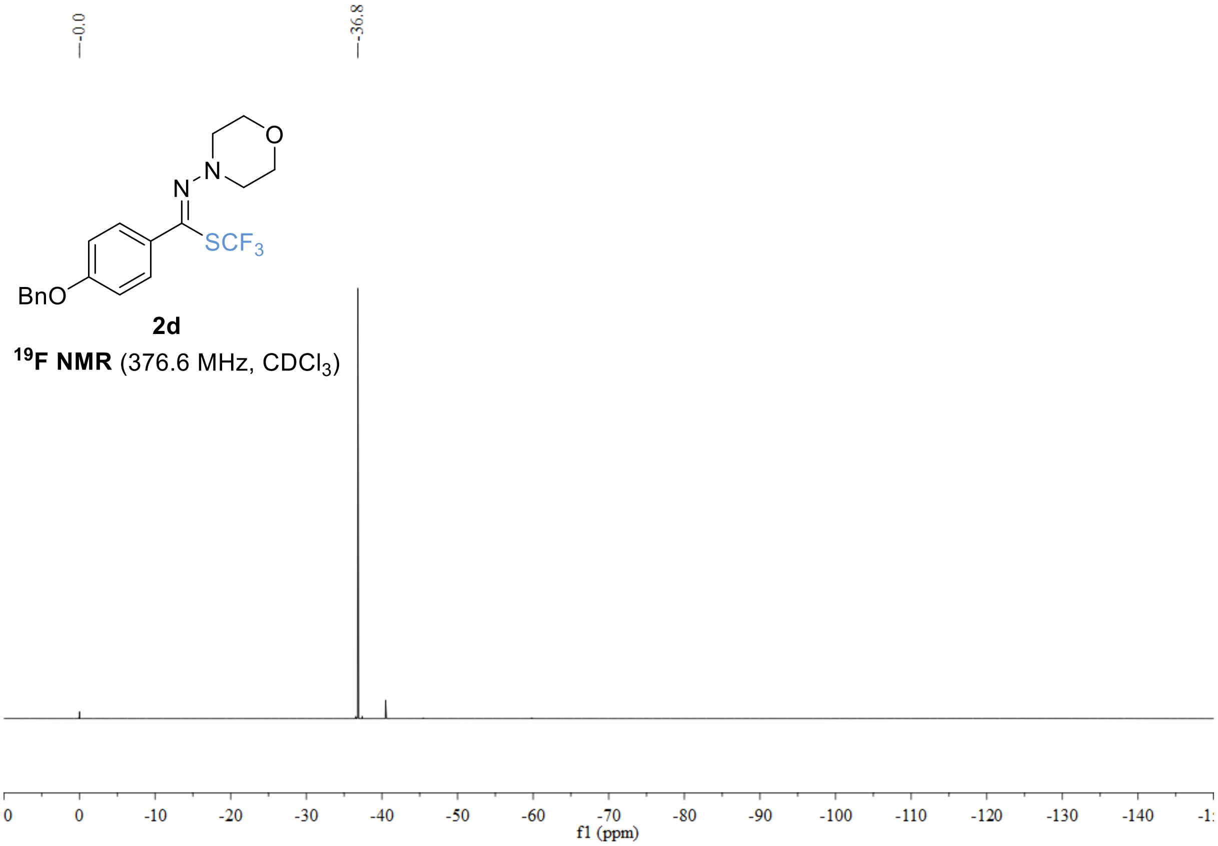
-0.0
-36.8
N
N
O
SCF3
BnO
2d
19F NMR (376.6 MHz, CDCl3)
0
0
-10
-20
-30
-40
-50
-60
-70
-80
-90
-100
-110
-120
-130
-140
f1 (ppm)

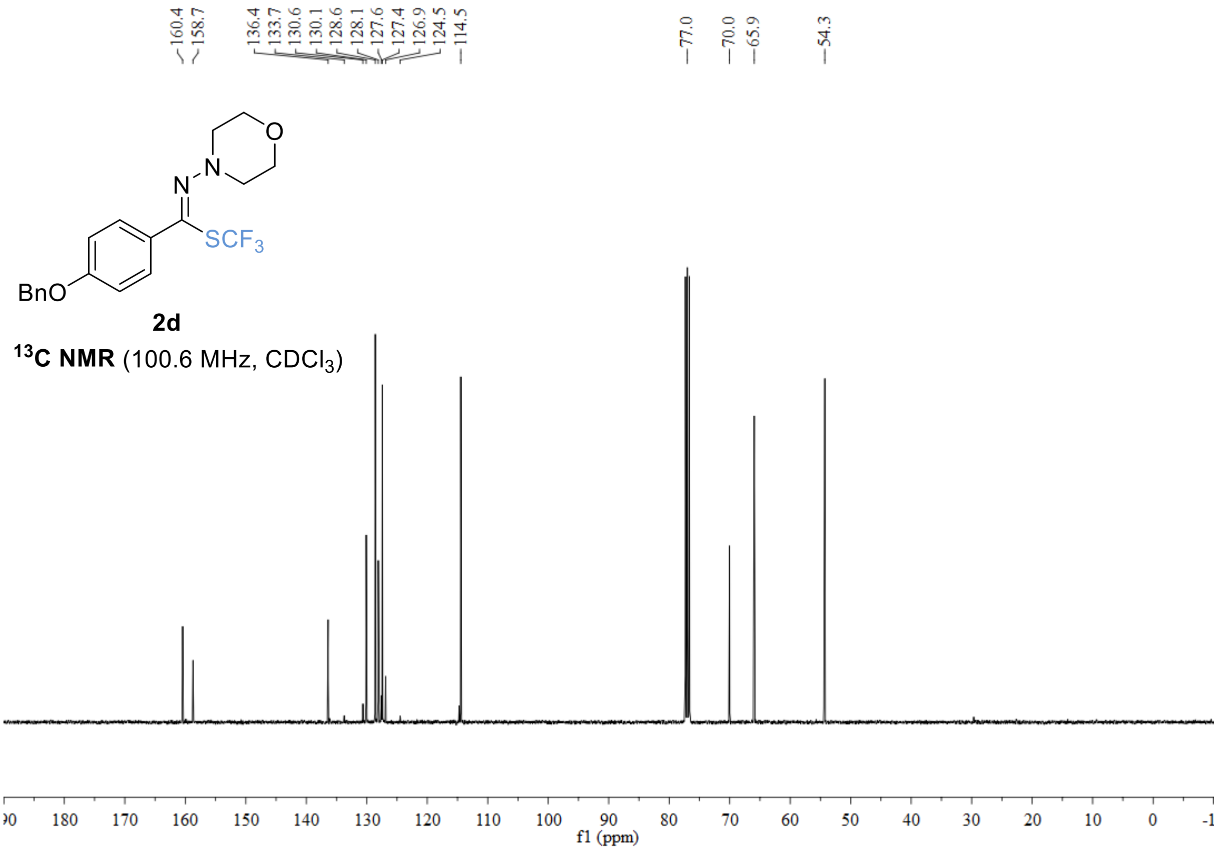
160.4
158.7
136.4
133.7
130.6
130.1
128.6
128.1
127.6
127.4
126.9
124.5
114.5
77.0
70.0
65.9
54.3
N
N
O
SCF3
BnO
2d
13C NMR (100.6 MHz, CDCl3)
180
170
160
150
140
130
120
110
100
90
80
70
60
50
40
30
20
10
0
f1 (ppm)

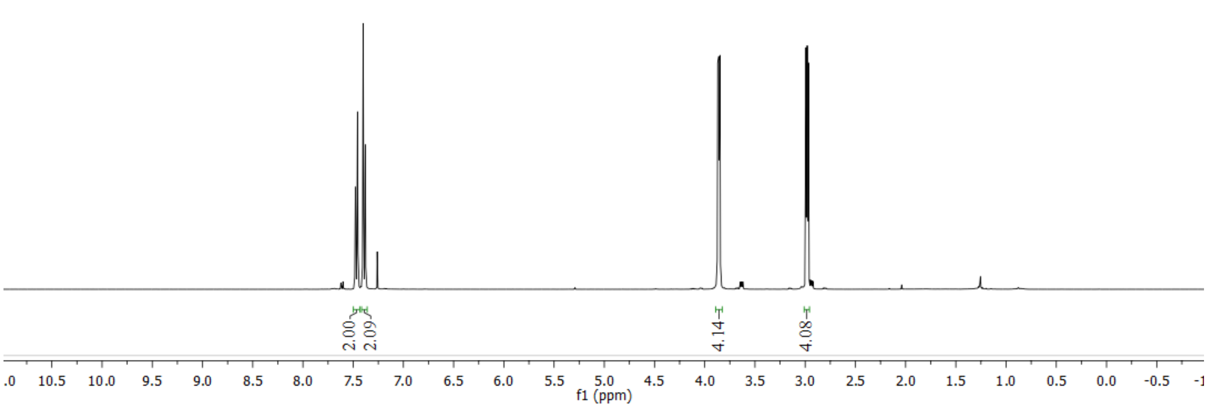

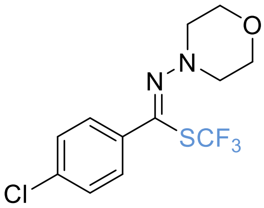

2e

^{1}H NMR (400.2 MHz, $CDCl_3$)

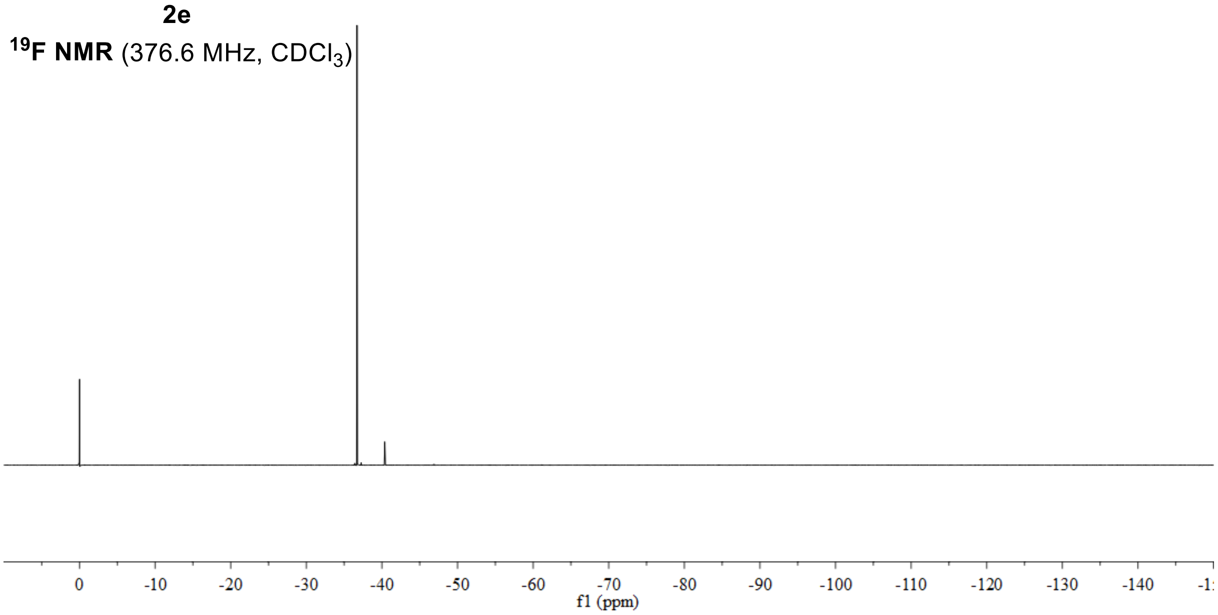

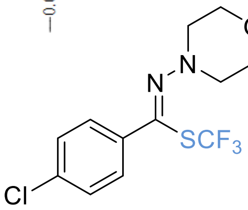

2e

^{19}F NMR (376.6 MHz, $CDCl_3$)

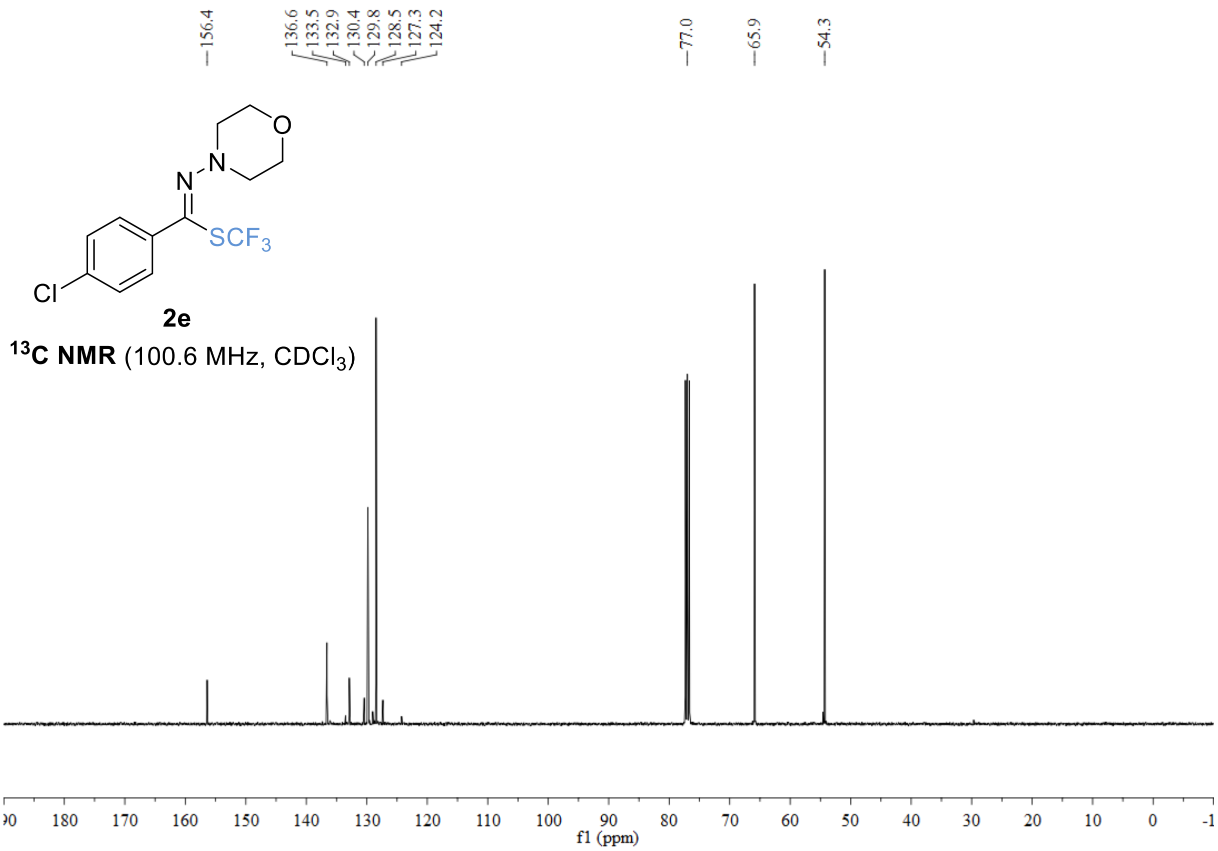

2e

^{13}C NMR (100.6 MHz, $CDCl_3$)

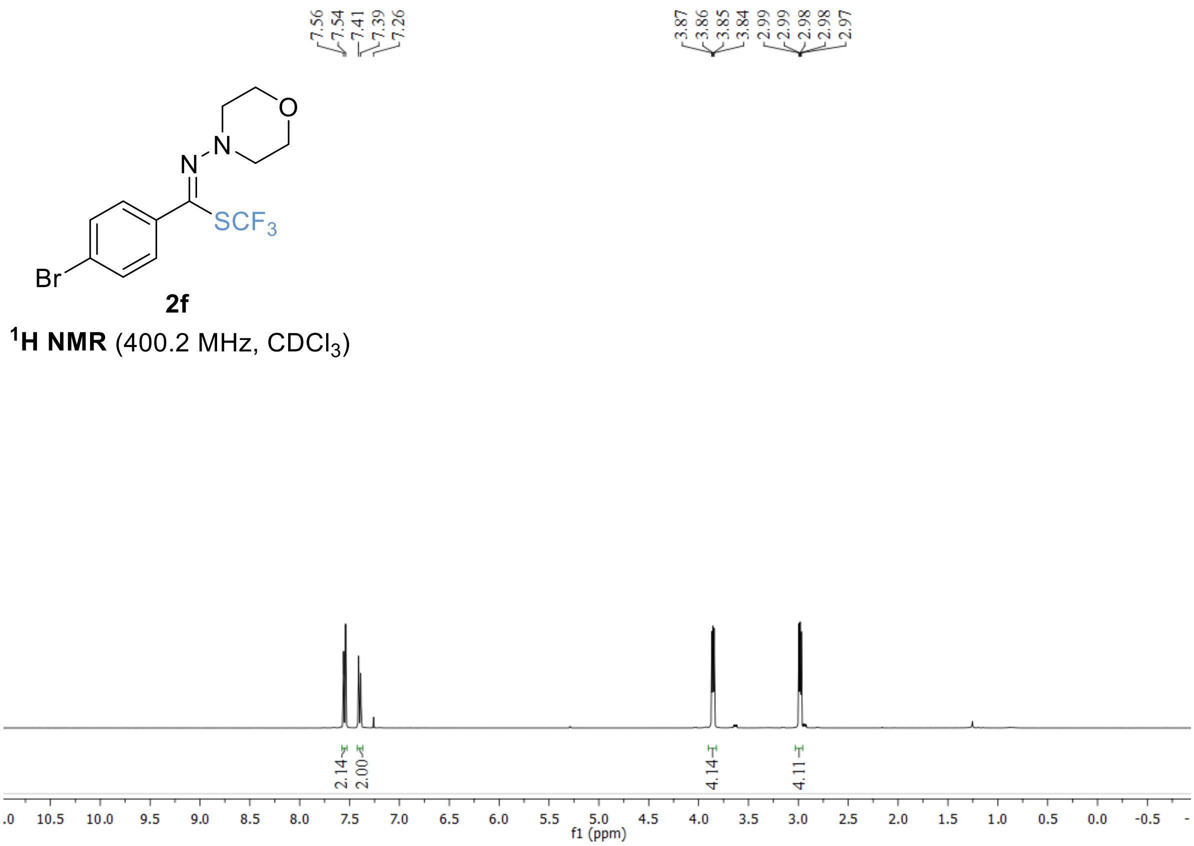

2f

^{1}H NMR (400.2 MHz, $CDCl_3$)

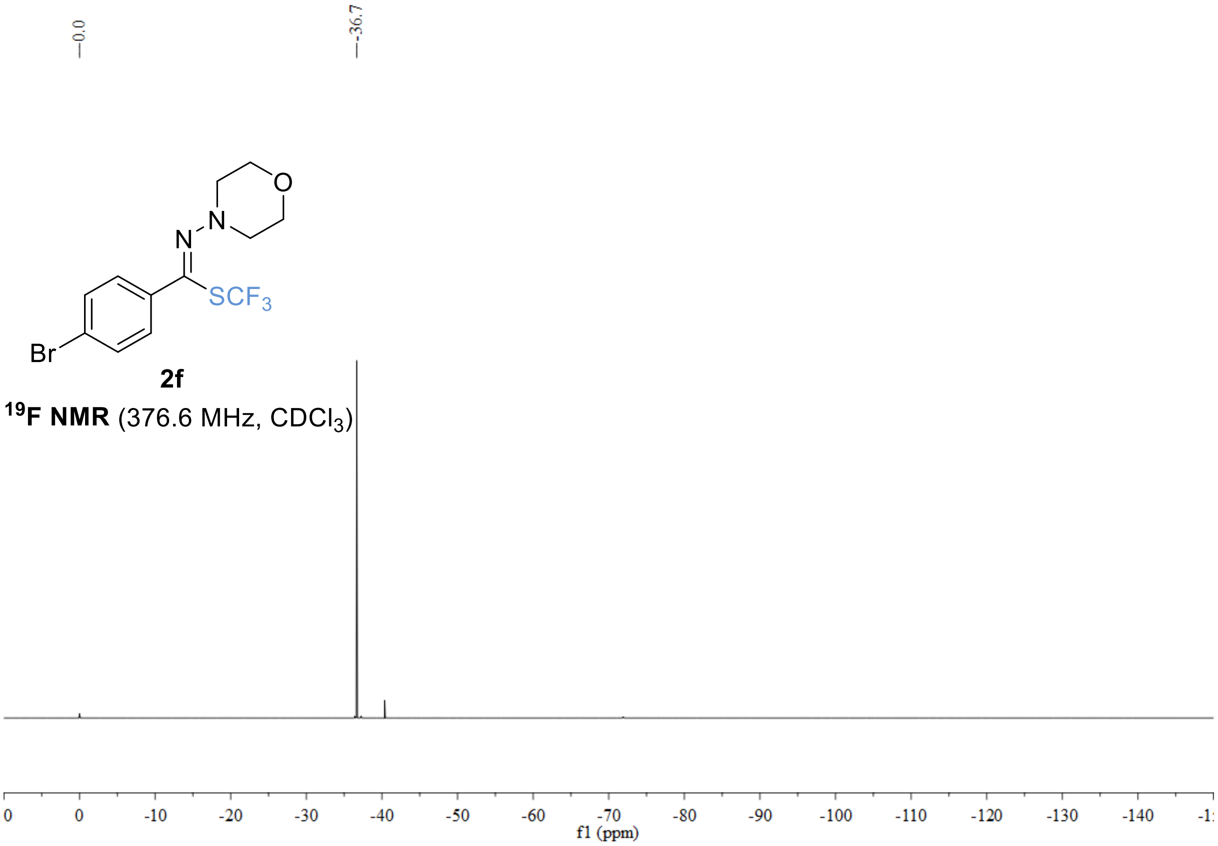

2f

^{19}F NMR (376.6 MHz, $CDCl_3$)

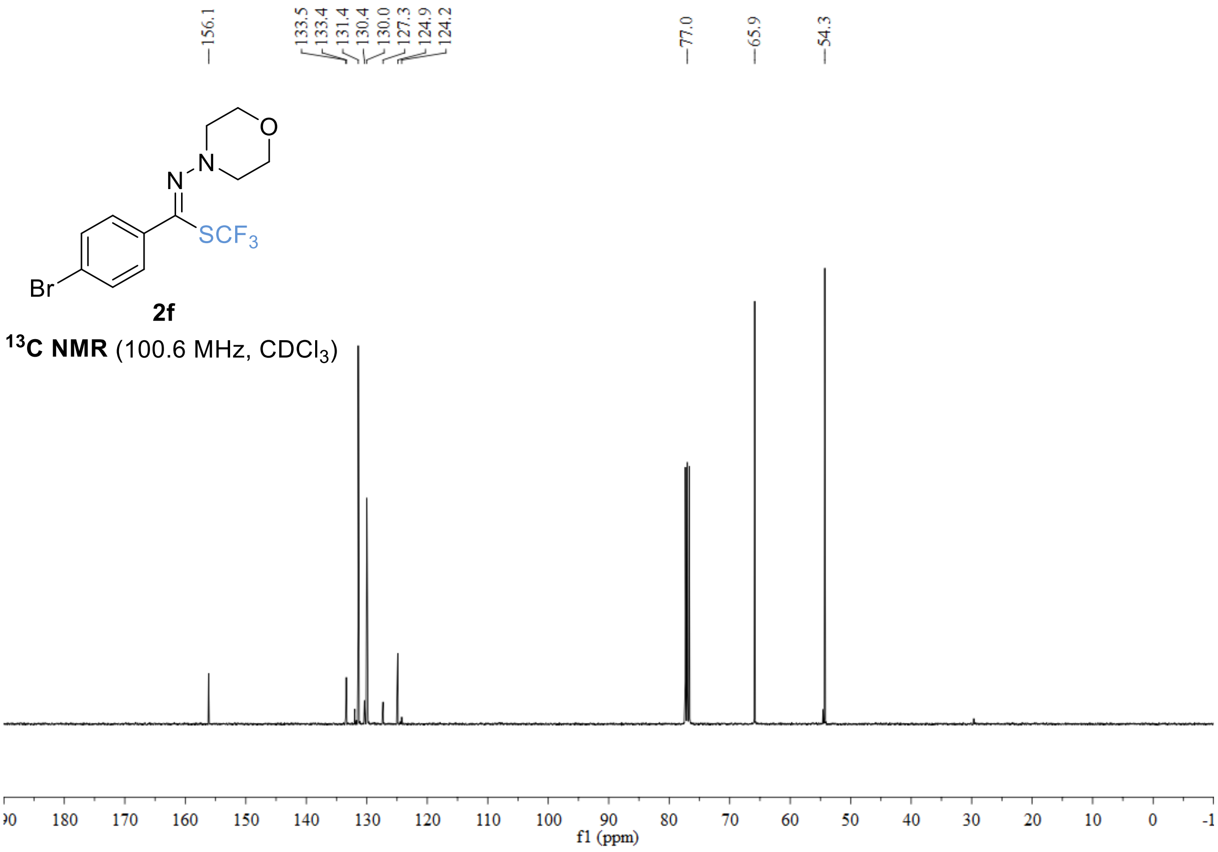

2f

^{13}C NMR (100.6 MHz, $CDCl_3$)

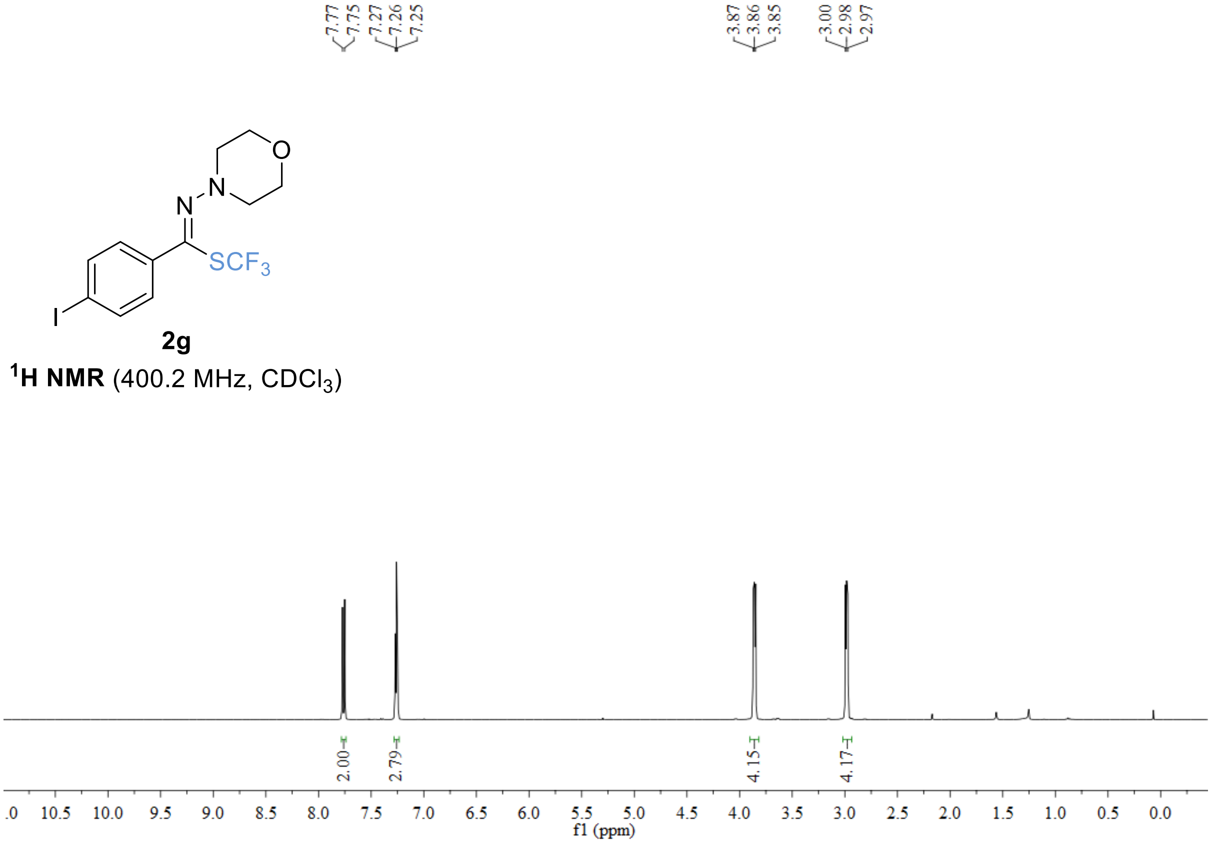

2g

^{1}H NMR (400.2 MHz, $CDCl_3$)

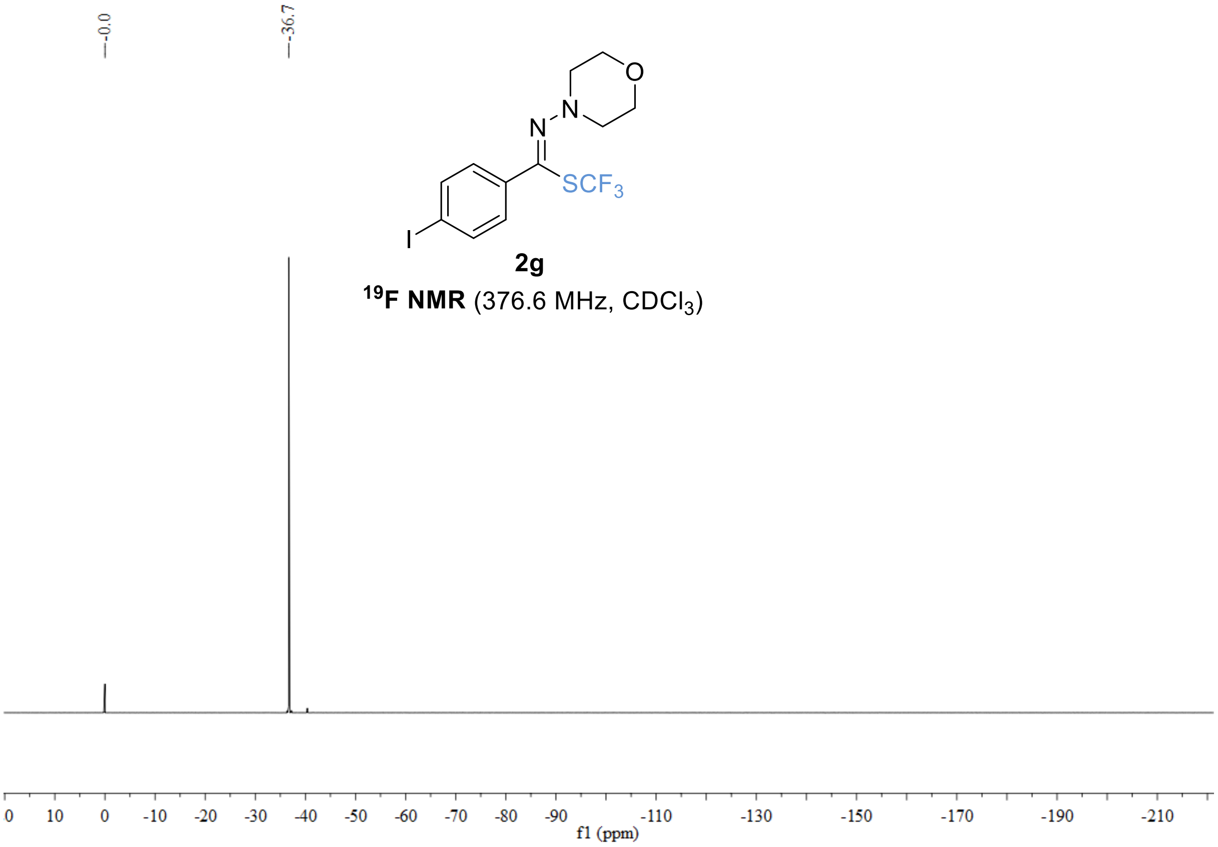

2g

^{19}F NMR (376.6 MHz, $CDCl_3$)

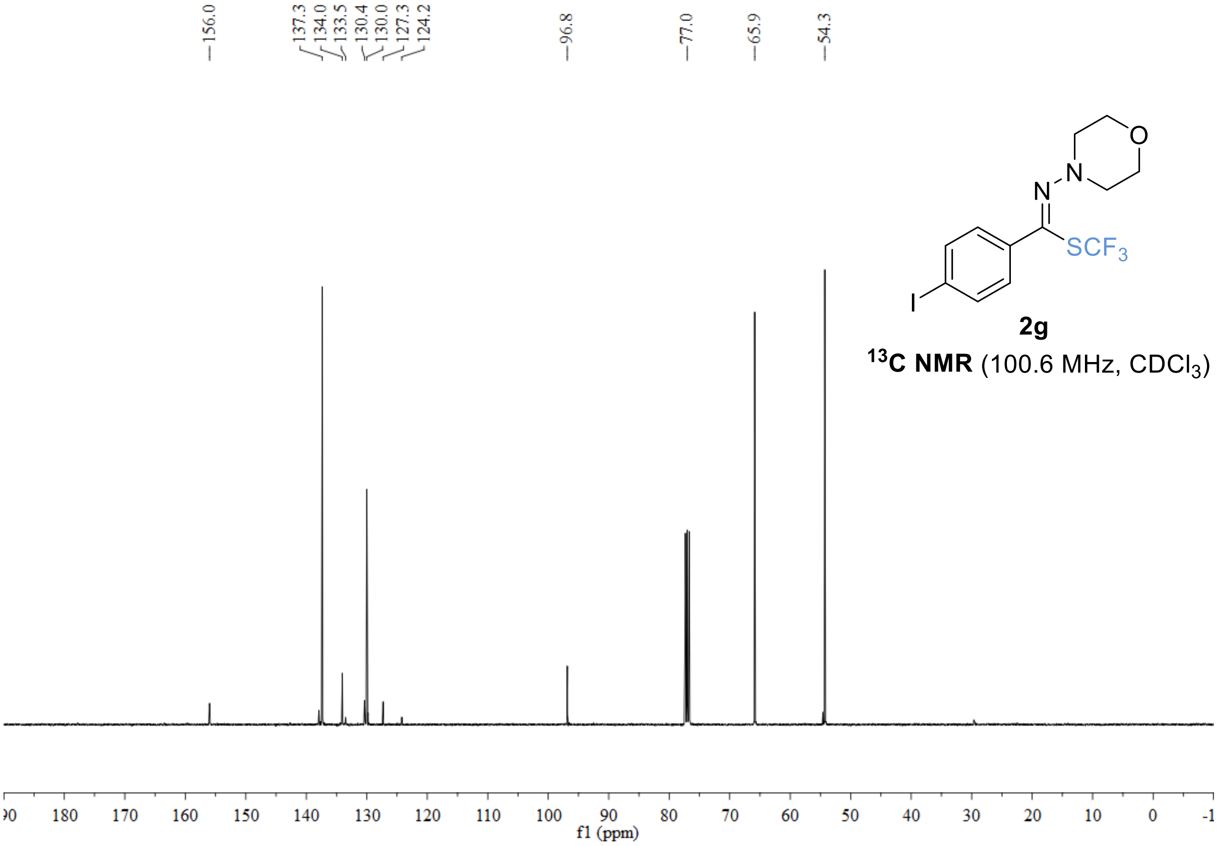

2g

^{13}C NMR (100.6 MHz, $CDCl_3$)

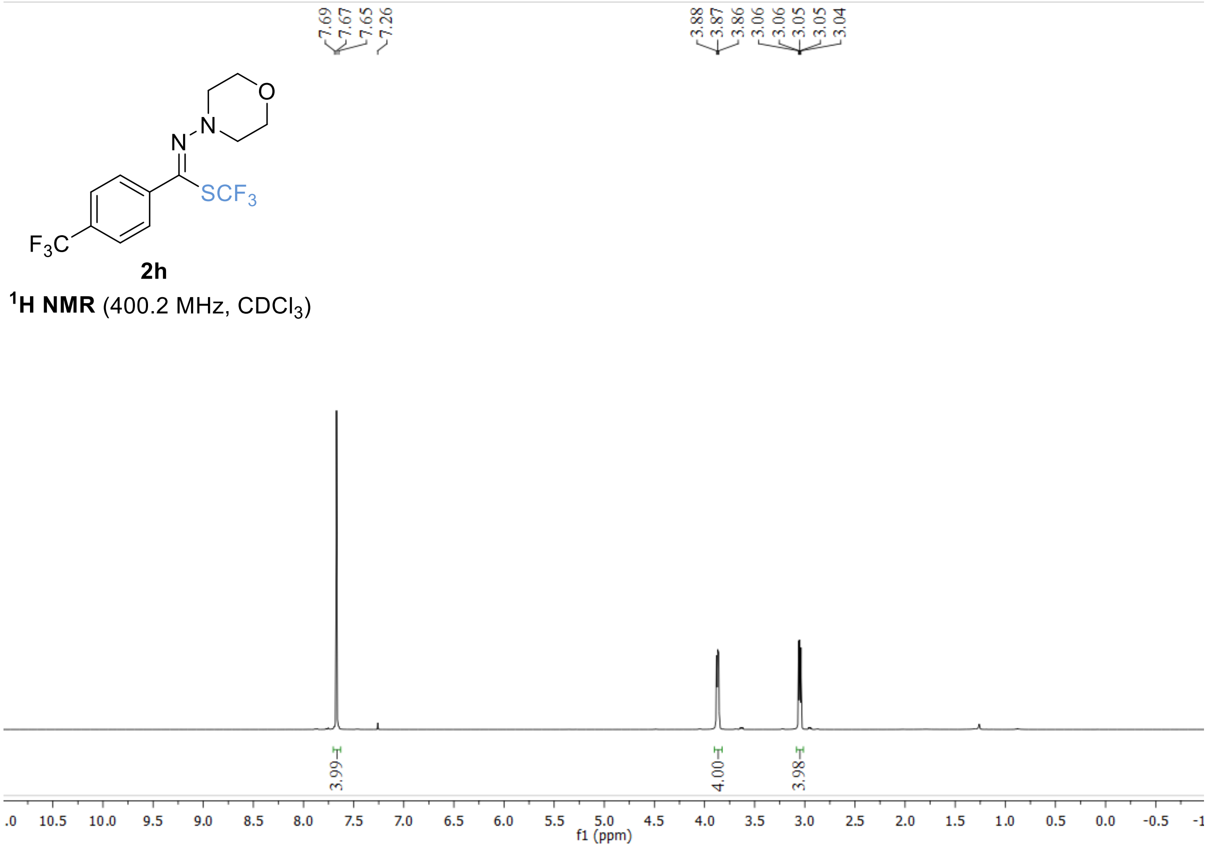

2h

^{1}H NMR (400.2 MHz, $CDCl_3$)

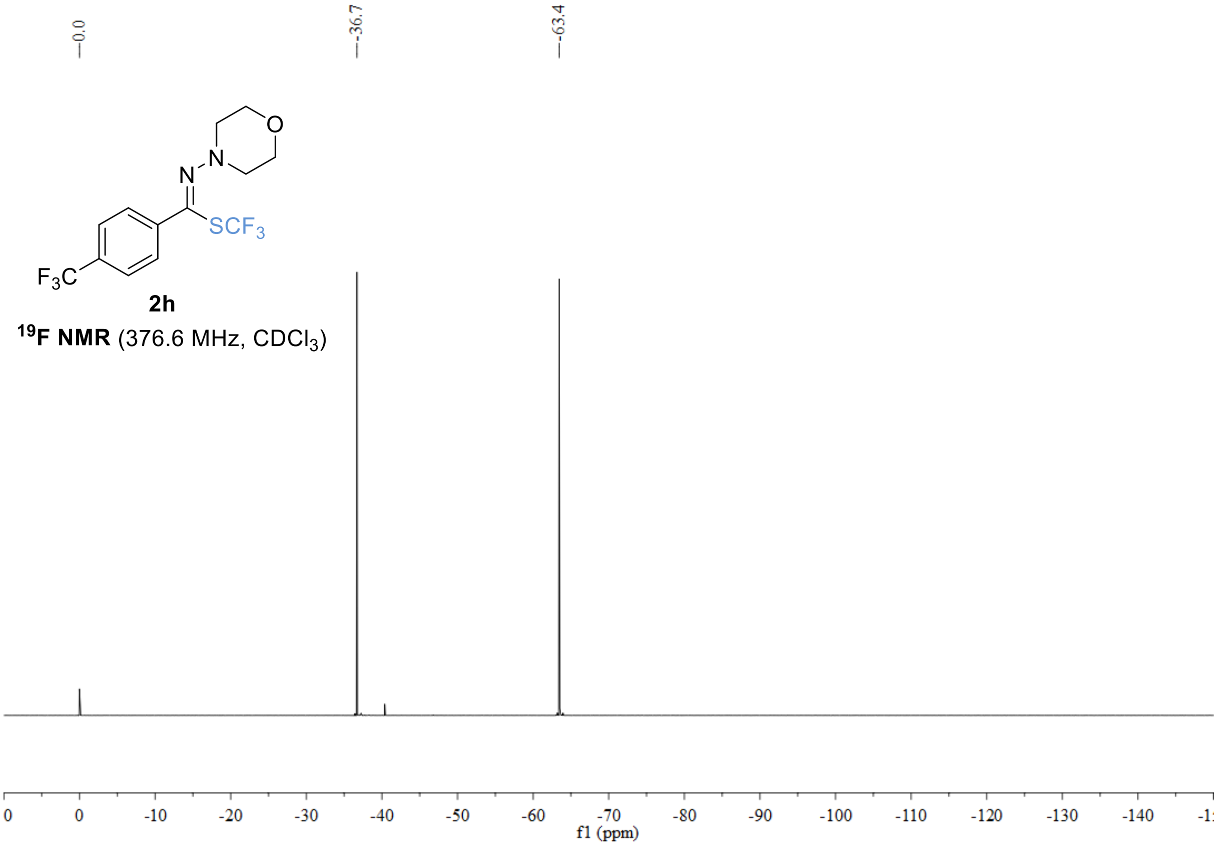

0.0
-36.7
-63.4
N
N
O
SCF3
F3C
2h
19F NMR (376.6 MHz, CDCl3)
f1 (ppm)

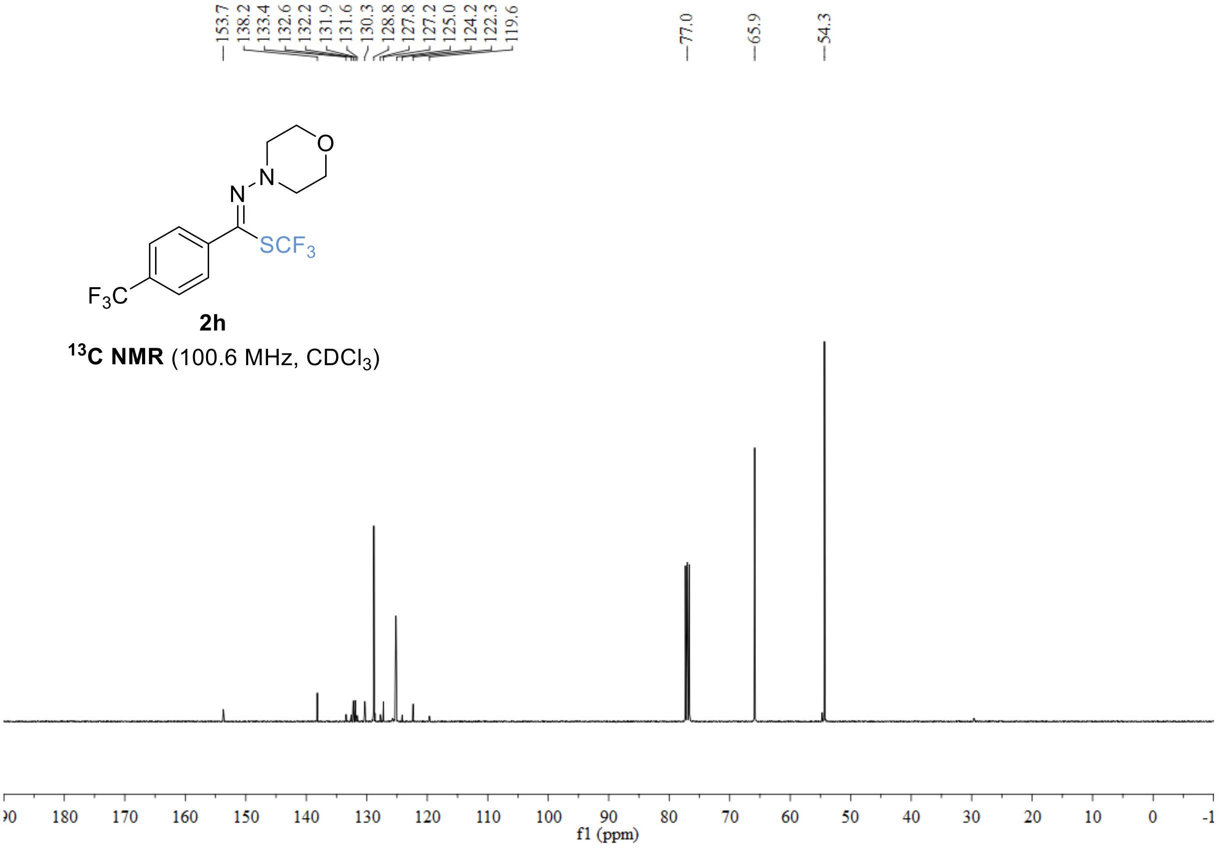

153.7
138.2
133.4
132.6
132.2
131.9
131.6
130.3
128.8
127.8
127.2
125.0
124.2
122.3
119.6
77.0
65.9
54.3
N
N
O
SCF3
F3C
2h
13C NMR (100.6 MHz, CDCl3)
f1 (ppm)

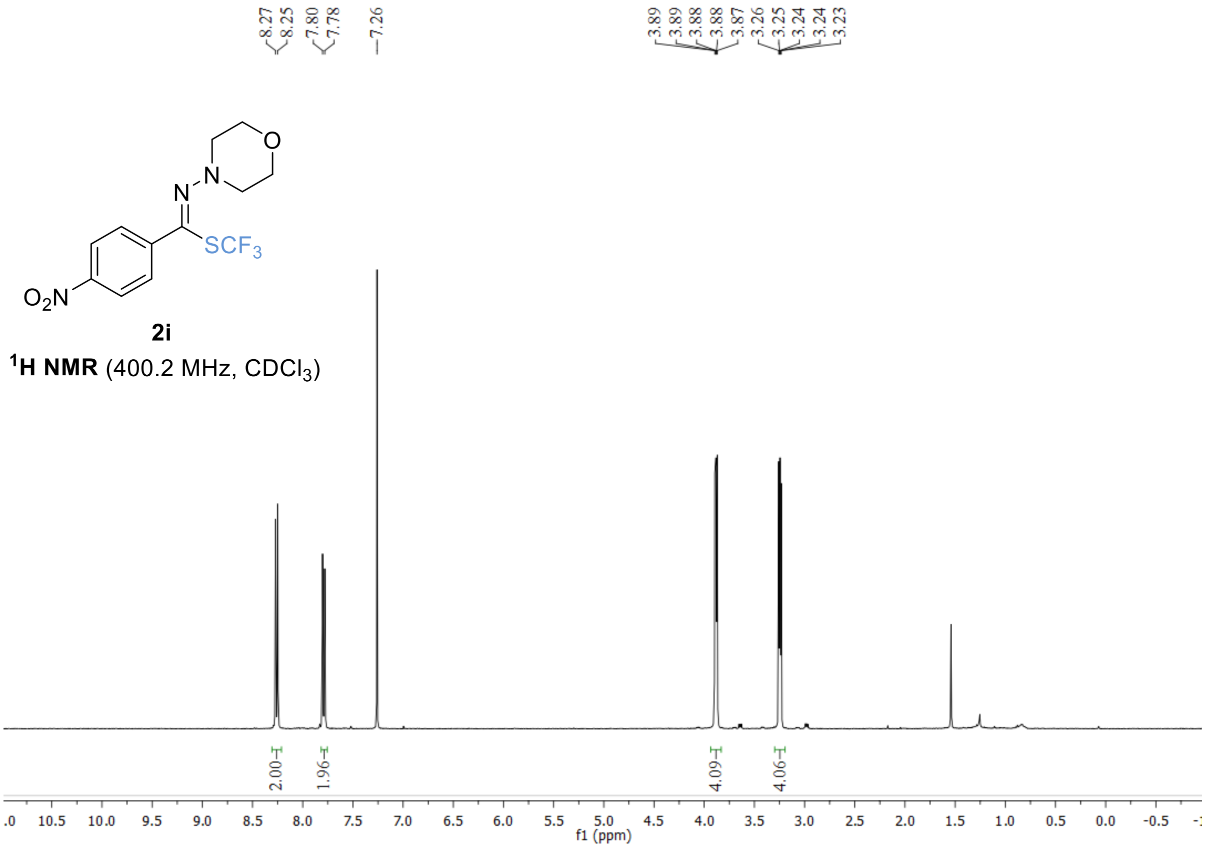
8.27
8.25
7.80
7.78
7.26
3.89
3.89
3.88
3.88
3.87
3.26
3.25
3.24
3.24
3.23
2i
¹H NMR (400.2 MHz, CDCl3)
2.00
1.96
4.09
4.06
f1 (ppm)

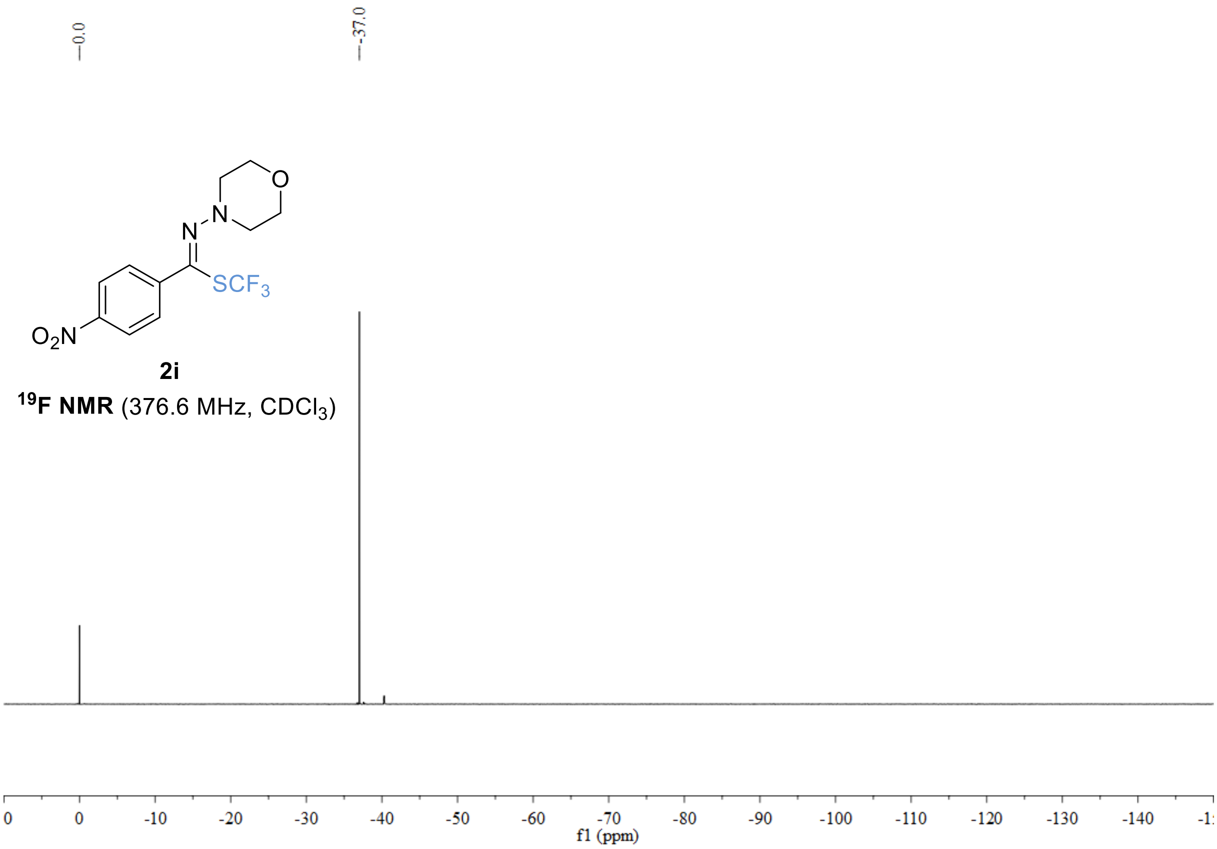
0.0
-37.0
2i
¹⁹F NMR (376.6 MHz, CDCl3)
f1 (ppm)

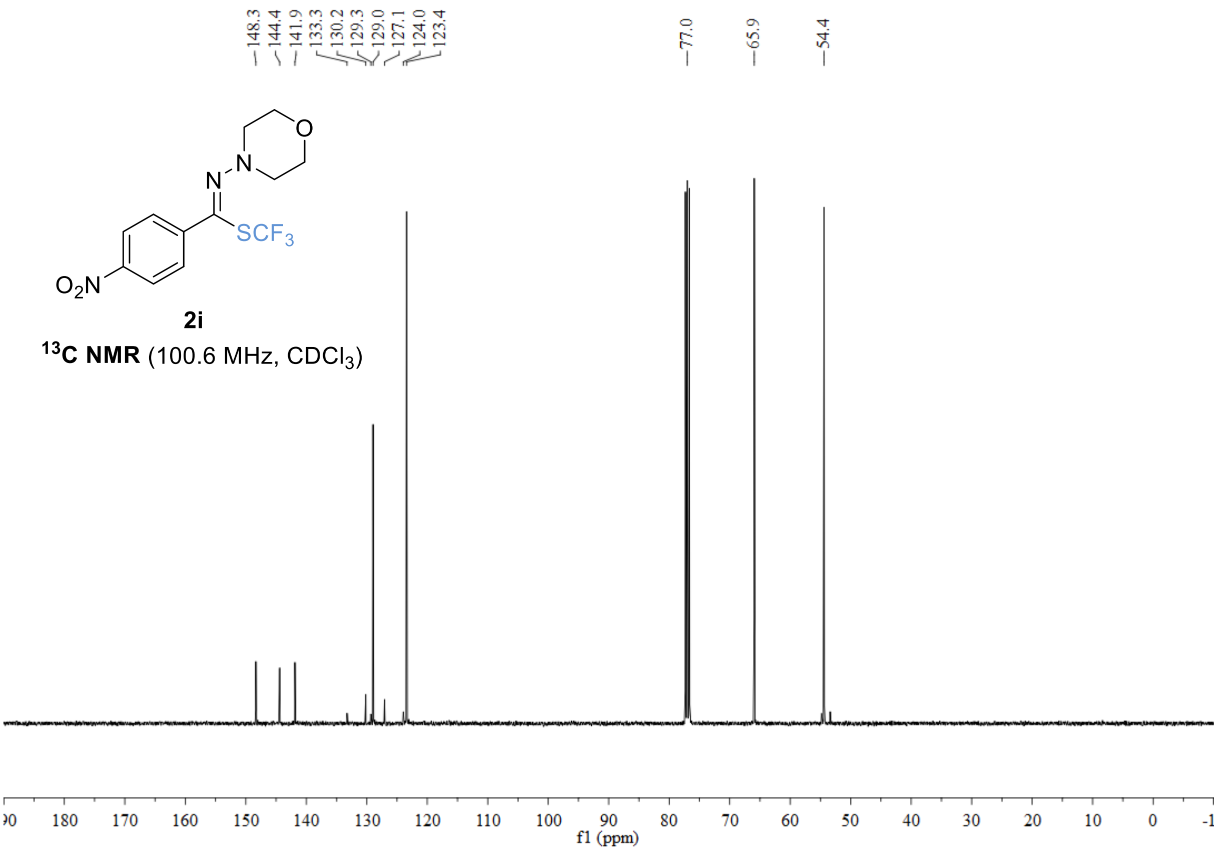

NOESY 2D spectra of compound 2i

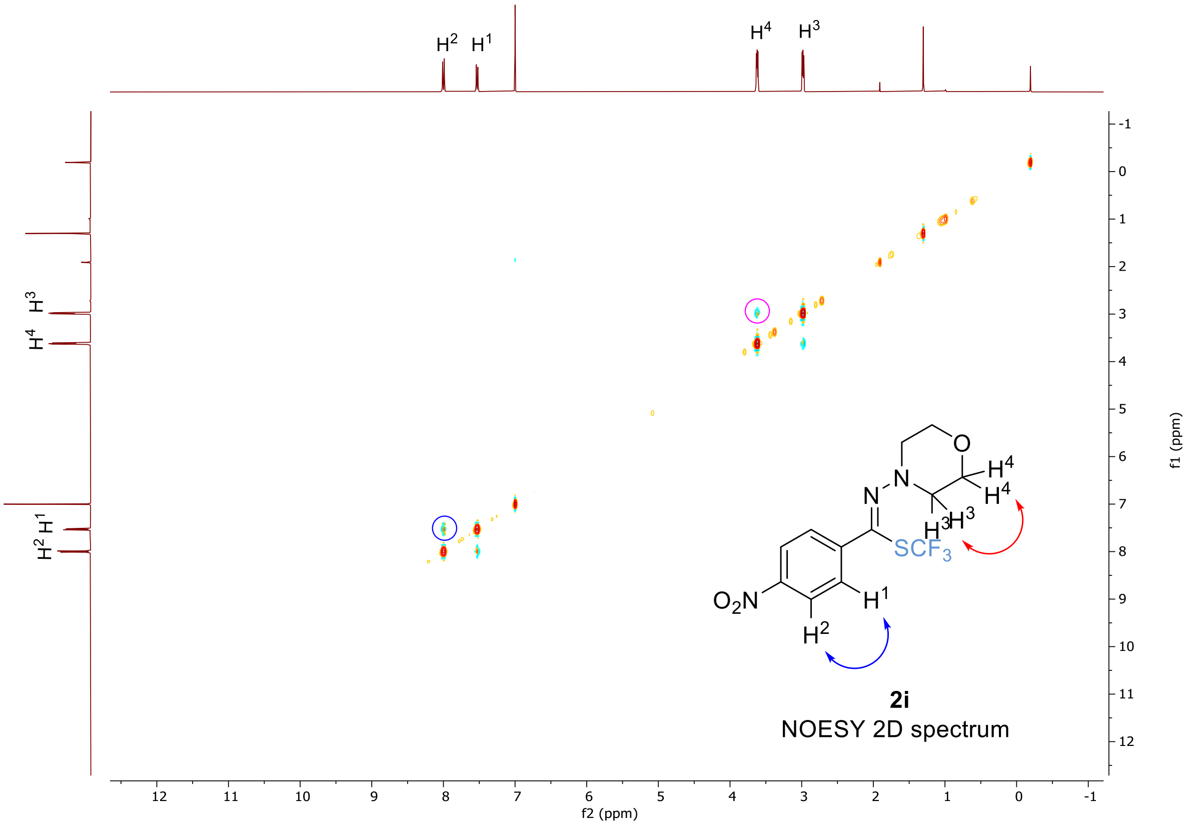

HOESY 2D spectra of compound 2i

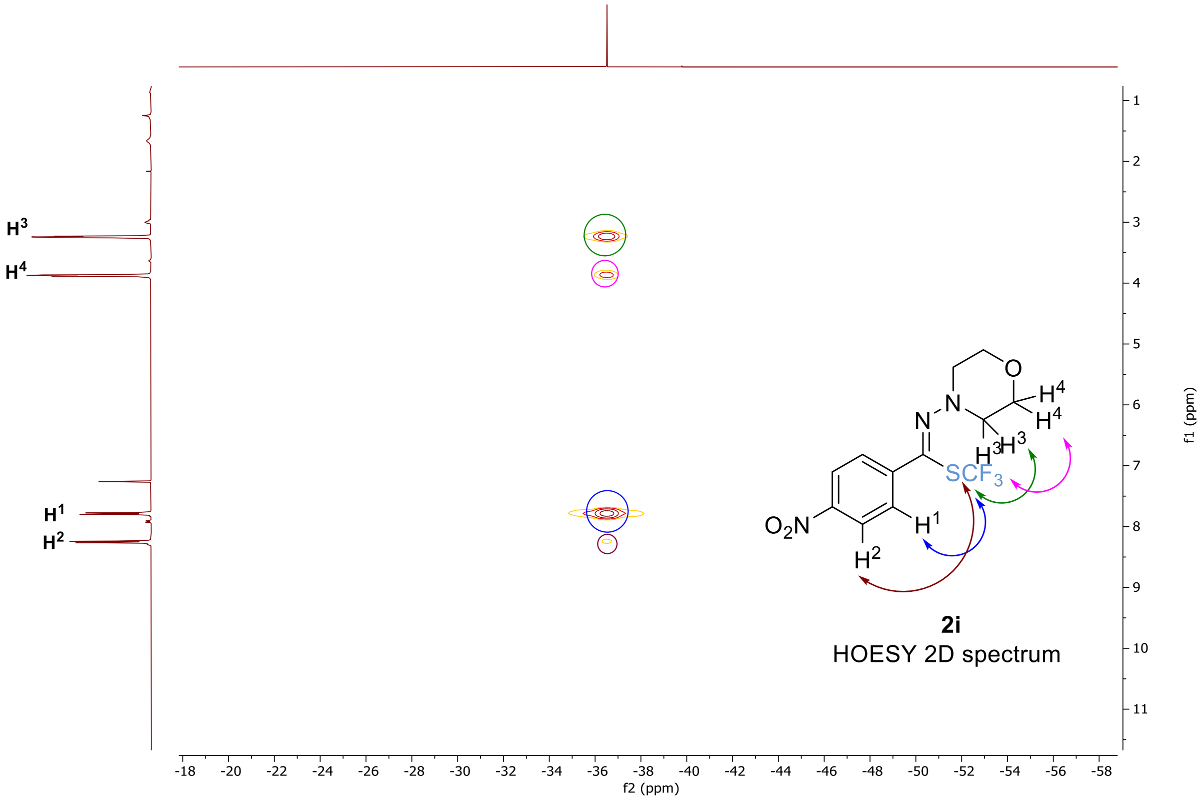

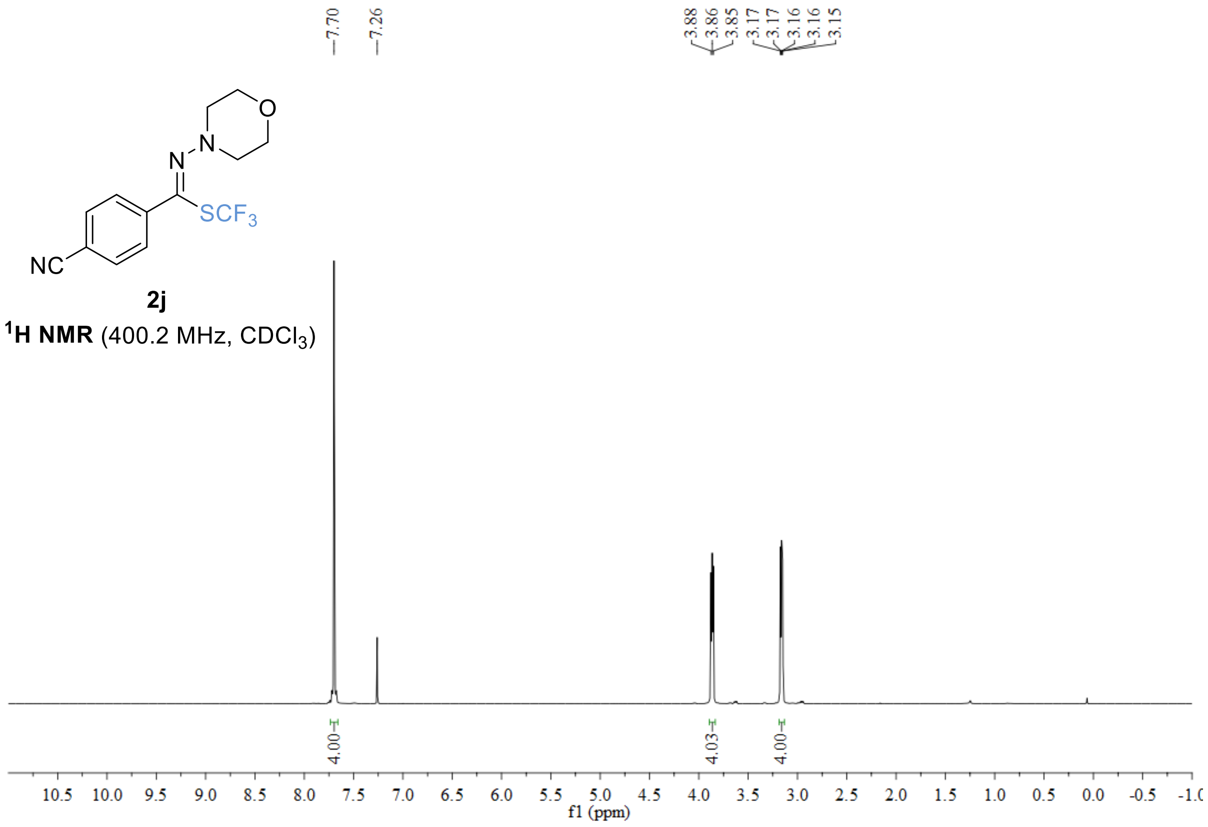

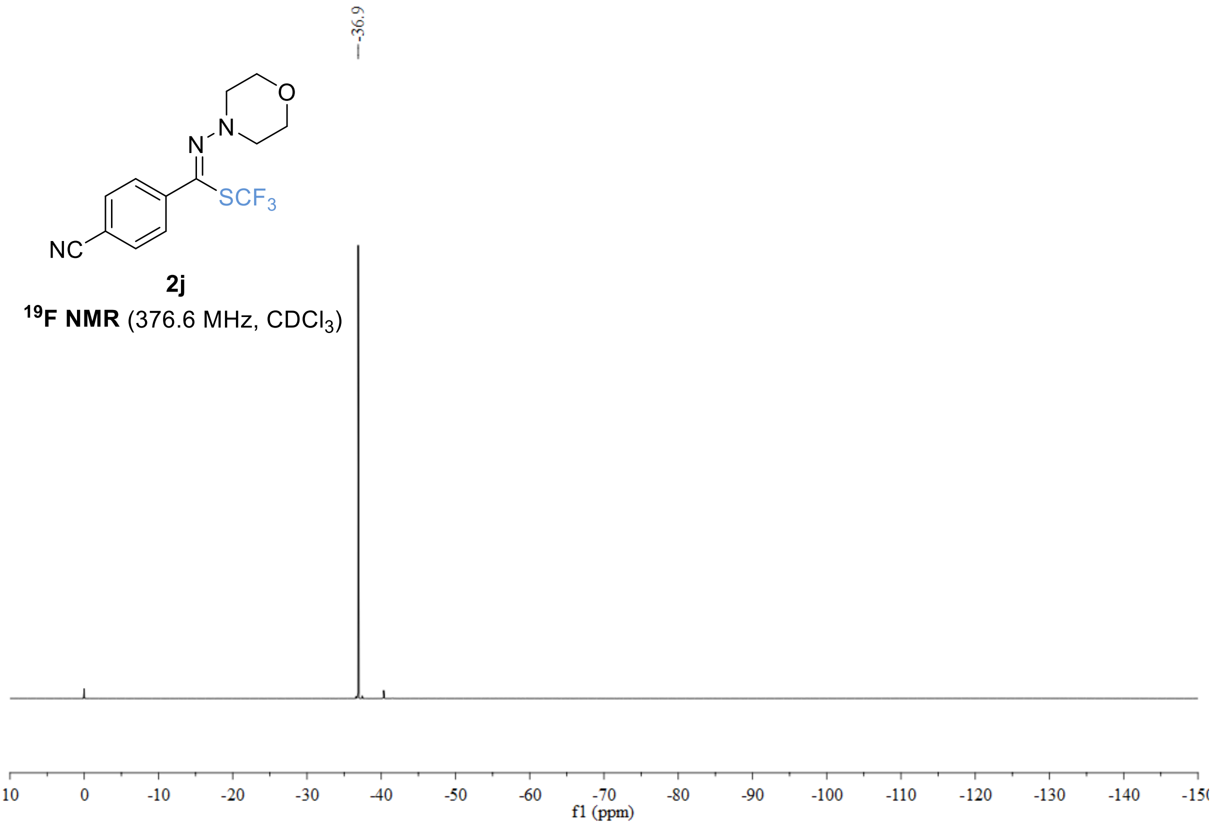

-36.9
N
N
O
SCF3
NC
2j
19F NMR (376.6 MHz, CDCl3)
10 0 -10 -20 -30 -40 -50 -60 -70 -80 -90 -100 -110 -120 -130 -140
f1 (ppm)

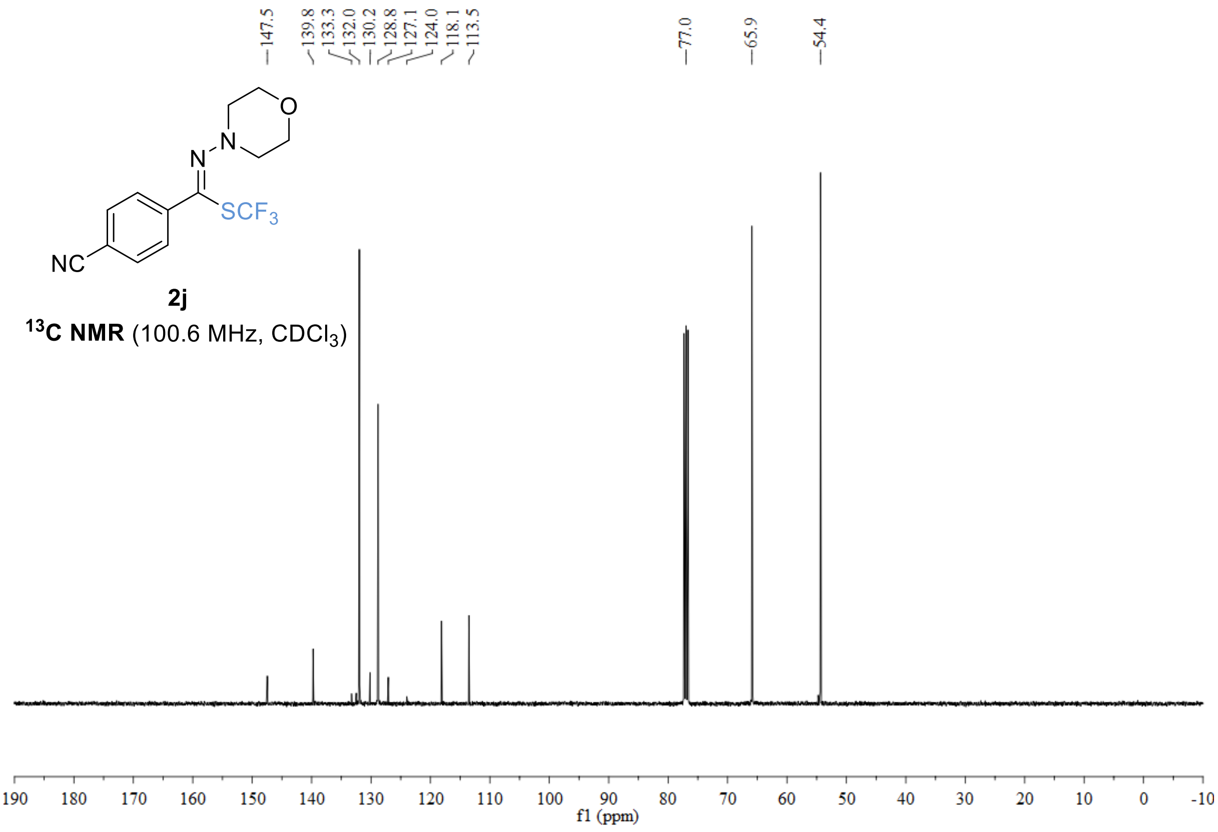

147.5
139.8
133.3
132.0
130.2
128.8
127.1
124.0
118.1
113.5
77.0
65.9
54.4
N
N
O
SCF3
NC
2j
13C NMR (100.6 MHz, CDCl3)
190 180 170 160 150 140 130 120 110 100 90 80 70 60 50 40 30 20 10 0 -10
f1 (ppm)

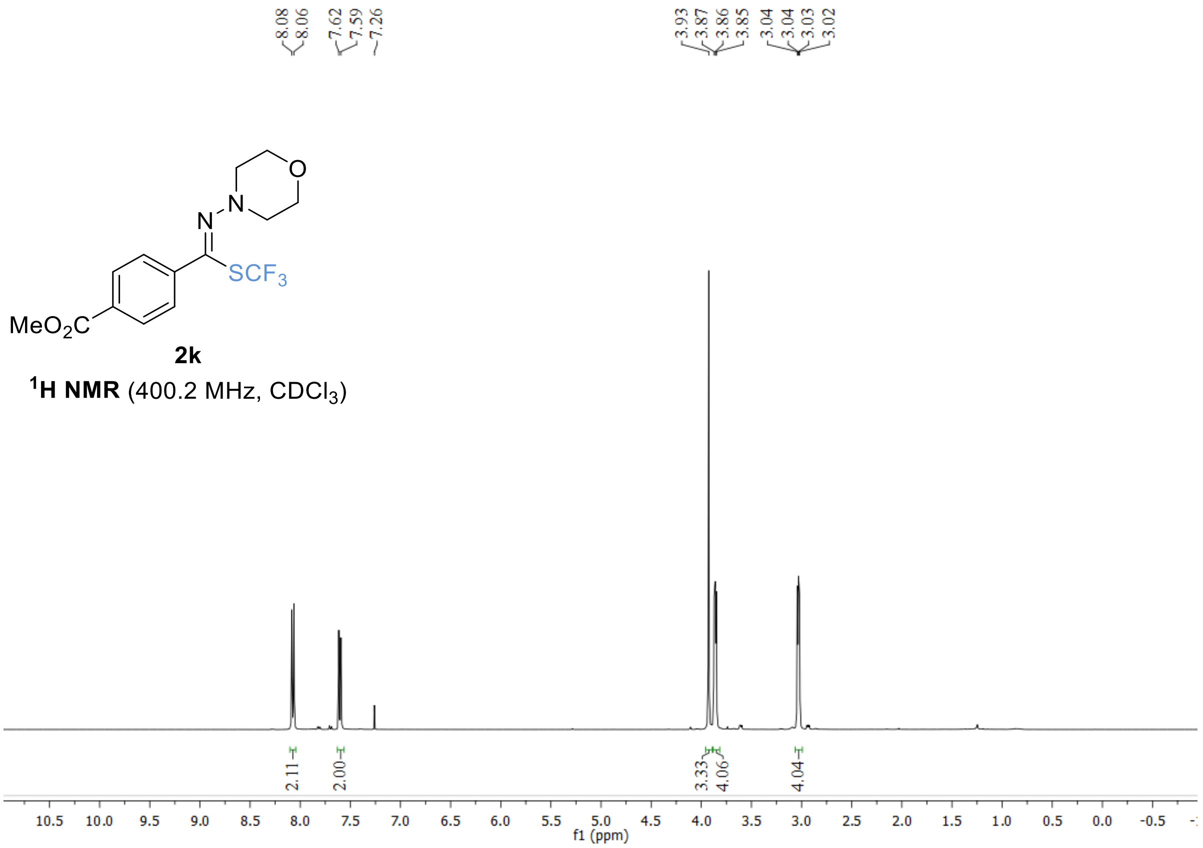

8.08
8.06
7.62
7.59
7.26
3.93
3.87
3.86
3.85
3.04
3.04
3.03
3.02
MeO2C
N
N
O
SCF3
2k
1H NMR (400.2 MHz, CDCl3)
2.11
2.00
3.33
4.06
4.04
f1 (ppm)

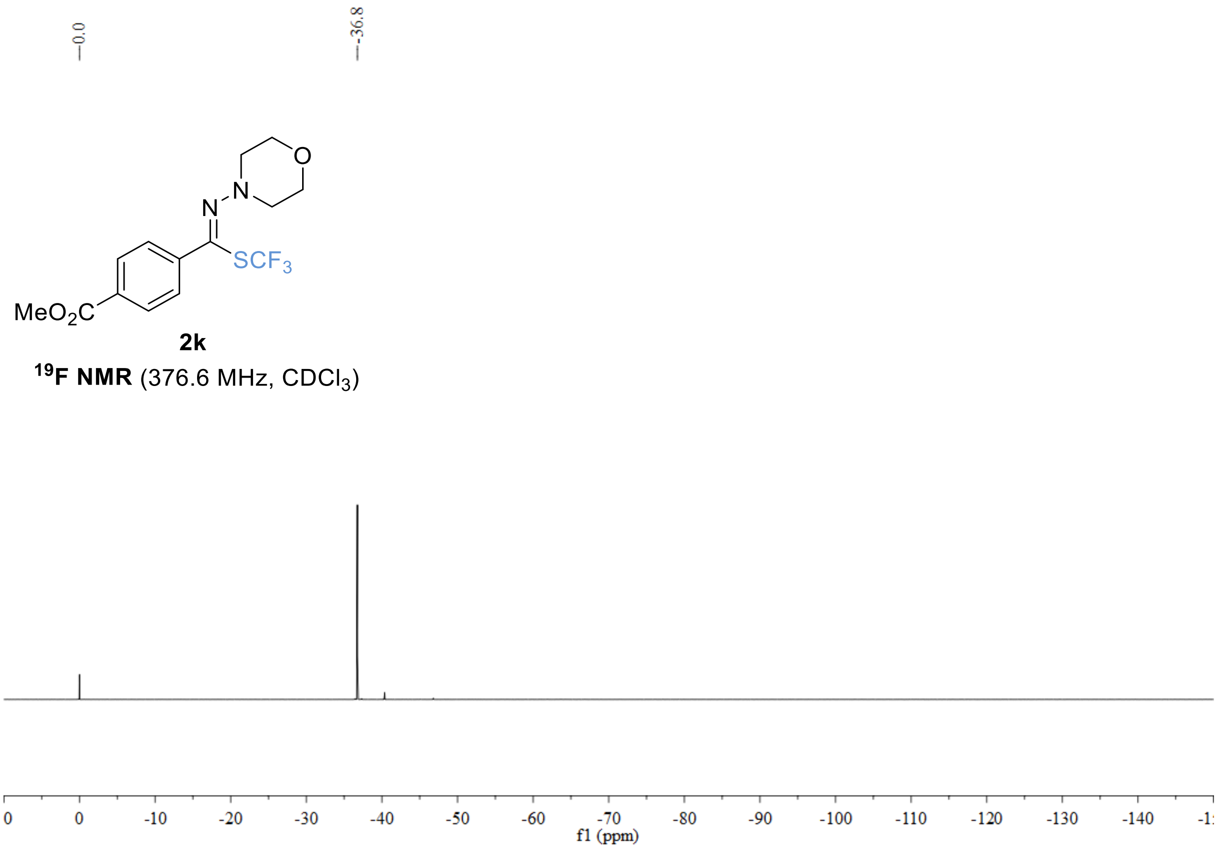

0.0
-36.8
MeO2C
N
N
O
SCF3
2k
19F NMR (376.6 MHz, CDCl3)
f1 (ppm)

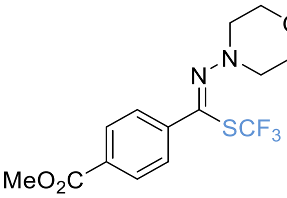

2k

^{13}**C NMR** (100.6 MHz, $CDCl_3$)

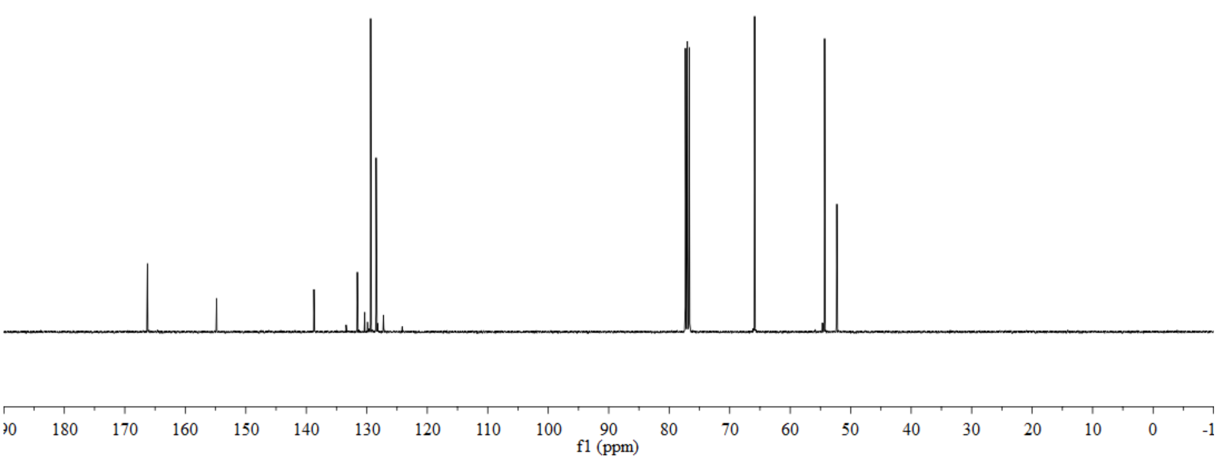

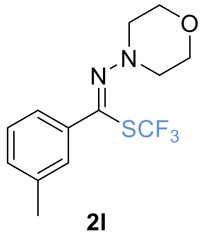

2l

^{1}**H NMR** (400.2 MHz, $CDCl_3$)

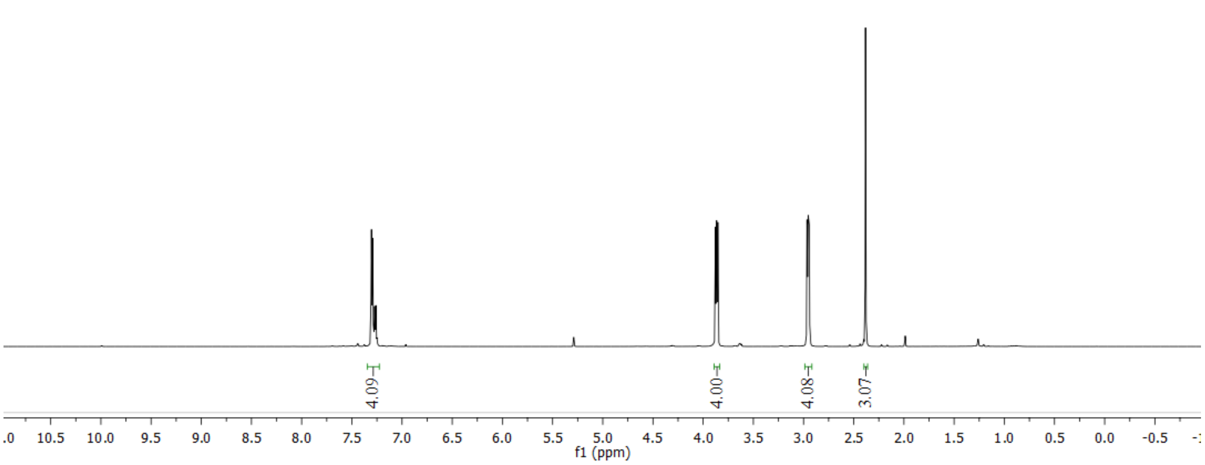

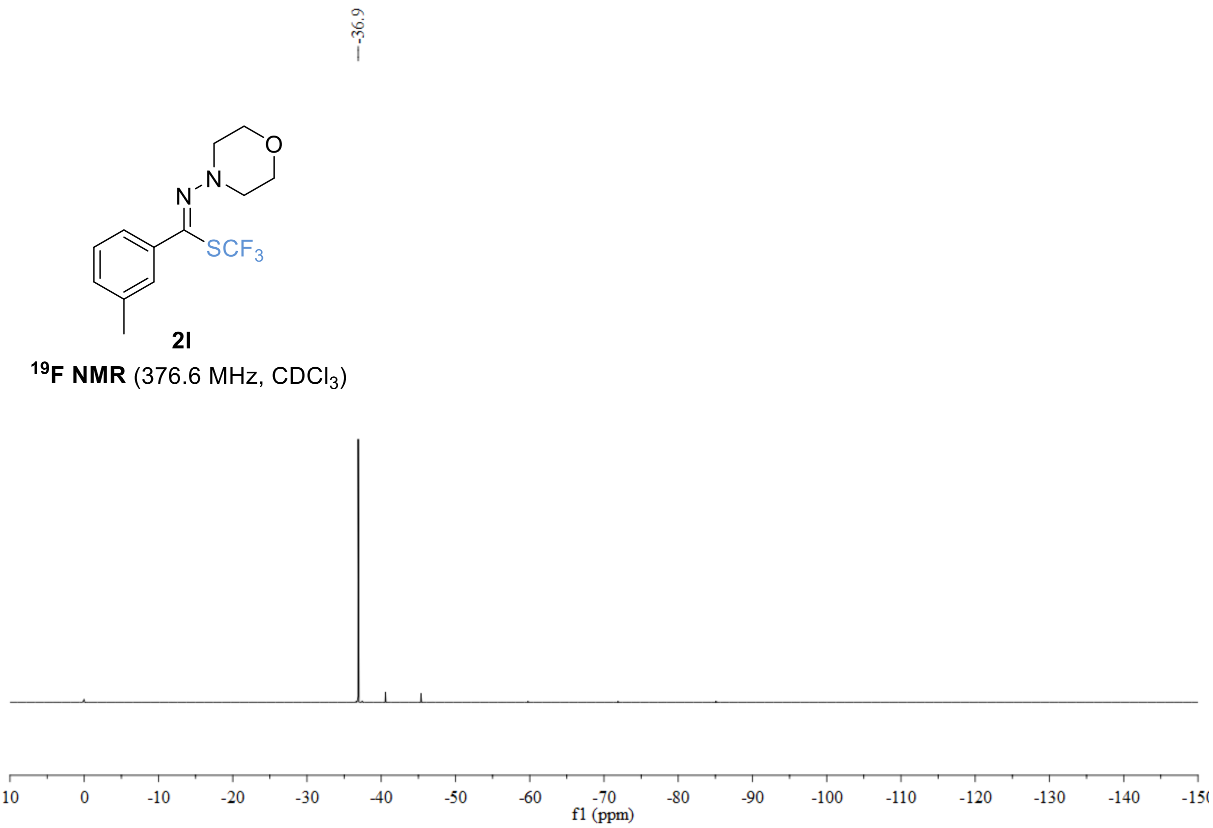

2l

^{19}**F NMR** (376.6 MHz, $CDCl_3$)

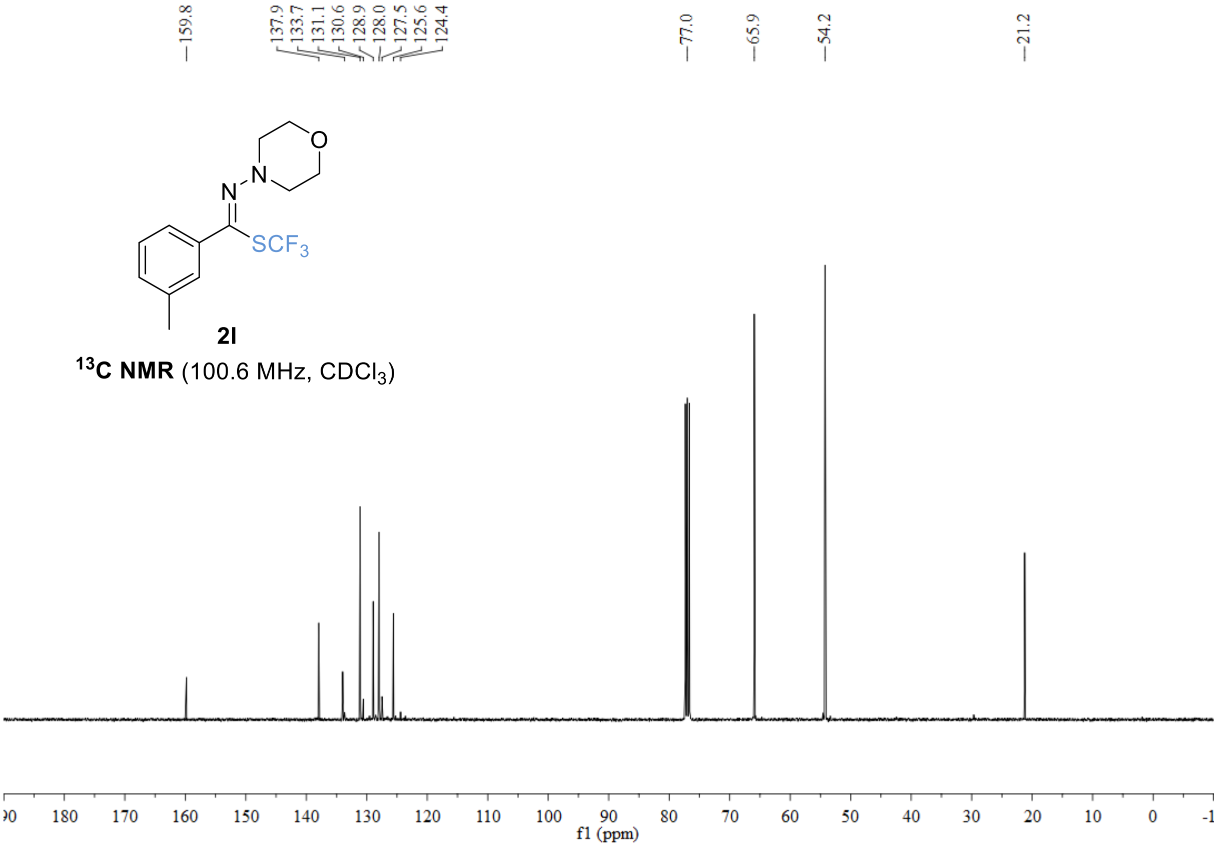

2l

^{13}**C NMR** (100.6 MHz, $CDCl_3$)

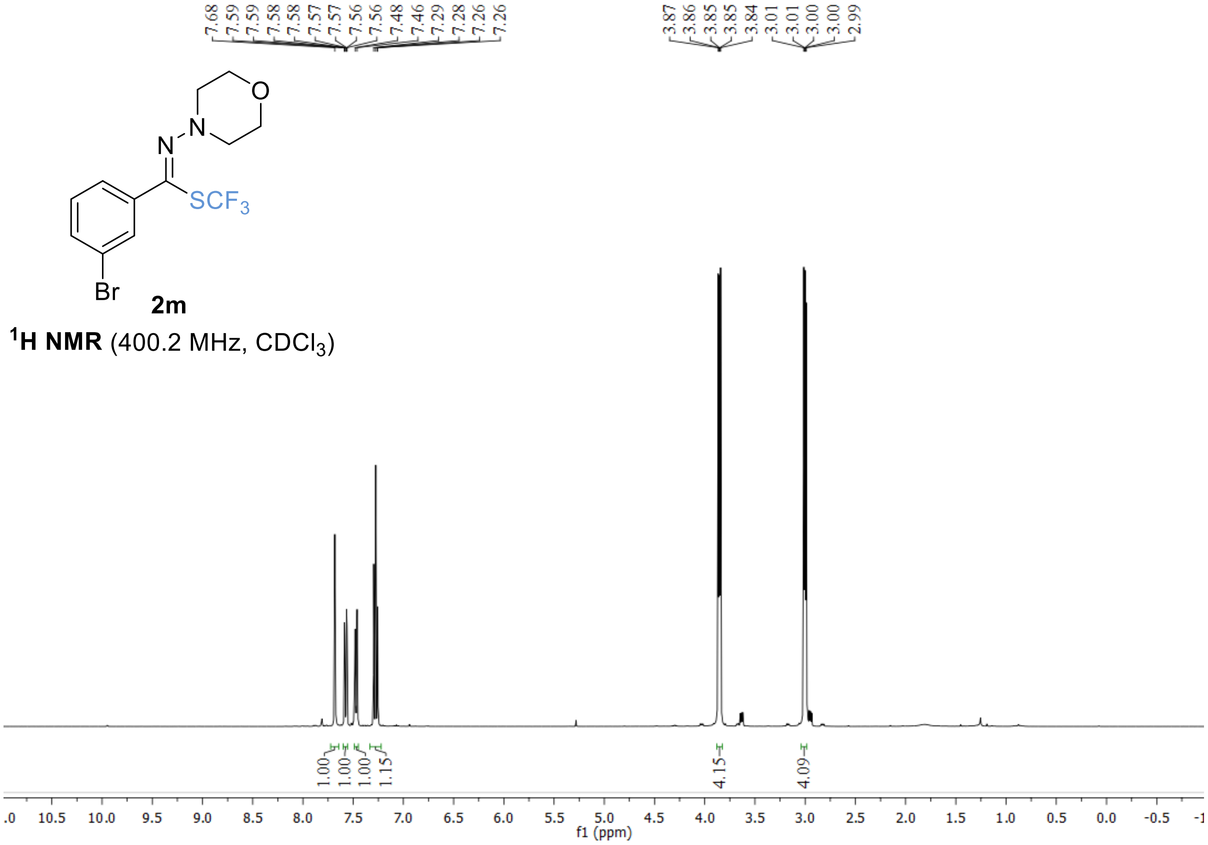

7.68 7.59 7.59 7.58 7.58 7.57 7.57 7.56 7.56 7.48 7.46 7.29 7.28 7.26 7.26
3.87 3.86 3.85 3.85 3.84 3.01 3.01 3.00 3.00 2.99
O
N
N
SCF3
Br
2m
1H NMR (400.2 MHz, CDCl3)
1.00 1.00 1.00 1.15 4.15 4.09
f1 (ppm)

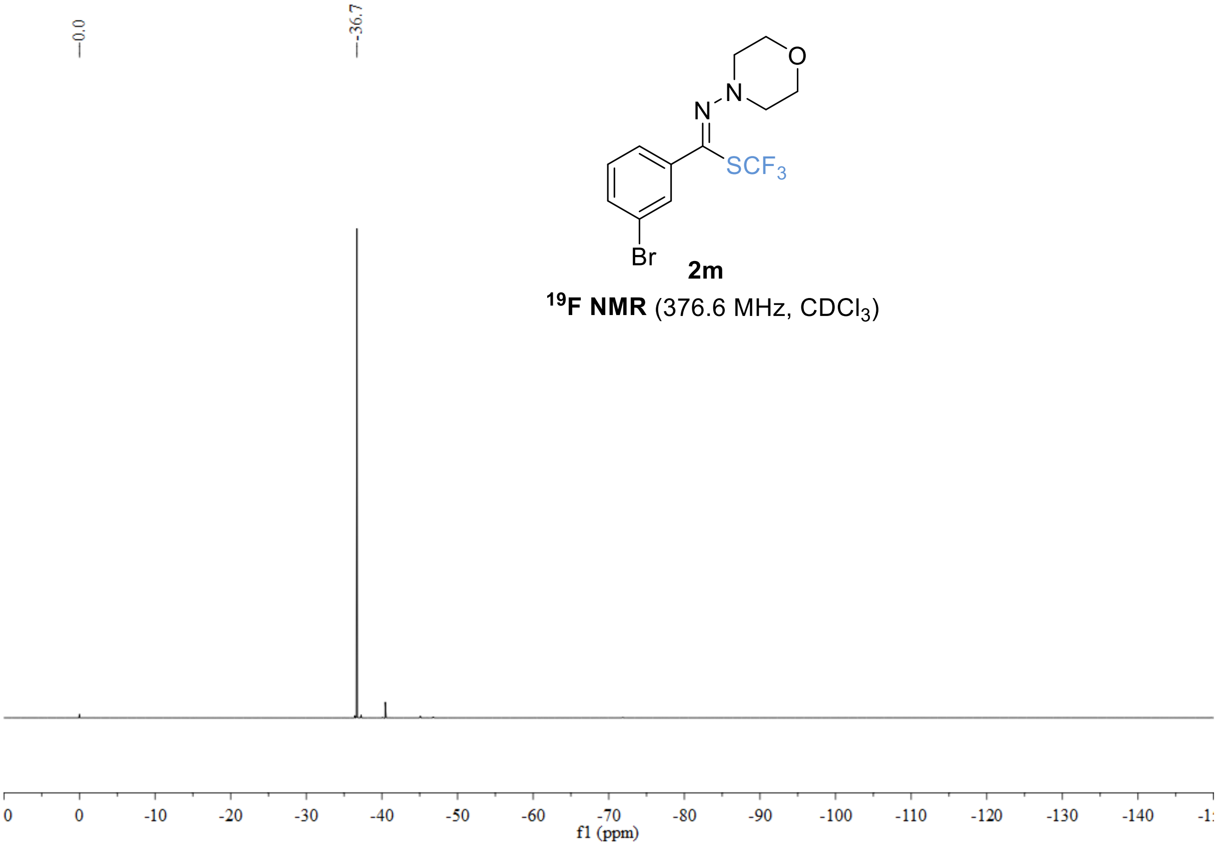

0.0
-36.7
O
N
N
SCF3
Br
2m
19F NMR (376.6 MHz, CDCl3)
f1 (ppm)

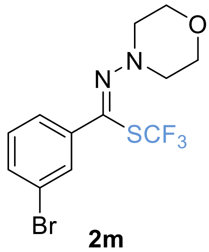

2m

^{13}C NMR (100.6 MHz, $CDCl_3$)

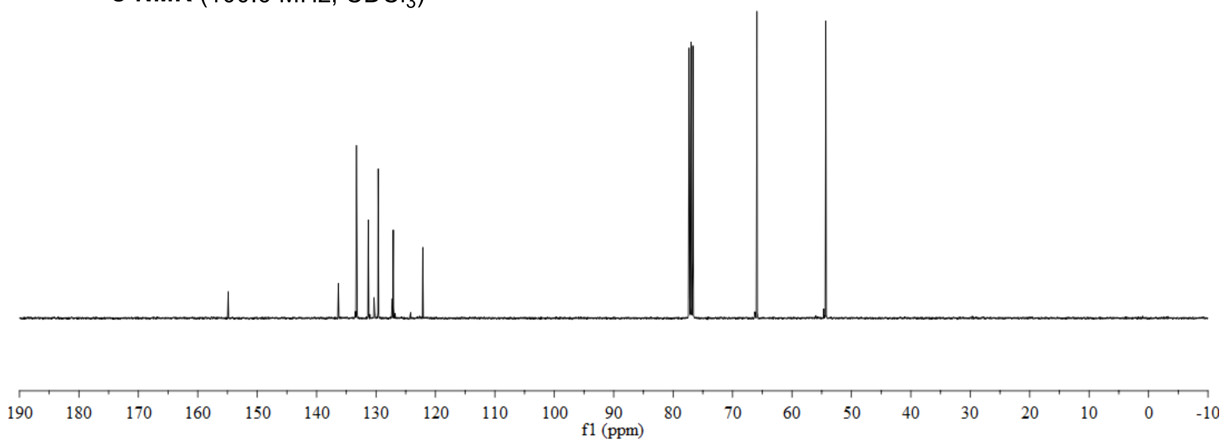

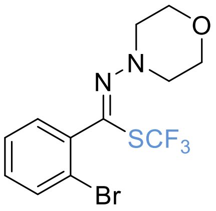

2n

^{1}H NMR (400.2 MHz, $CDCl_3$)

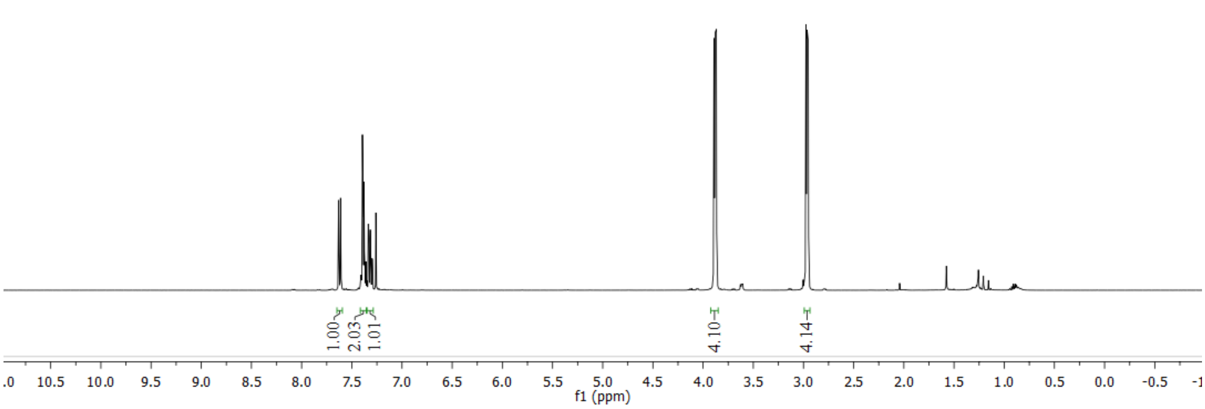

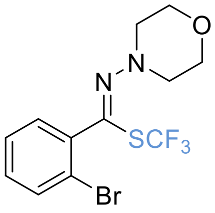

2n

^{19}F NMR (376.6 MHz, $CDCl_3$)

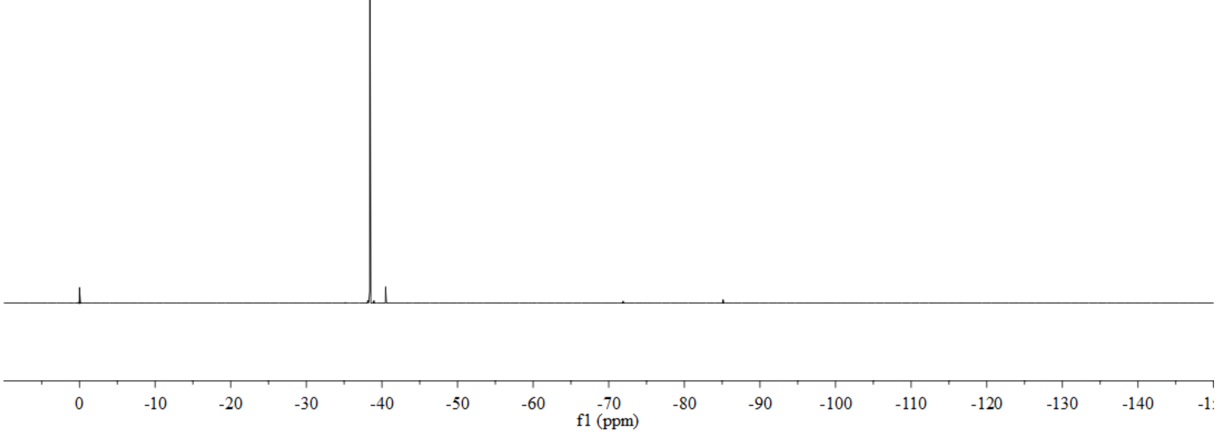

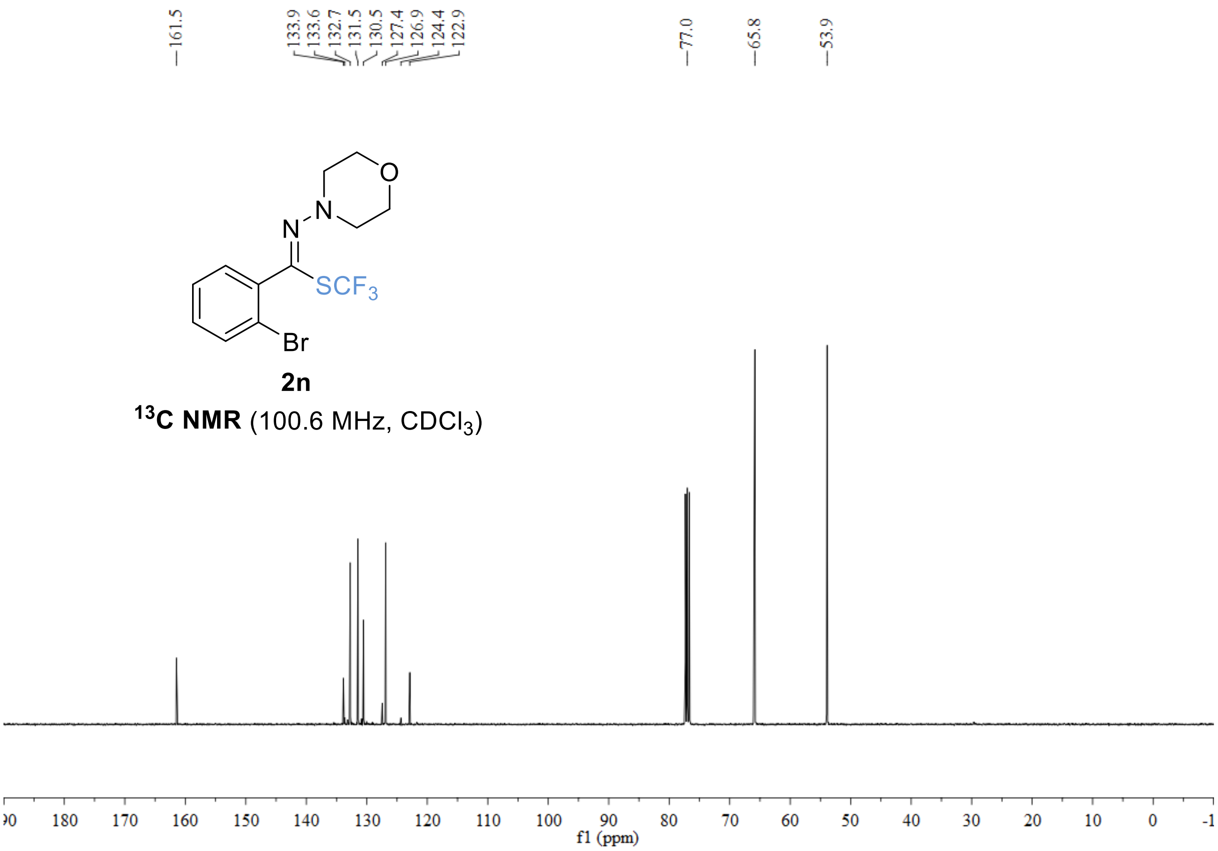
161.5
133.9
133.6
132.7
131.5
130.5
127.4
126.9
124.4
122.9
77.0
65.8
53.9
N
N
O
SCF3
Br
2n
13C NMR (100.6 MHz, CDCl3)
f1 (ppm)

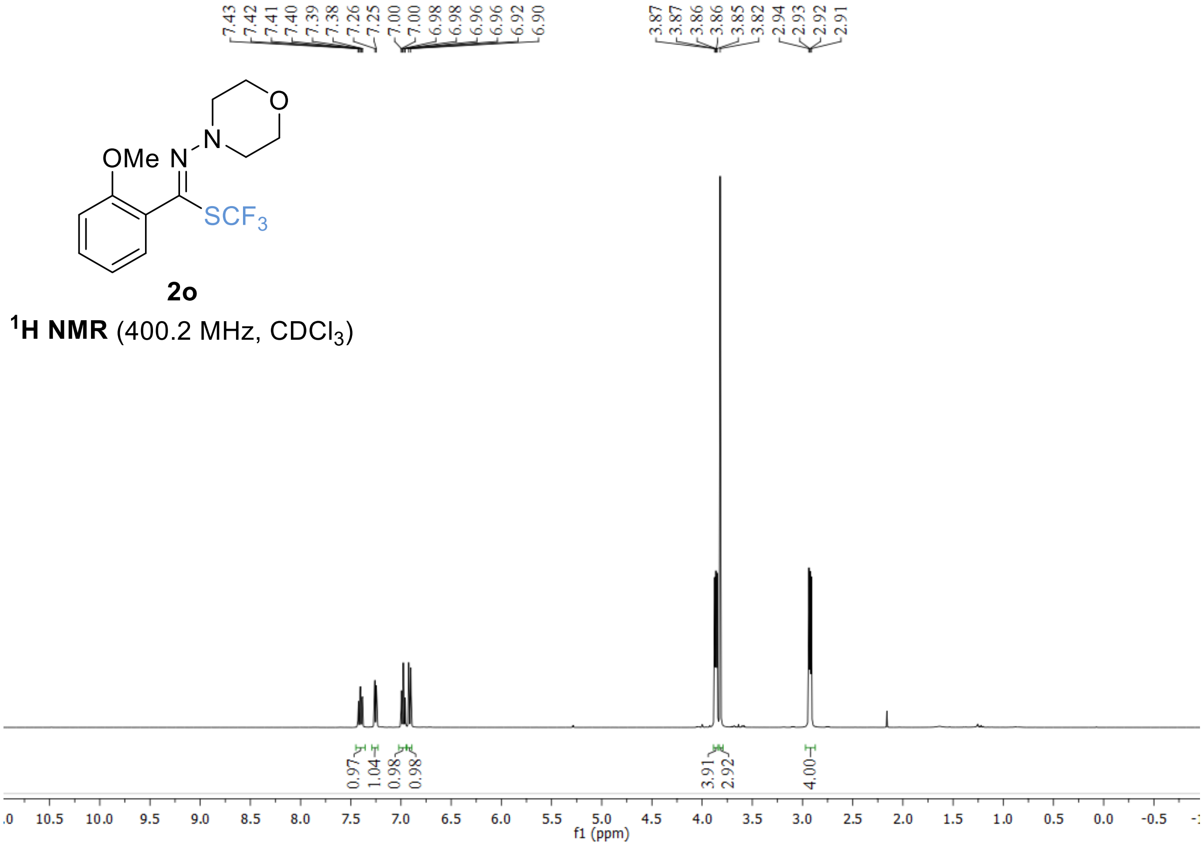
7.43
7.42
7.41
7.40
7.39
7.38
7.26
7.25
7.00
7.00
6.98
6.98
6.96
6.96
6.92
6.90
3.87
3.87
3.86
3.86
3.85
3.82
2.94
2.93
2.92
2.91
OMe
N
N
O
SCF3
2o
1H NMR (400.2 MHz, CDCl3)
0.97
1.04
0.98
0.98
3.91
2.92
4.00
f1 (ppm)

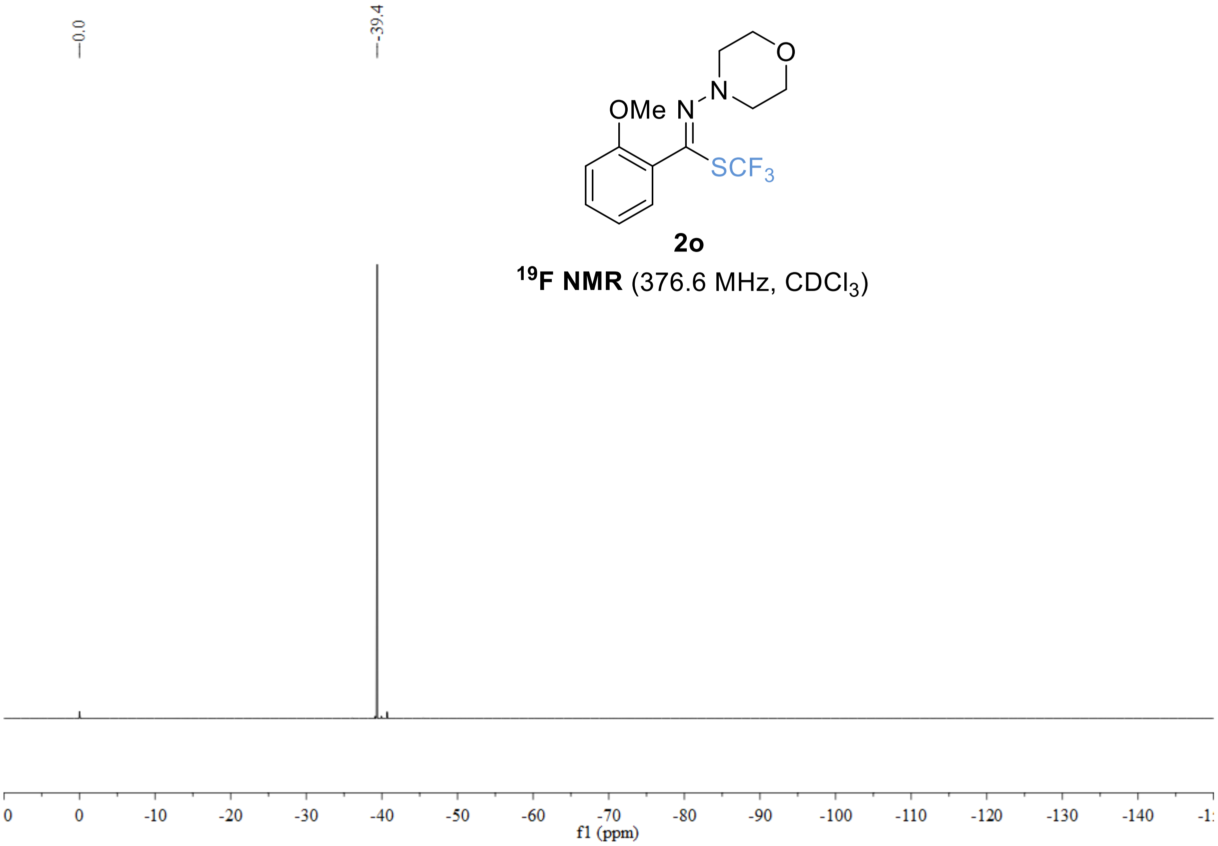
-0.0
-39.4
OMe
N
N
O
SCF3
2o
19F NMR (376.6 MHz, CDCl3)
f1 (ppm)

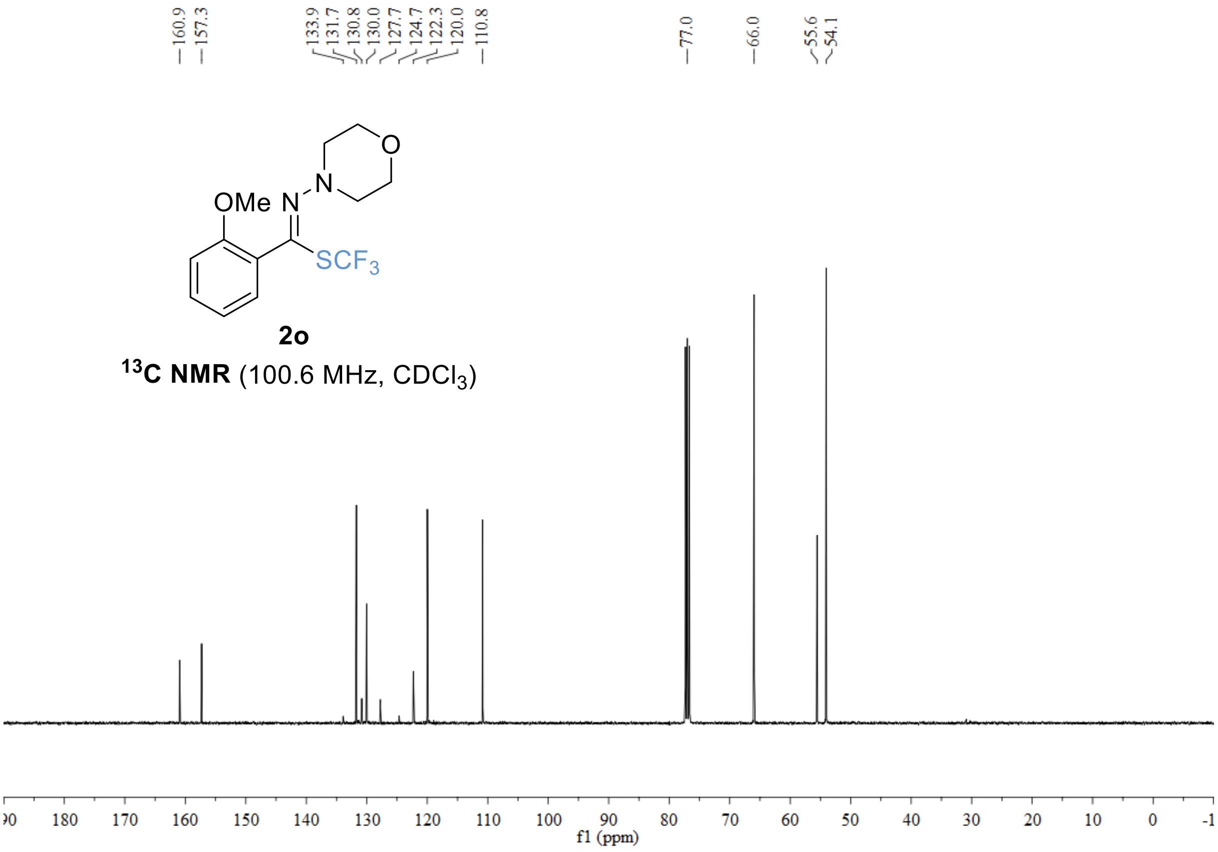
160.9
157.3
133.9
131.7
130.8
130.0
127.7
124.7
122.3
120.0
110.8
77.0
66.0
55.6
54.1
OMe
N
N
O
SCF3
2o
13C NMR (100.6 MHz, CDCl3)
f1 (ppm)

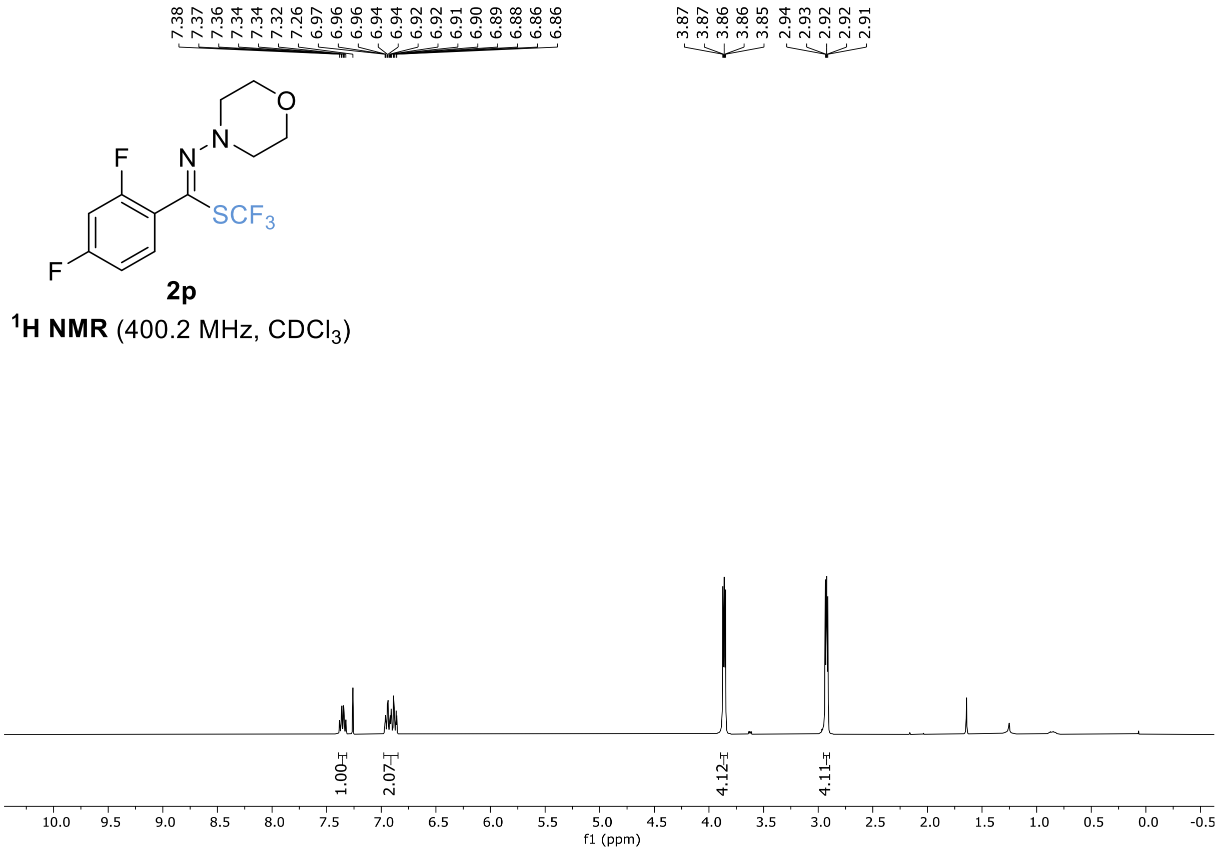

2p

^{1}H NMR (400.2 MHz, $CDCl_3$)

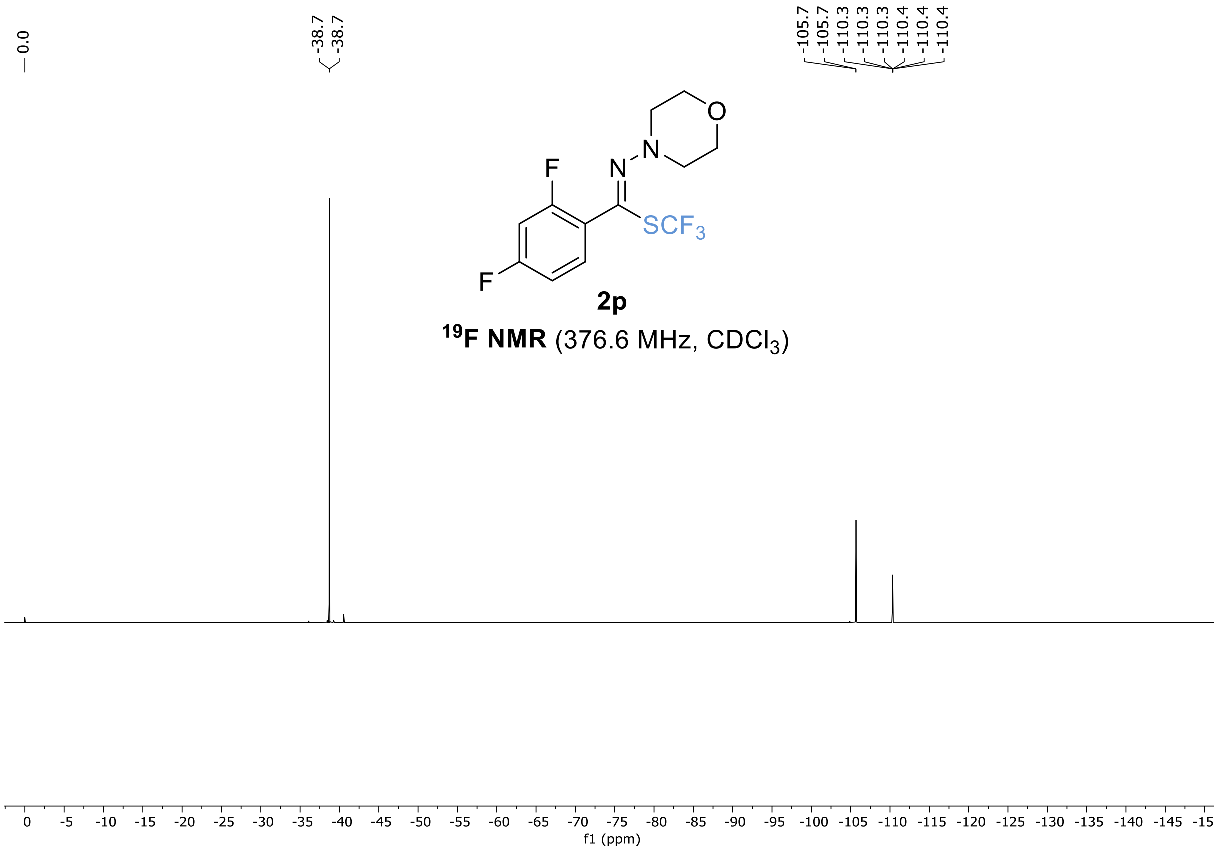

2p

^{19}F NMR (376.6 MHz, $CDCl_3$)

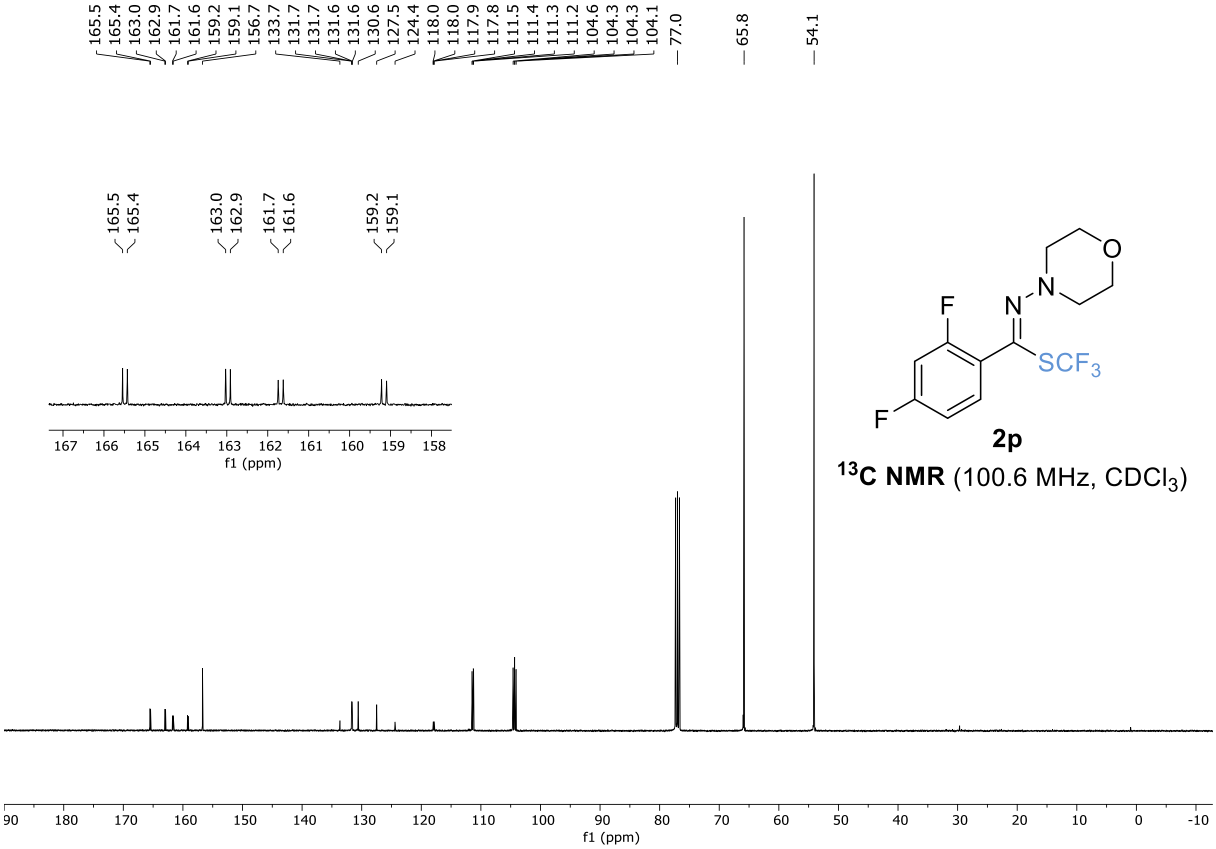

2p

^{13}C NMR (100.6 MHz, $CDCl_3$)

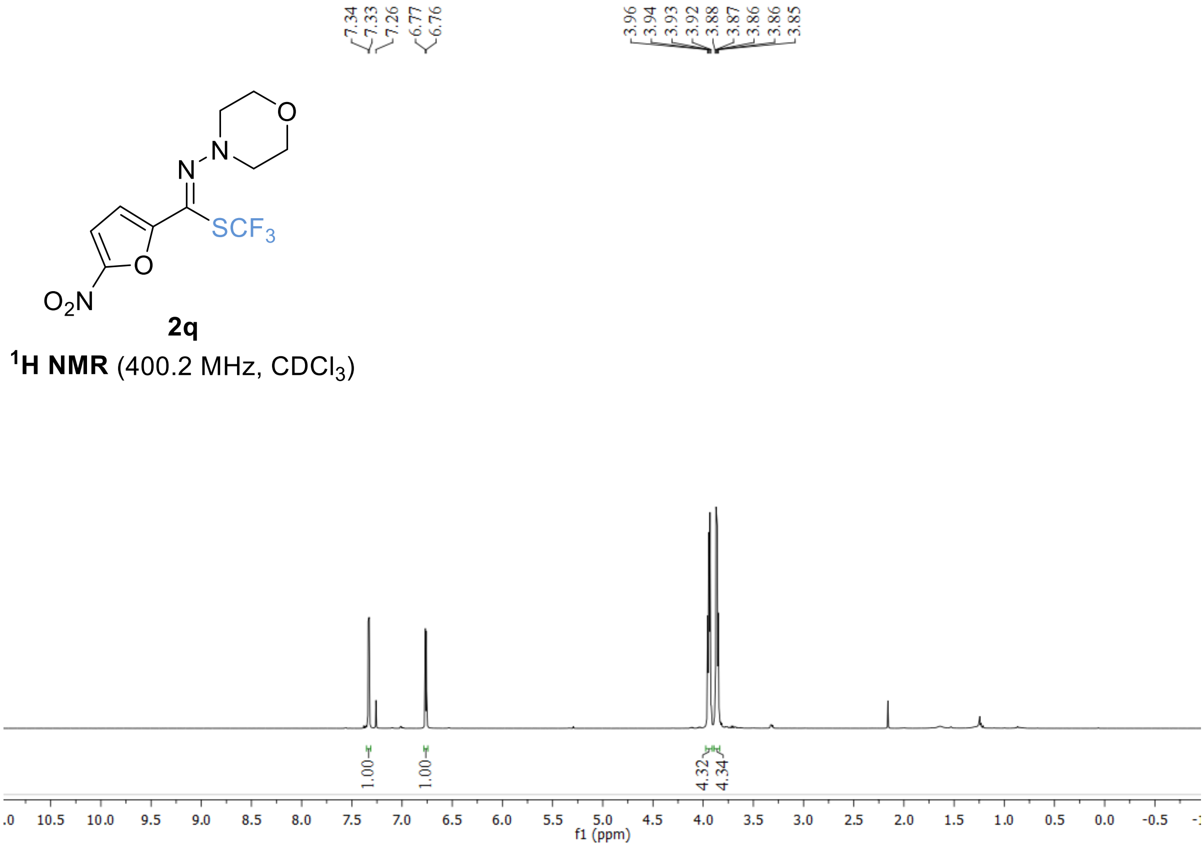

2q

^{1}H NMR (400.2 MHz, $CDCl_3$)

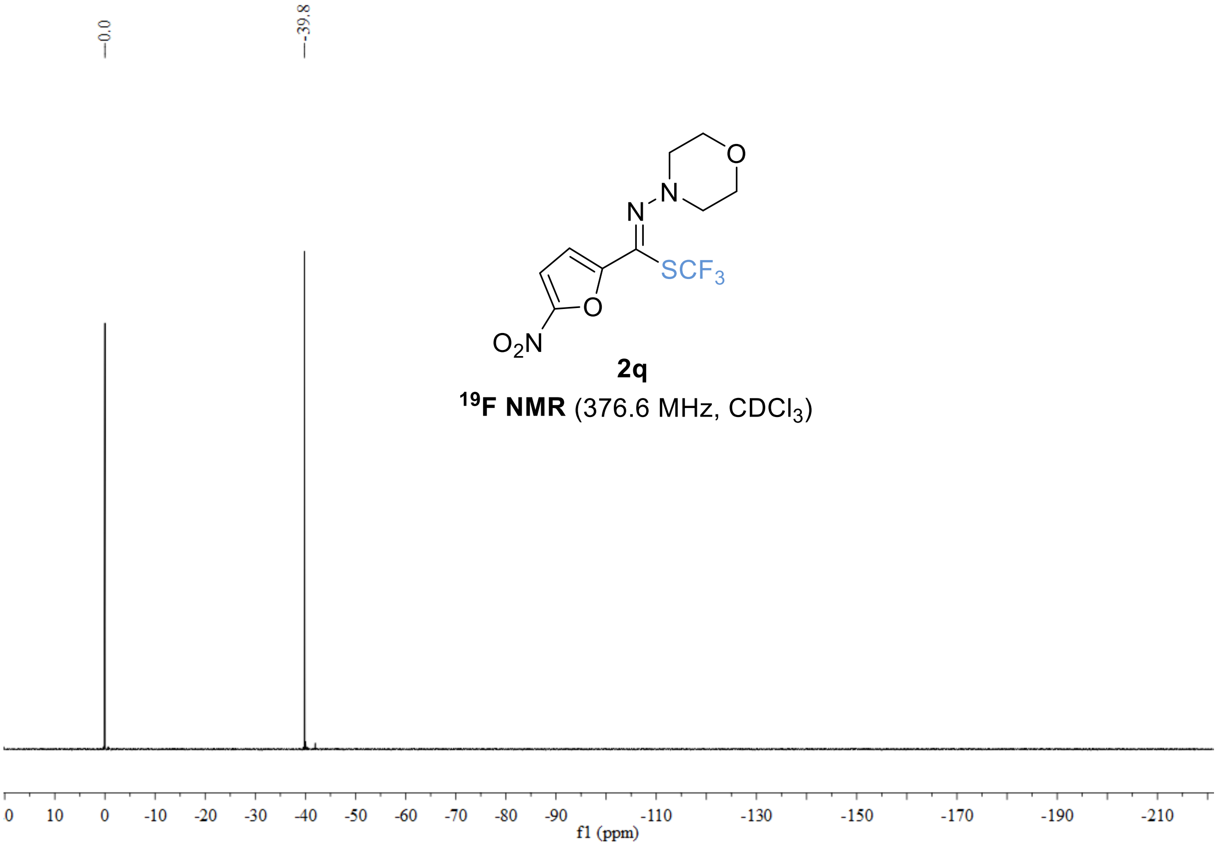

—0.0
—-39.8
SCF3
O2N
2q
19F NMR (376.6 MHz, CDCl3)
f1 (ppm)

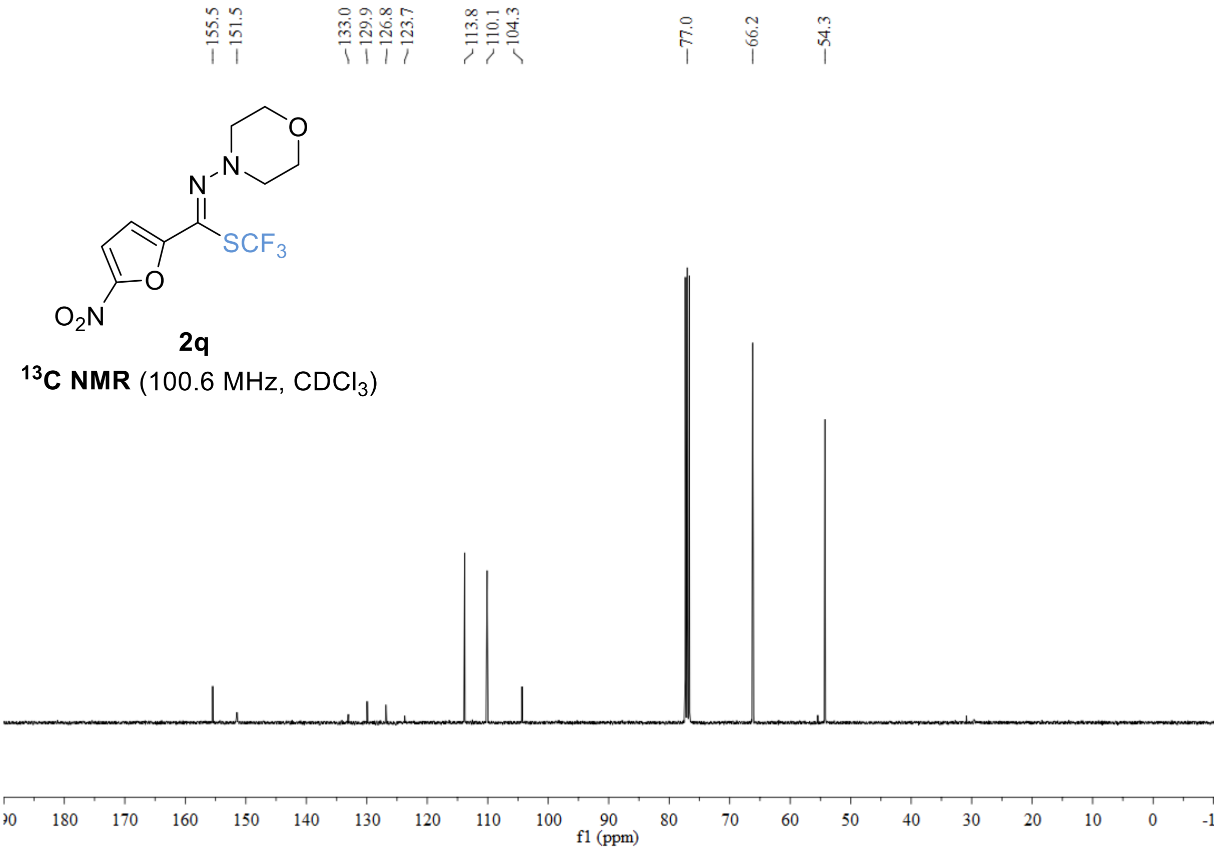

155.5
151.5
133.0
129.9
126.8
123.7
113.8
110.1
104.3
77.0
66.2
54.3
SCF3
O2N
2q
13C NMR (100.6 MHz, CDCl3)
f1 (ppm)

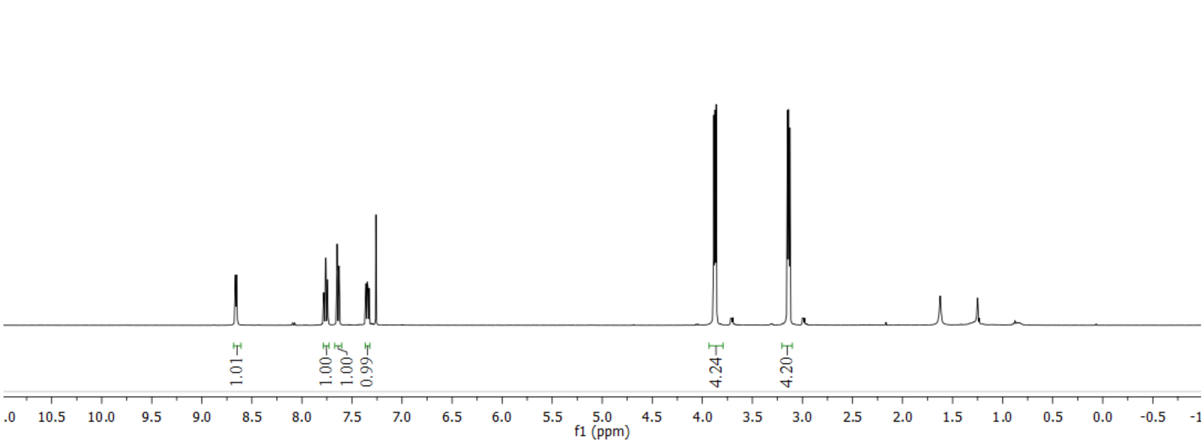

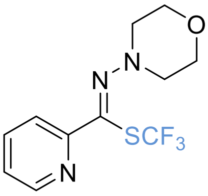

2r

^{1}H NMR (400.2 MHz, $CDCl_3$)

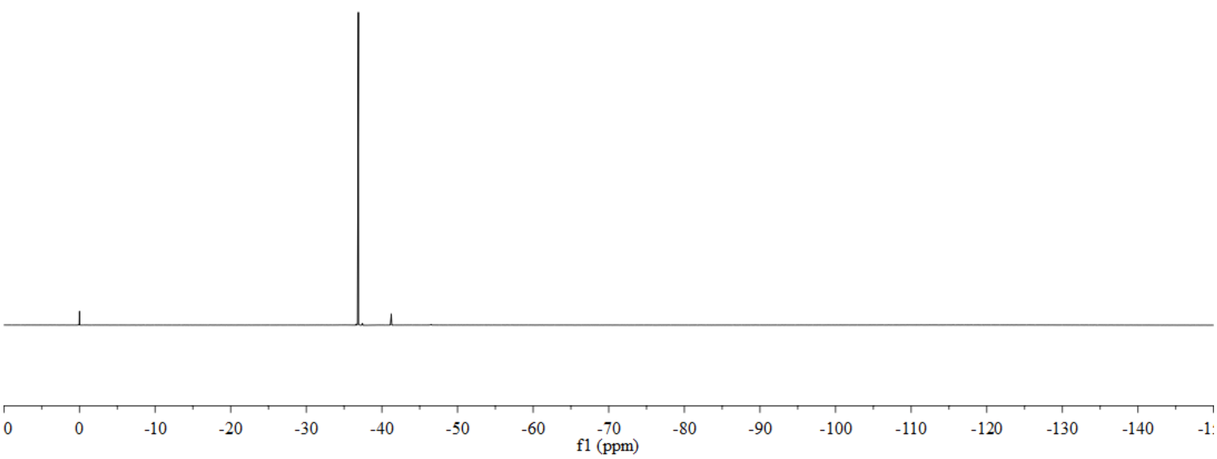

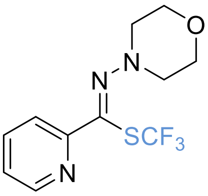

2r

^{19}F NMR (376.6 MHz, $CDCl_3$)

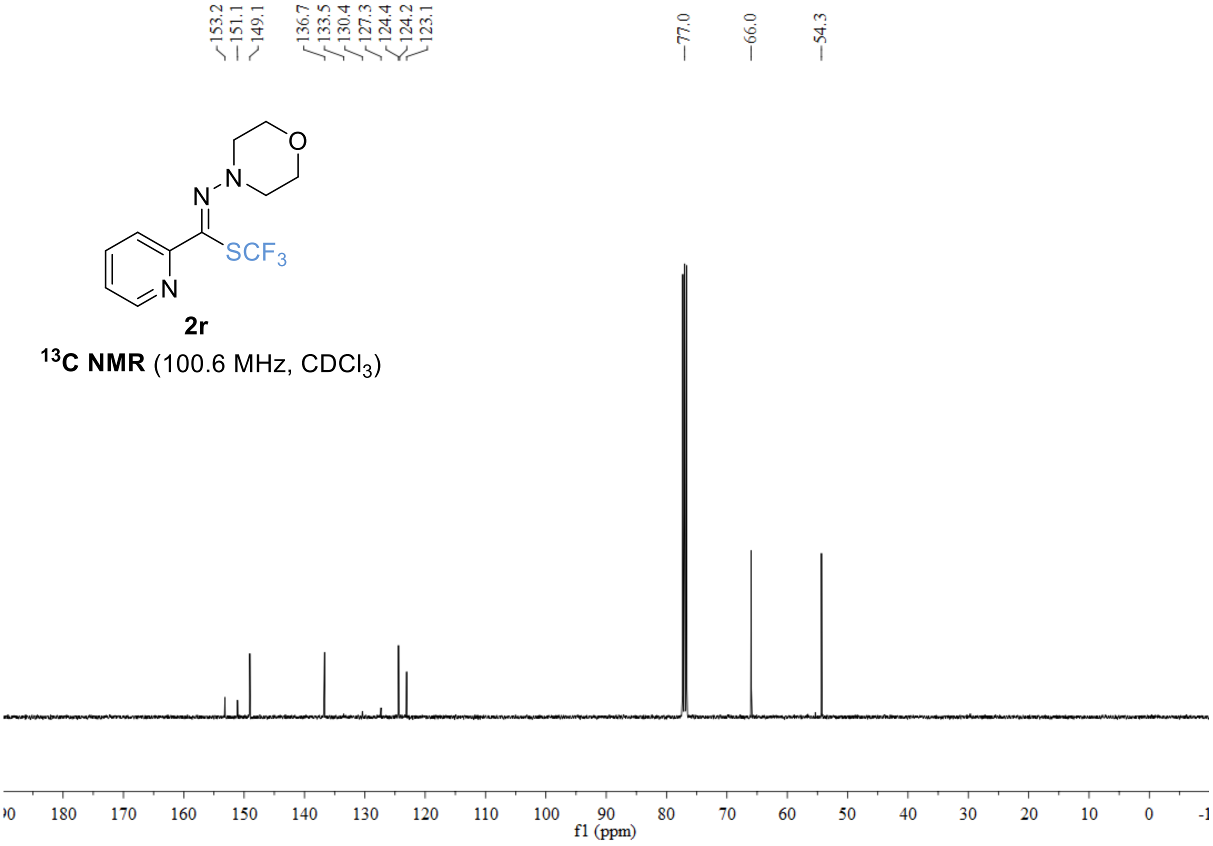

2r

^{13}C NMR (100.6 MHz, $CDCl_3$)

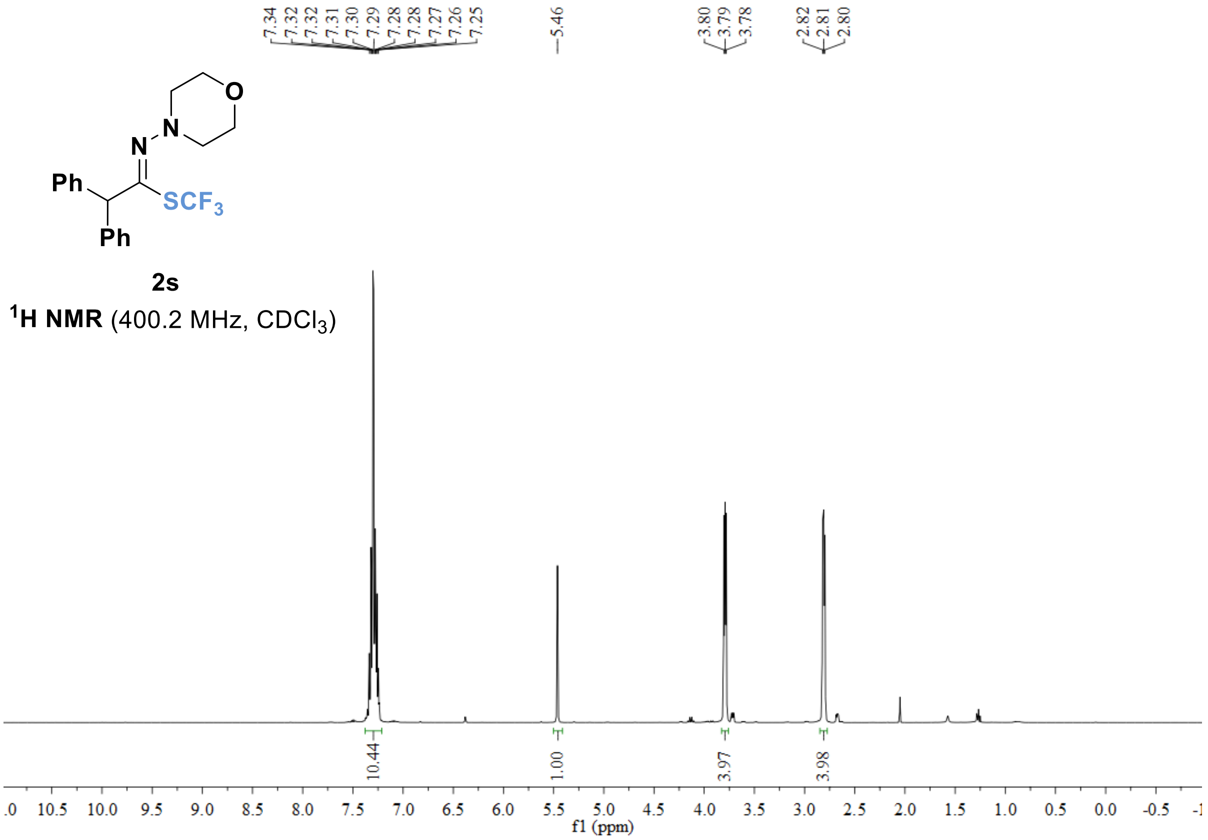

2s

^{1}H NMR (400.2 MHz, $CDCl_3$)

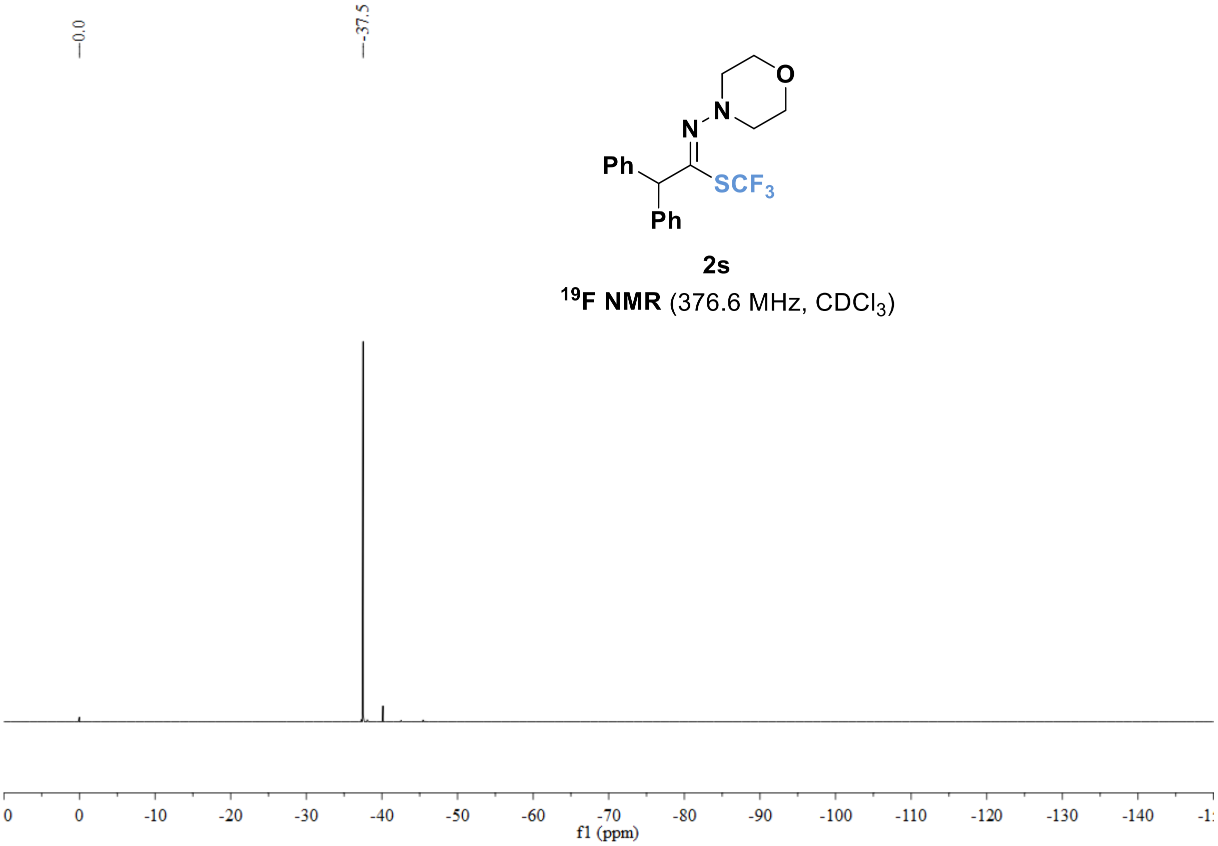

2s

^{19}F NMR (376.6 MHz, $CDCl_3$)

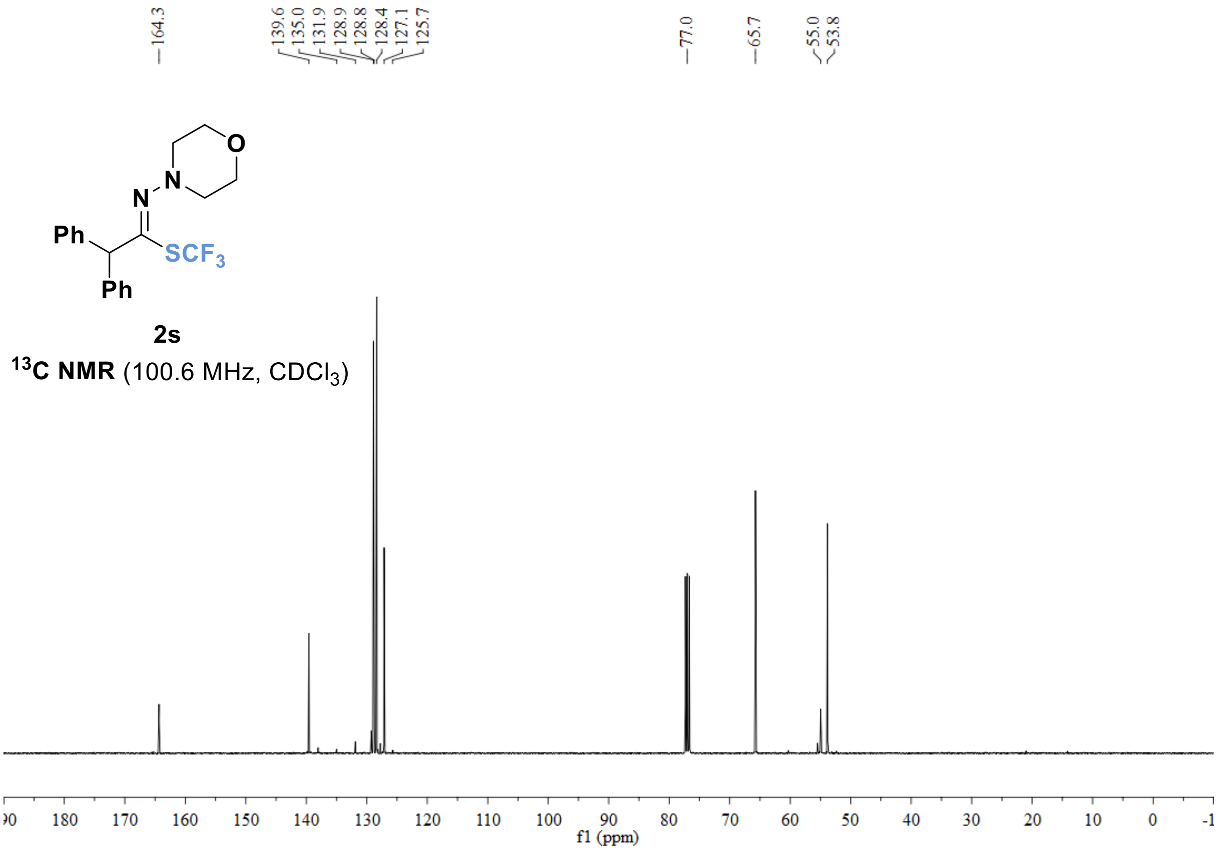

2s

^{13}C NMR (100.6 MHz, $CDCl_3$)

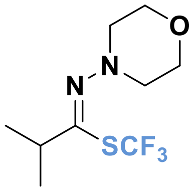

2t

^{1}H NMR (400.2 MHz, $CDCl_3$)

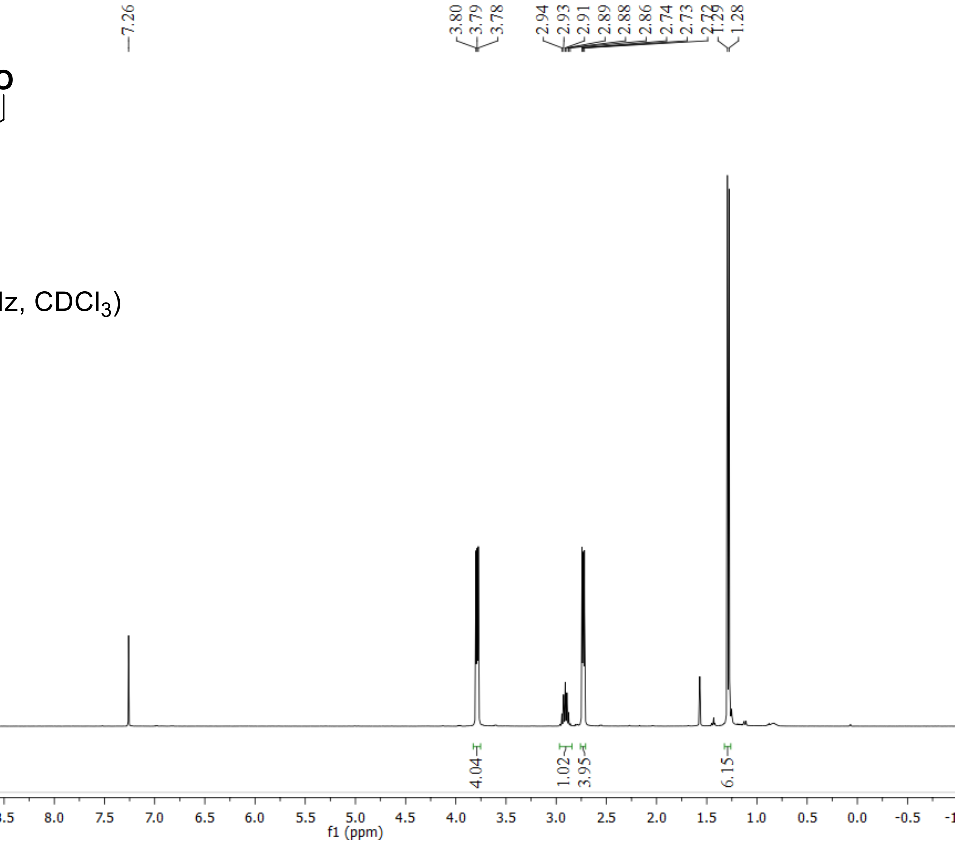

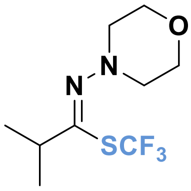

2t

^{19}F NMR (376.6 MHz, $CDCl_3$)

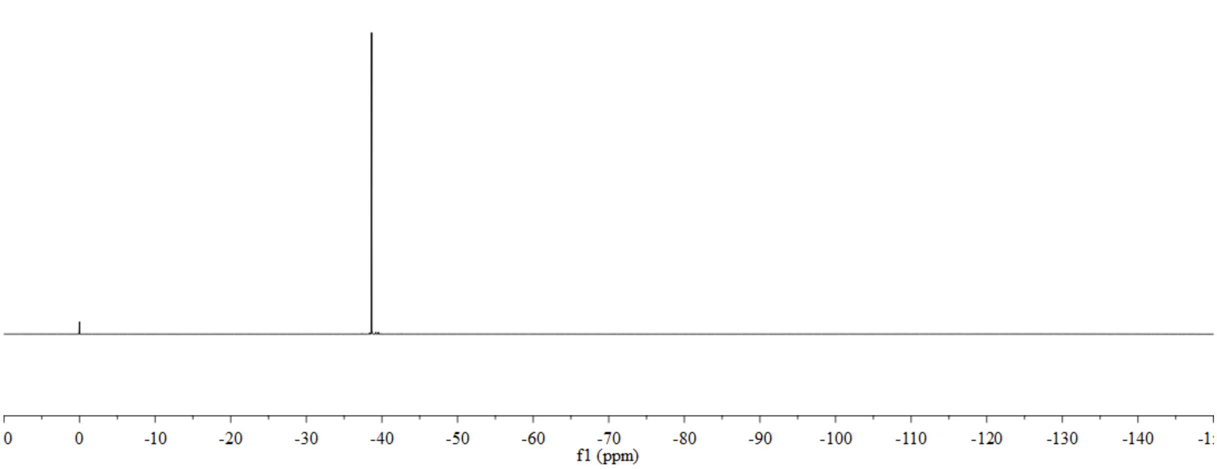

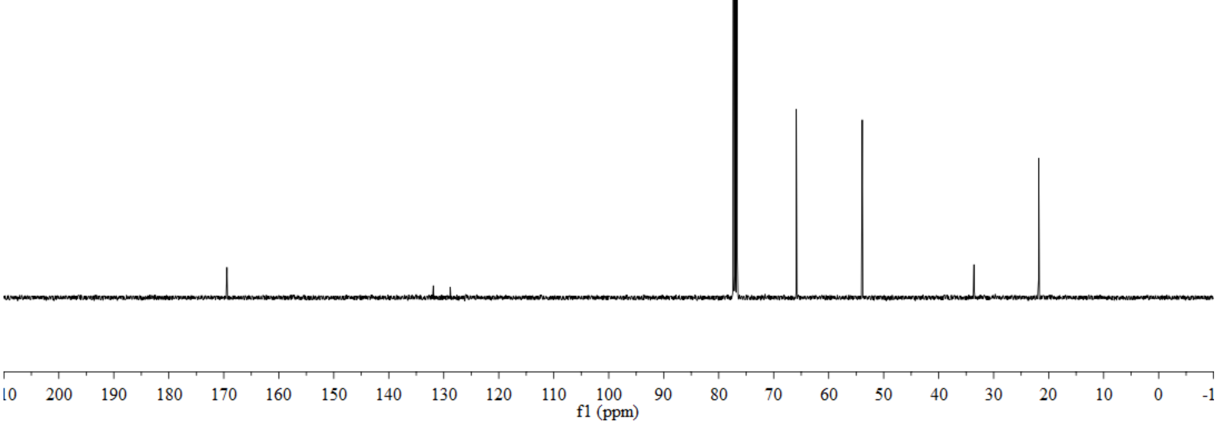

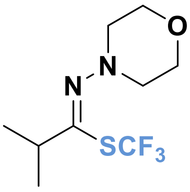

2t

^{13}C NMR (100.6 MHz, $CDCl_3$)

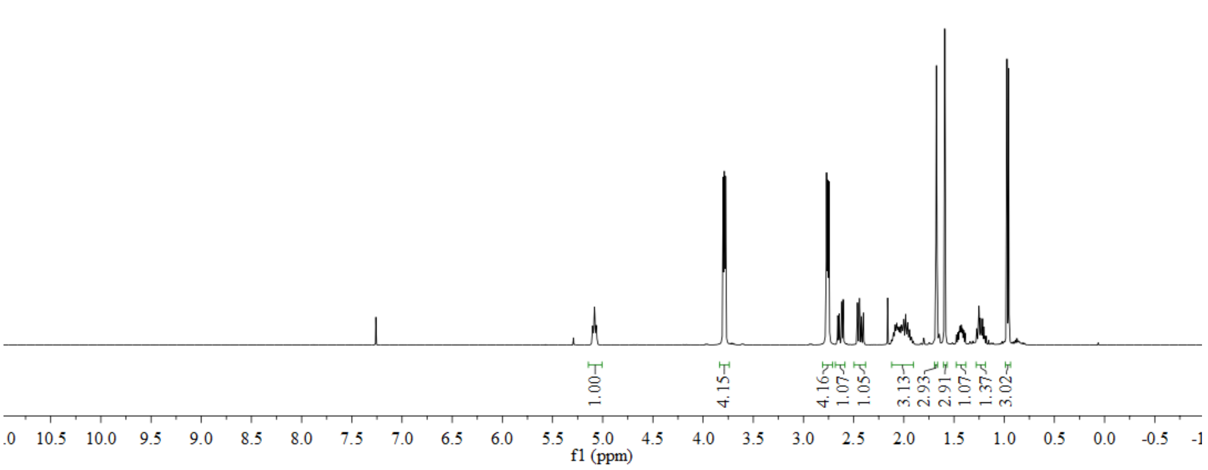

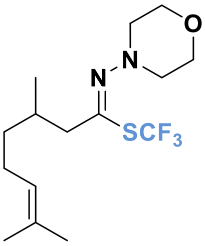

2u

^{1}H NMR (400.2 MHz, $CDCl_3$)

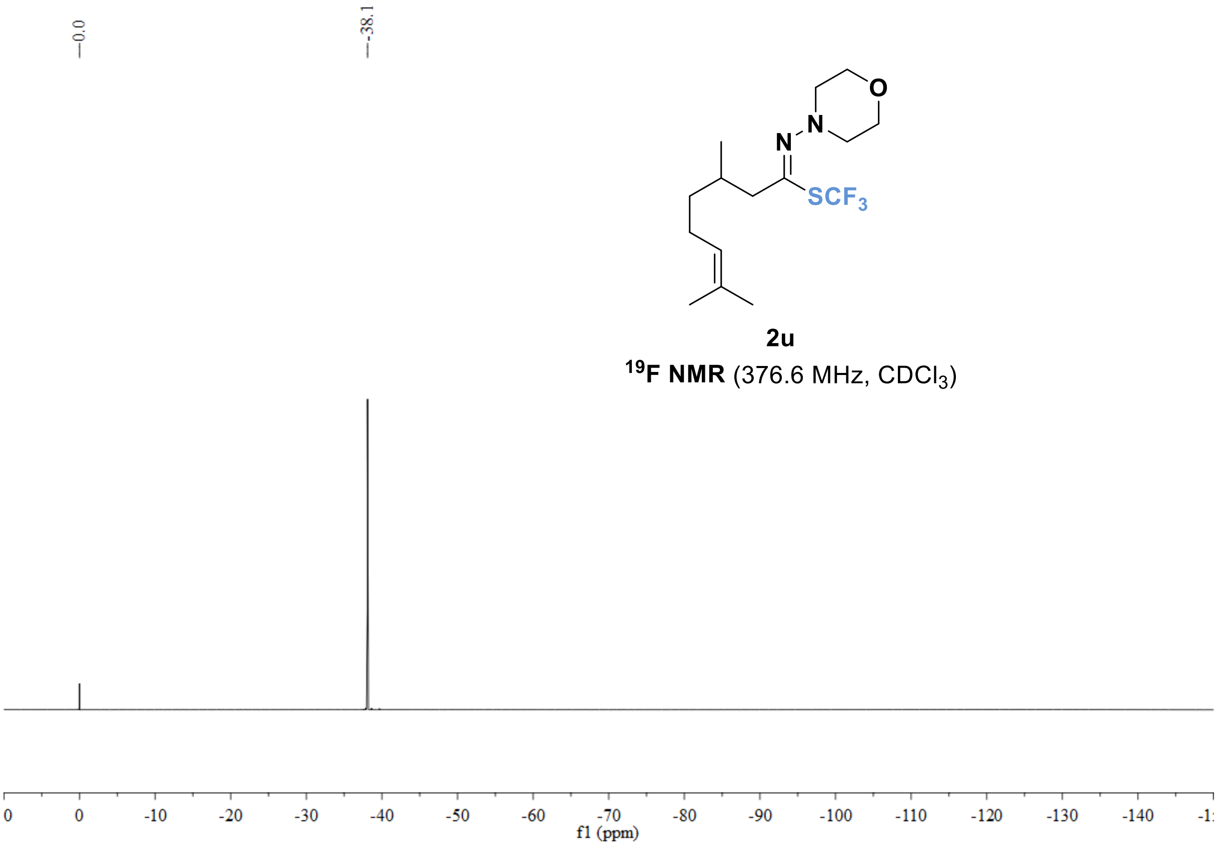

2u
^{19}F NMR (376.6 MHz, $CDCl_3$)

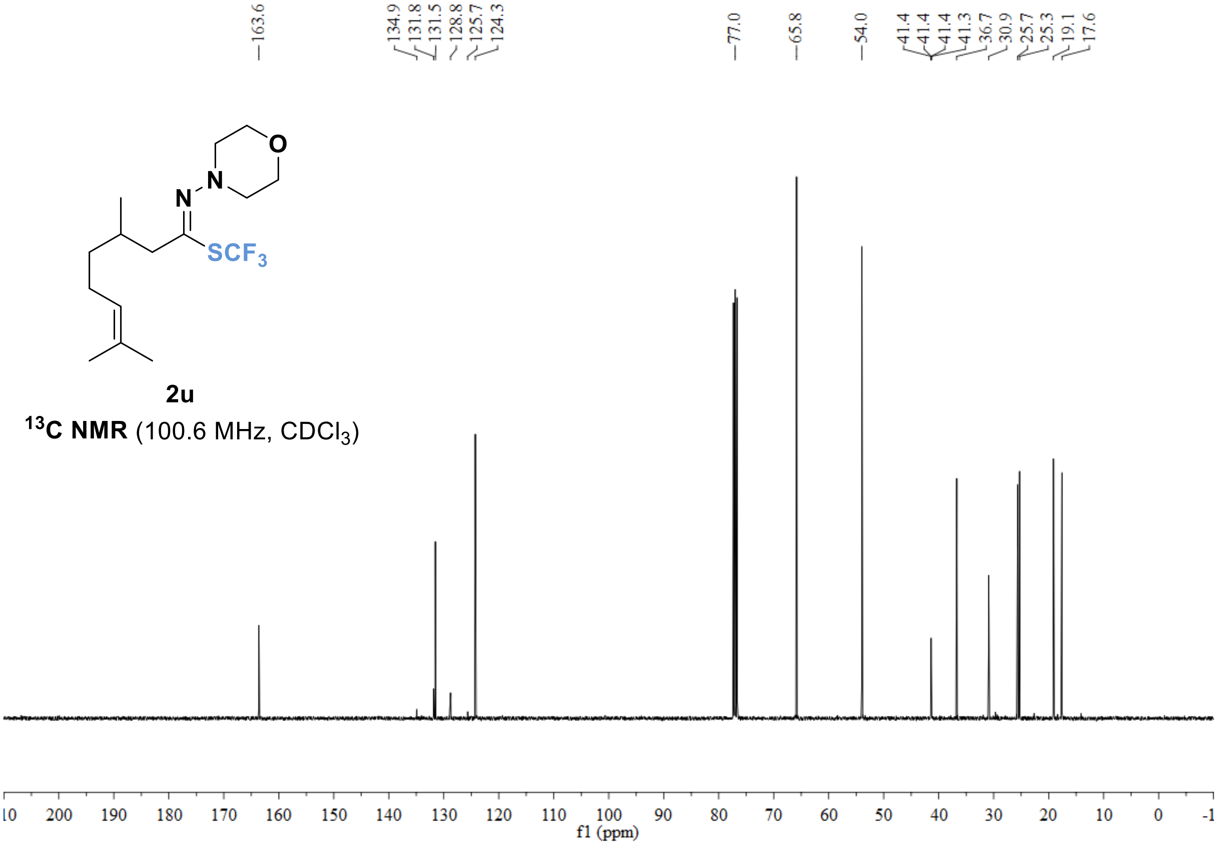

2u
^{13}C NMR (100.6 MHz, $CDCl_3$)

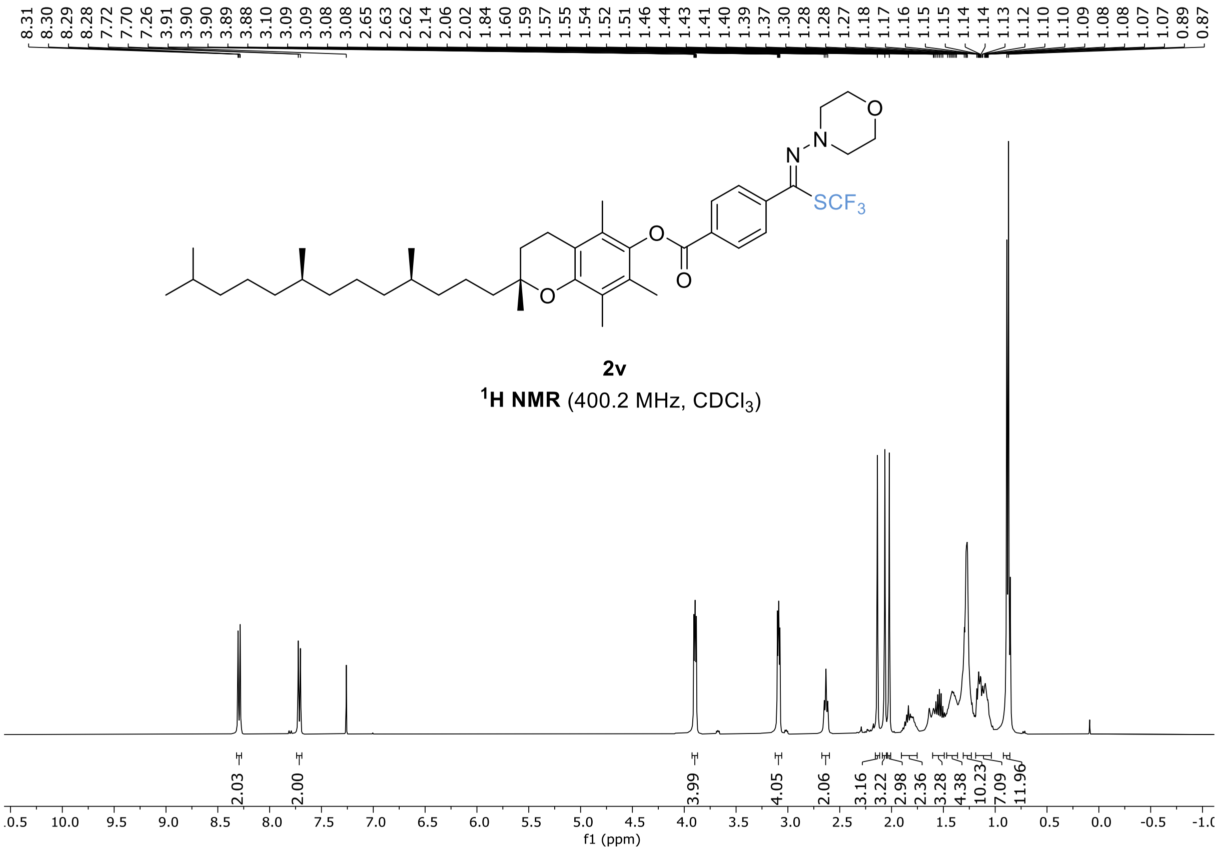
8.31 8.30 8.29 8.28 7.72 7.70 7.26 3.91 3.90 3.90 3.89 3.88 3.10 3.09 3.09 3.08 3.08 2.65 2.63 2.62 2.14 2.06 2.02 1.84 1.60 1.59 1.57 1.55 1.54 1.52 1.51 1.46 1.44 1.43 1.41 1.40 1.39 1.37 1.30 1.28 1.27 1.18 1.17 1.16 1.15 1.15 1.14 1.14 1.13 1.12 1.10 1.10 1.09 1.08 1.07 1.07 0.89 0.87
N
N
O
SCF3
O
O
O
2v
1H NMR (400.2 MHz, CDCl3)
2.03 2.00 3.99 4.05 2.06 3.16 3.22 2.98 2.36 3.28 4.38 10.23 7.09 11.96
f1 (ppm)

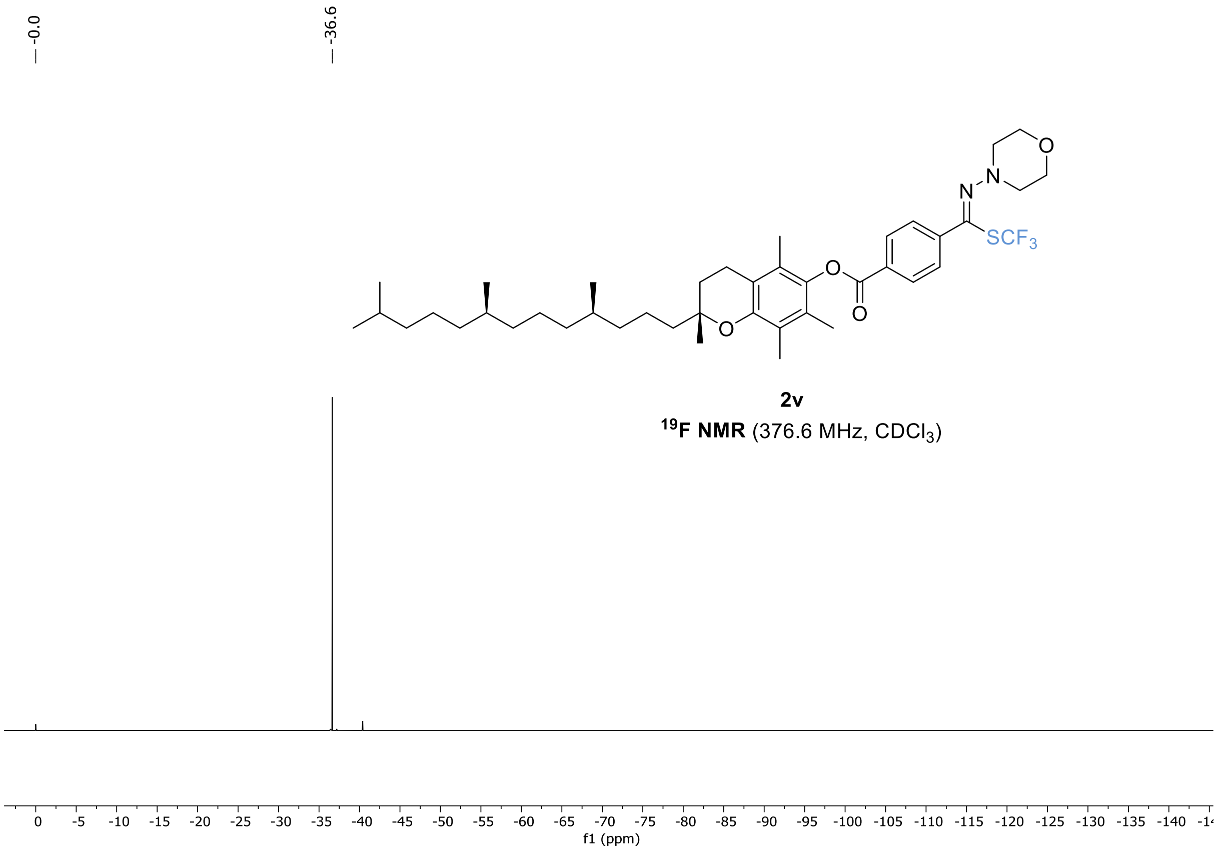
-0.0
-36.6
N
N
O
SCF3
O
O
O
2v
19F NMR (376.6 MHz, CDCl3)
f1 (ppm)

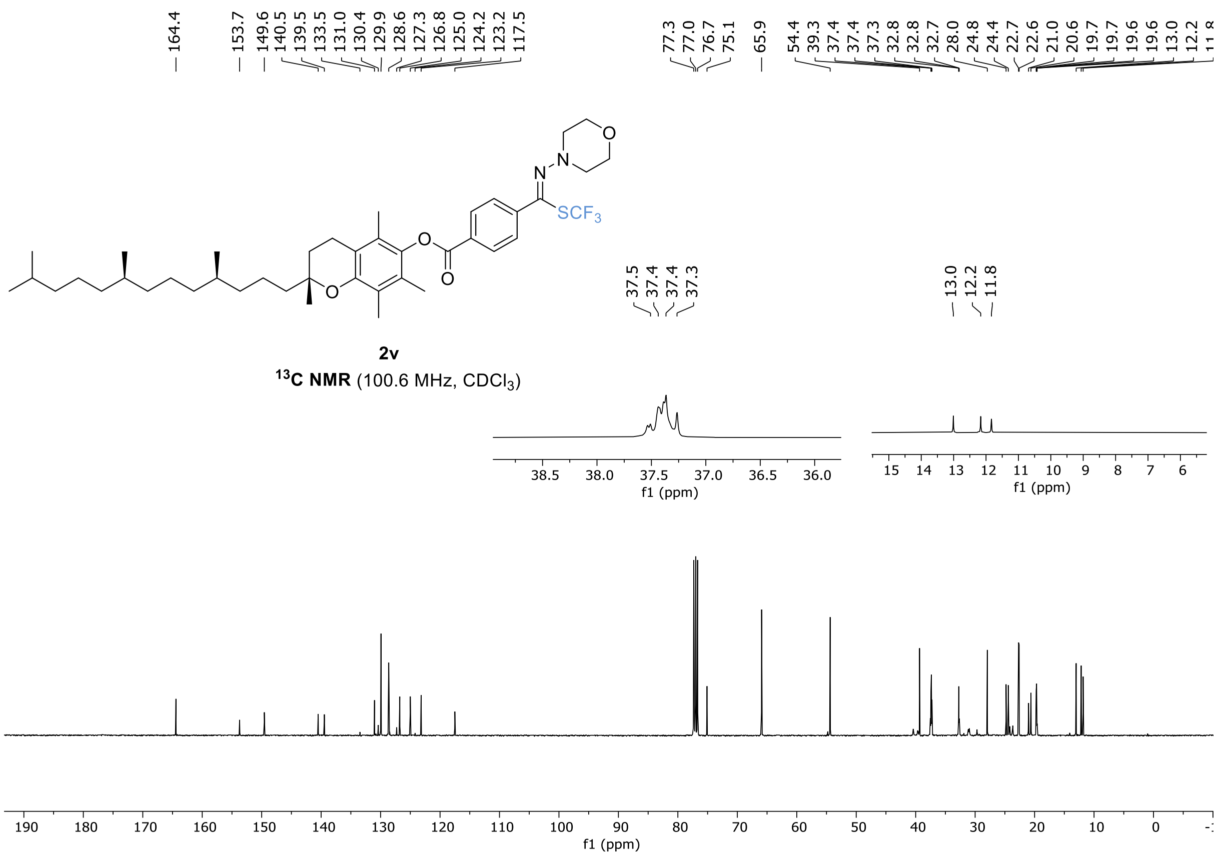

2v

^{13}C NMR (100.6 MHz, $CDCl_3$)

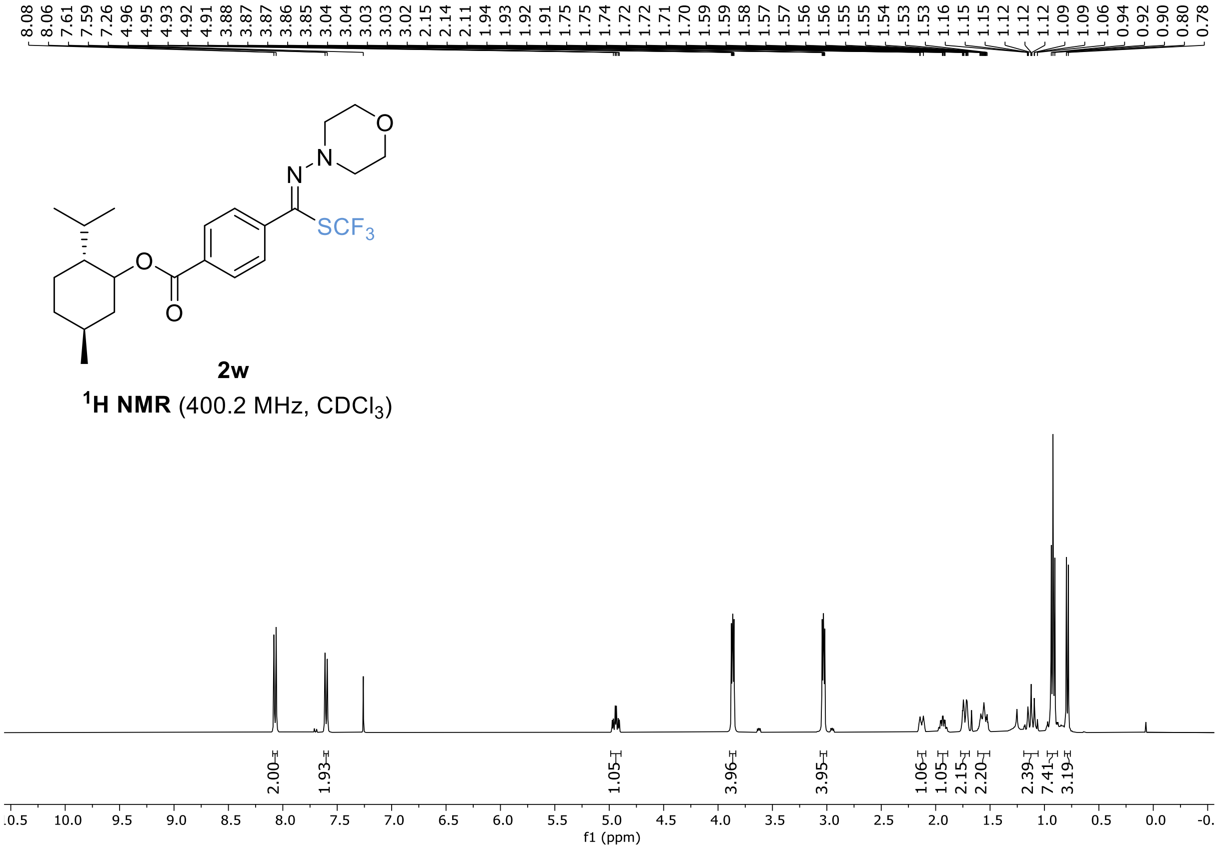

2w

^{1}H NMR (400.2 MHz, $CDCl_3$)

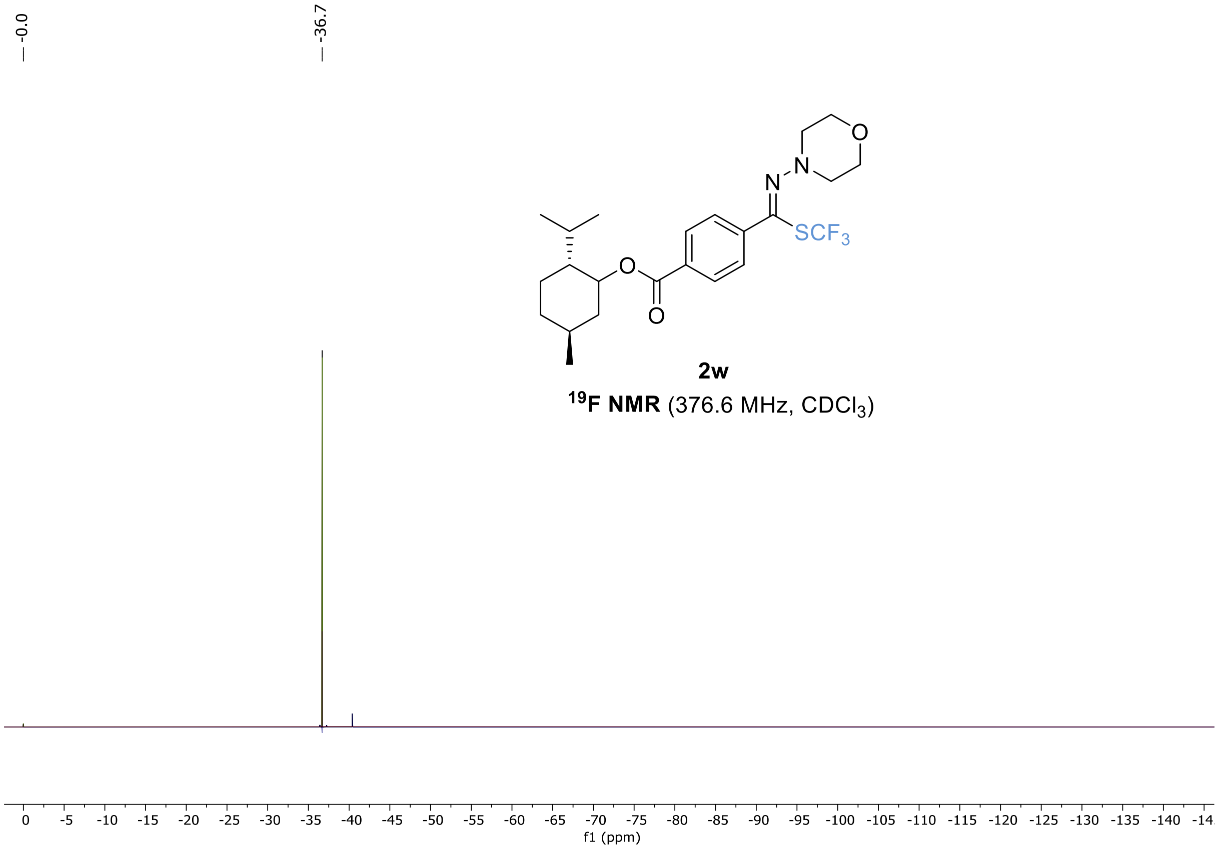

-0.0
-36.7
N
N
O
SCF3
O
O
2w
19F NMR (376.6 MHz, CDCl3)
0 -5 -10 -15 -20 -25 -30 -35 -40 -45 -50 -55 -60 -65 -70 -75 -80 -85 -90 -95 -100 -105 -110 -115 -120 -125 -130 -135 -140
f1 (ppm)

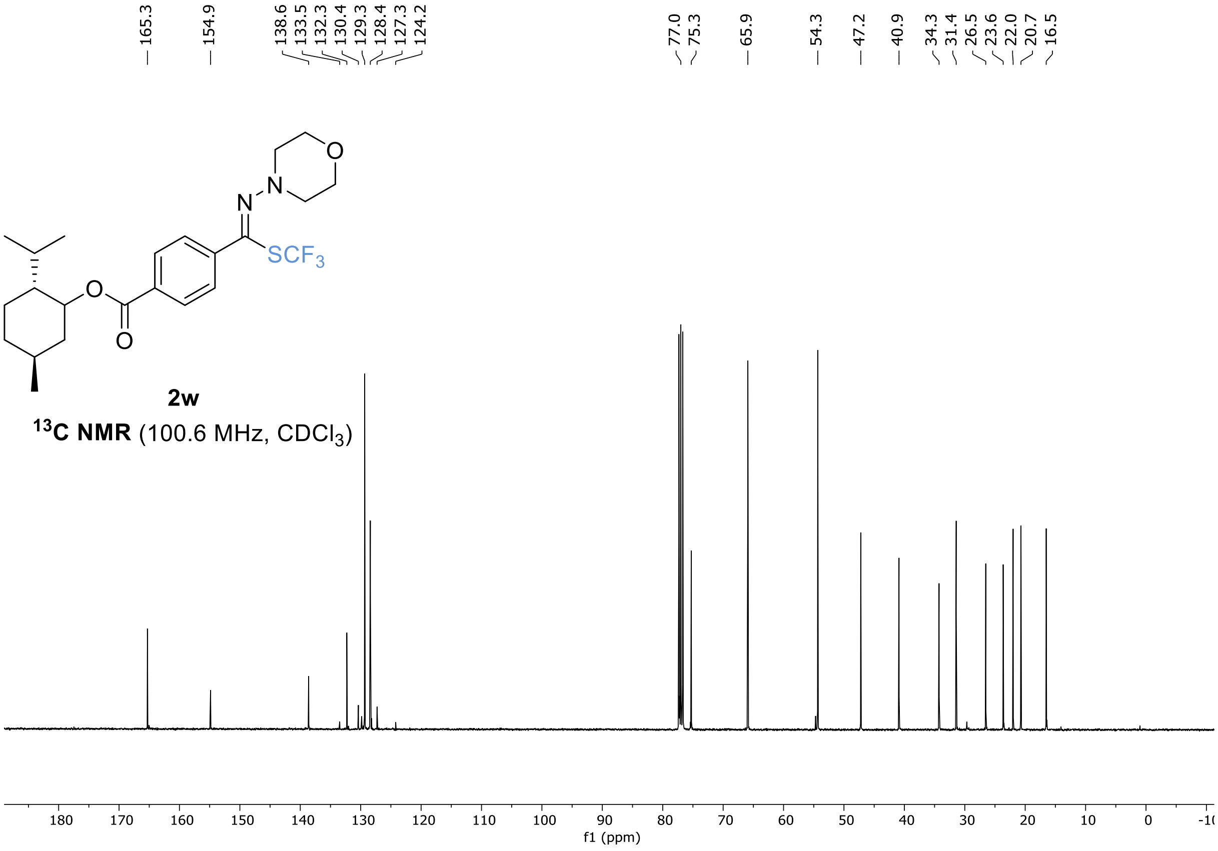

165.3
154.9
138.6
133.5
132.3
130.4
129.3
128.4
127.3
124.2
77.0
75.3
65.9
54.3
47.2
40.9
34.3
31.4
26.5
23.6
22.0
20.7
16.5
N
N
O
SCF3
O
O
2w
13C NMR (100.6 MHz, CDCl3)
180 170 160 150 140 130 120 110 100 90 80 70 60 50 40 30 20 10 0
f1 (ppm)

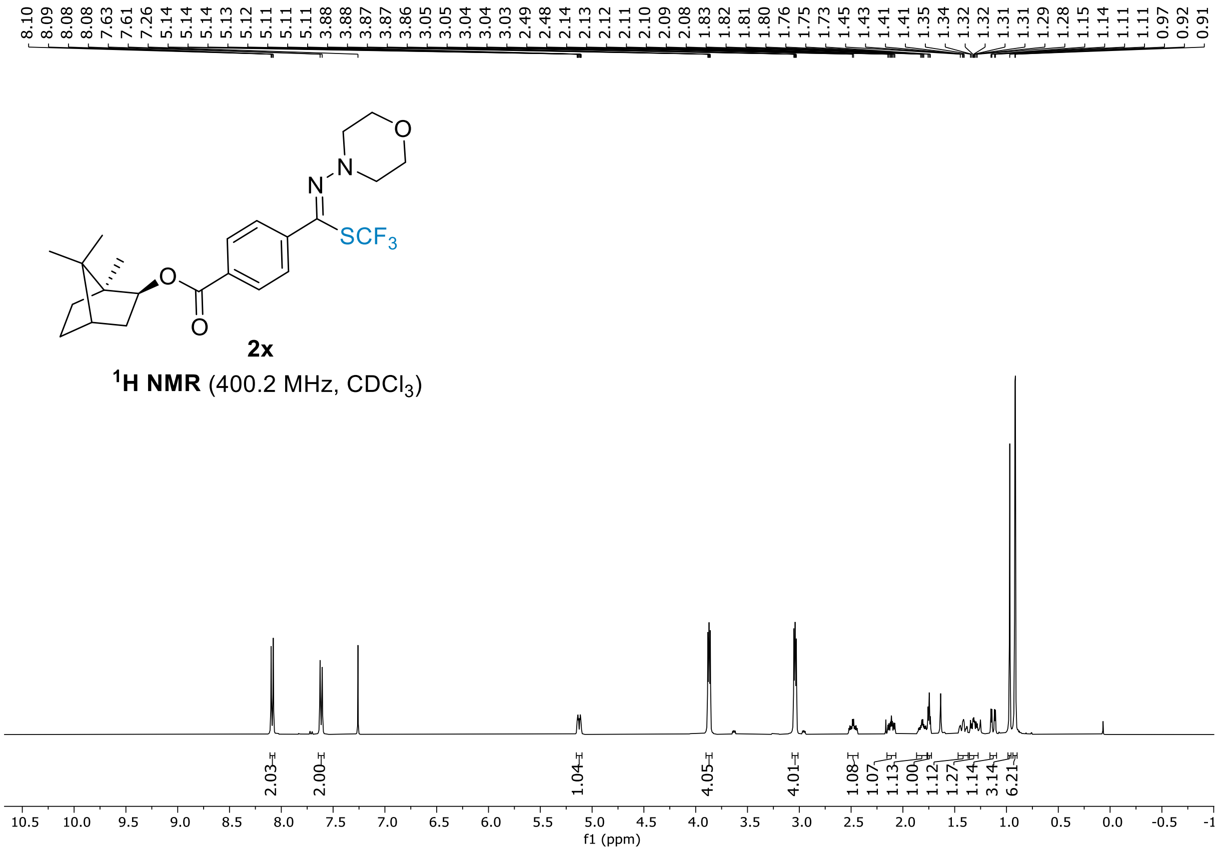
8.10 8.09 8.08 8.08 7.63 7.61 7.26 5.14 5.14 5.14 5.13 5.12 5.11 5.11 5.11 3.88 3.88 3.87 3.87 3.86 3.05 3.05 3.04 3.04 3.03 2.49 2.48 2.14 2.13 2.12 2.11 2.10 2.09 2.08 1.83 1.82 1.81 1.80 1.76 1.75 1.73 1.45 1.43 1.41 1.41 1.35 1.34 1.32 1.32 1.31 1.31 1.29 1.28 1.15 1.14 1.11 1.11 0.97 0.92 0.91
SCF3
2x
1H NMR (400.2 MHz, CDCl3)
2.03 2.00 1.04 4.05 4.01 1.08 1.07 1.13 1.00 1.12 1.27 1.14 3.14 6.21
f1 (ppm)

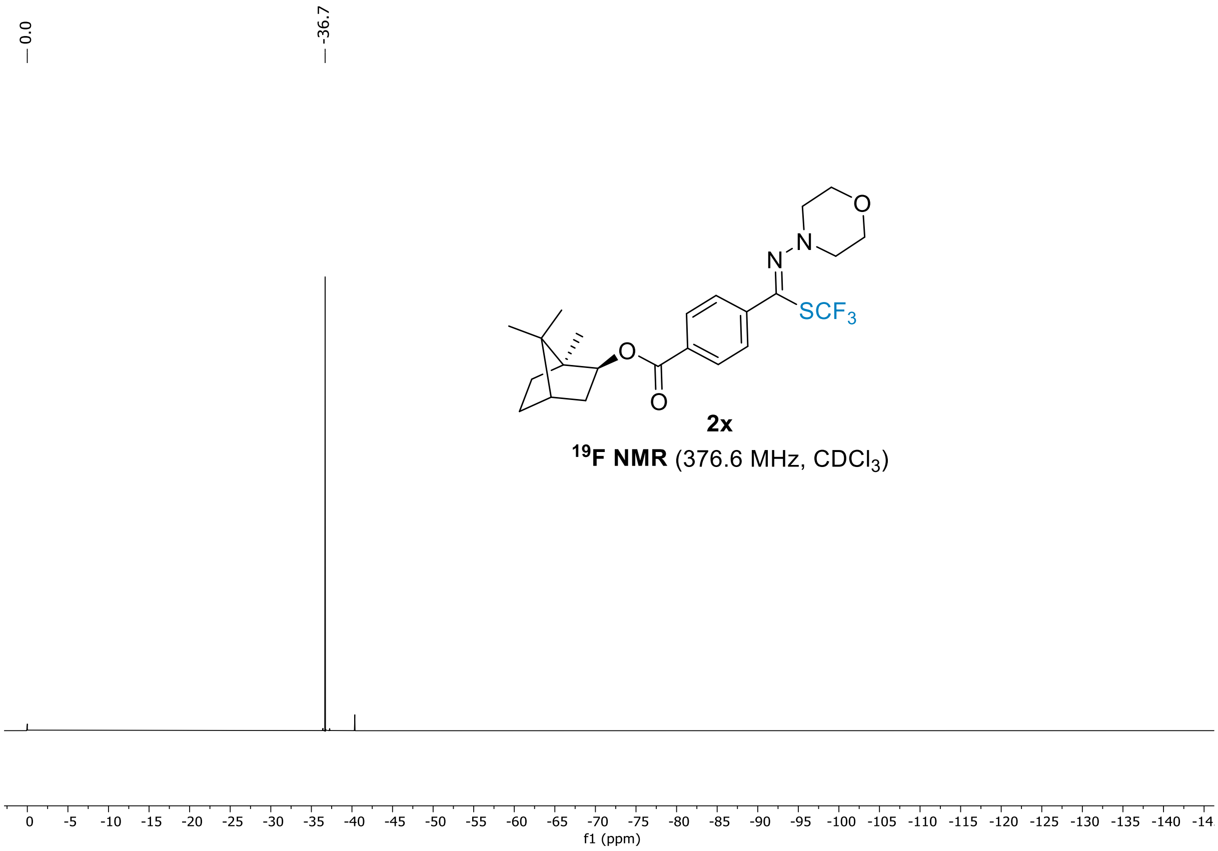
0.0
-36.7
SCF3
2x
19F NMR (376.6 MHz, CDCl3)
f1 (ppm)

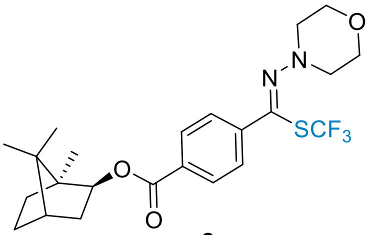

2x

^{13}C NMR (100.6 MHz, $CDCl_3$)

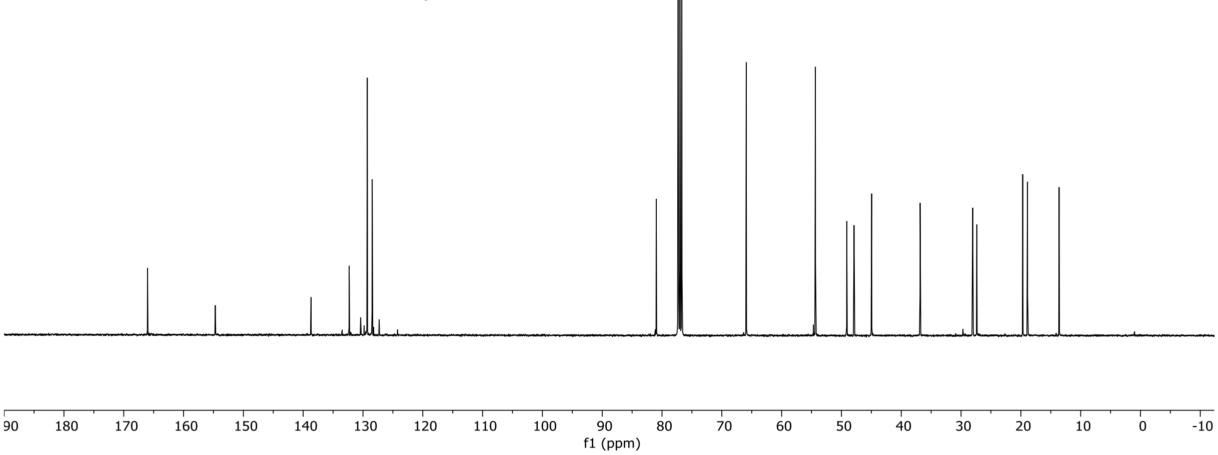

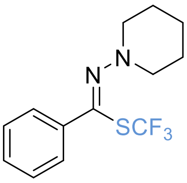

3a

^{1}H NMR (400.2 MHz, $CDCl_3$)

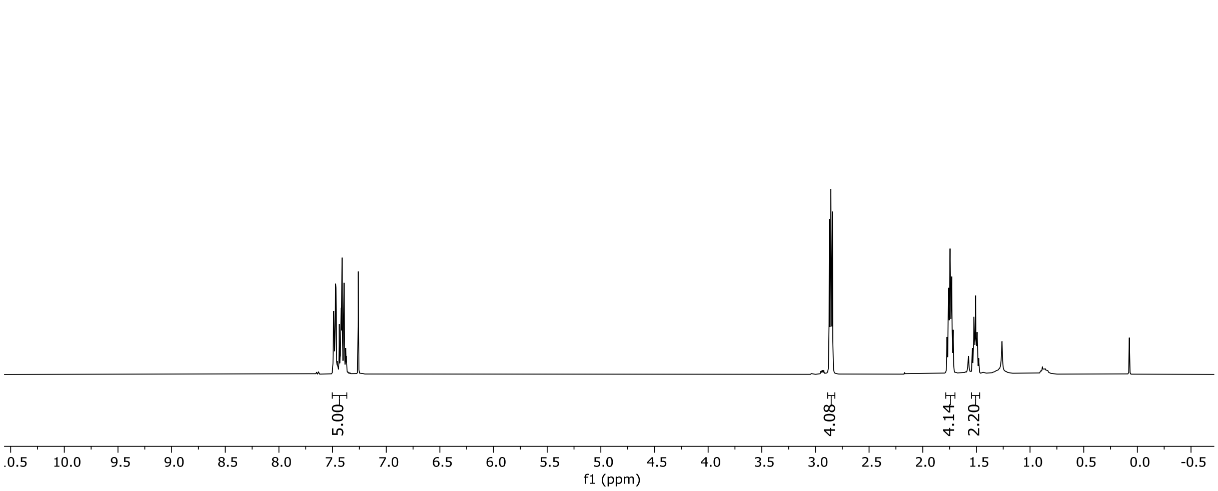

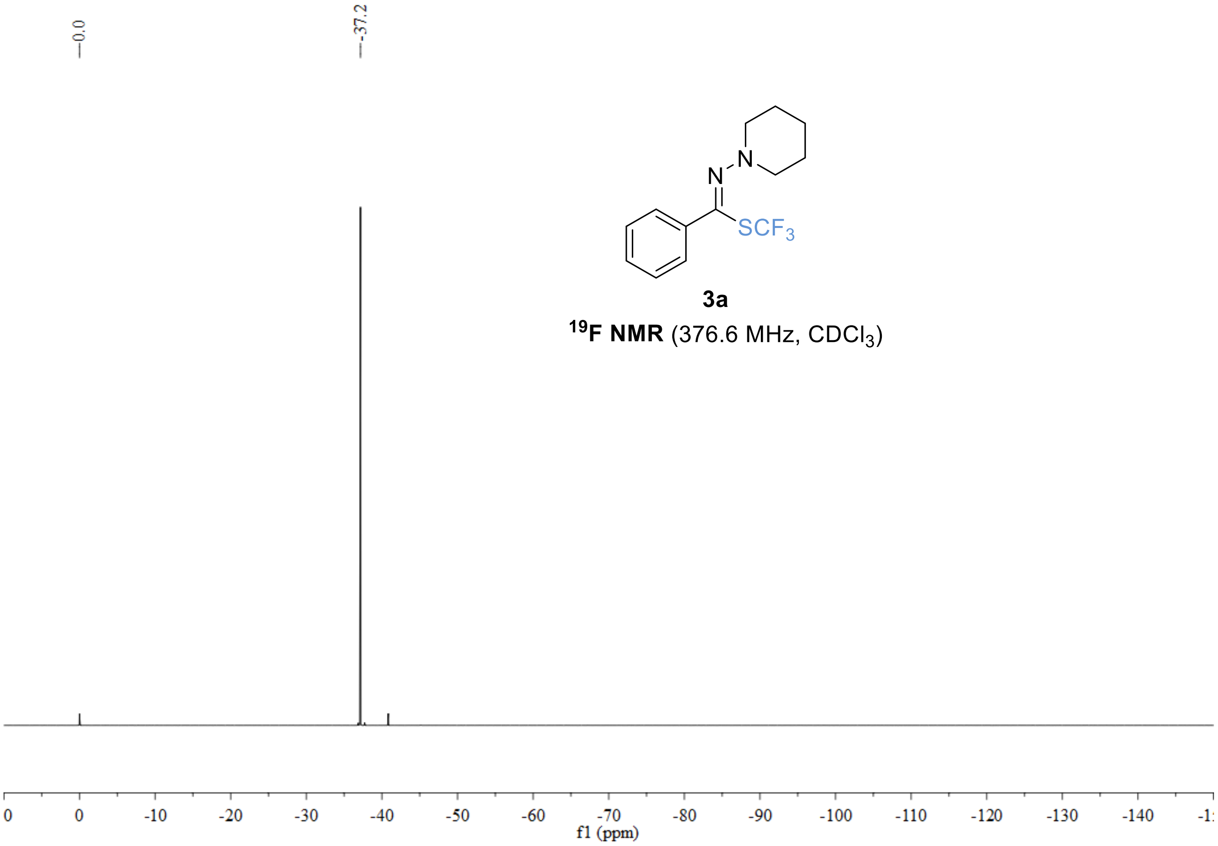

0.0
-37.2
SCF3
3a
19F NMR (376.6 MHz, CDCl3)
f1 (ppm)

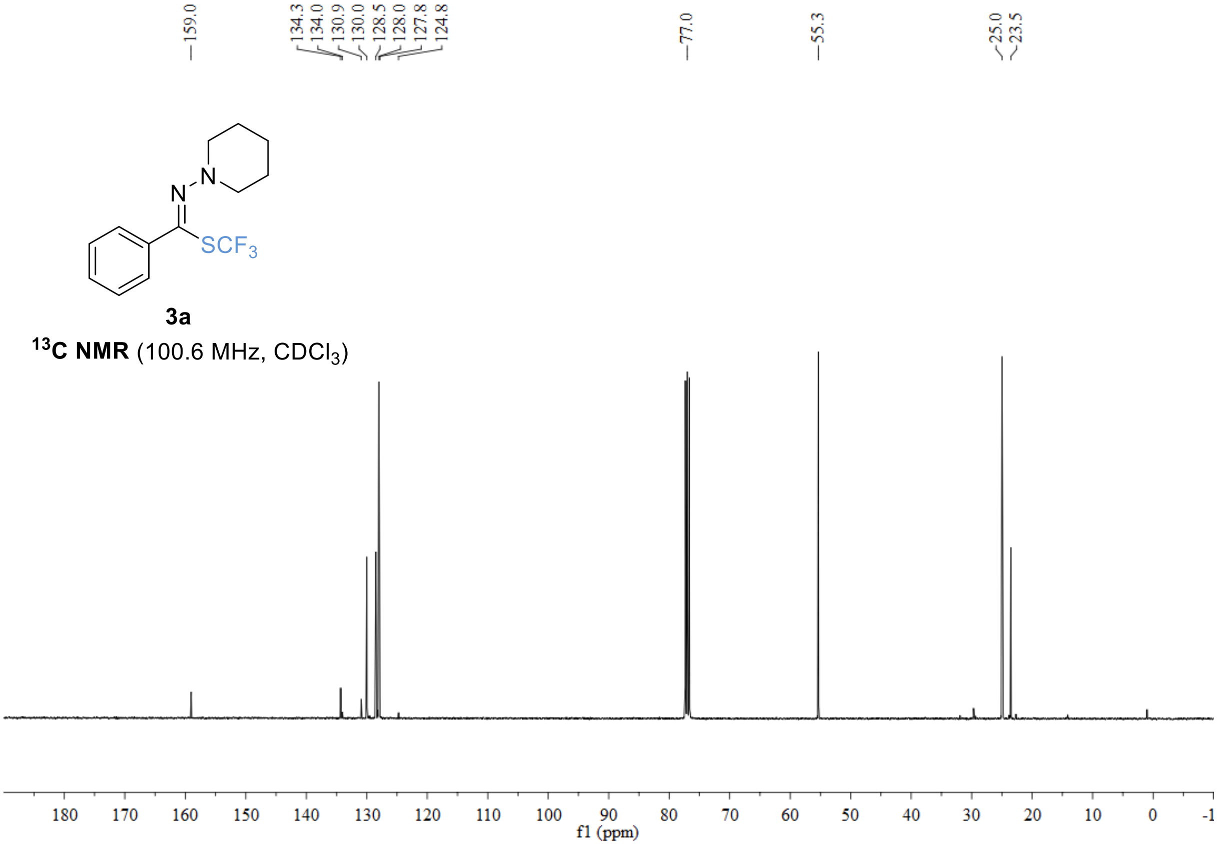

159.0
134.3
134.0
130.9
130.0
128.5
128.0
127.8
124.8
77.0
55.3
25.0
23.5
SCF3
3a
13C NMR (100.6 MHz, CDCl3)
f1 (ppm)

4a

^{1}**H NMR** (400.2 MHz, $CDCl_3$)

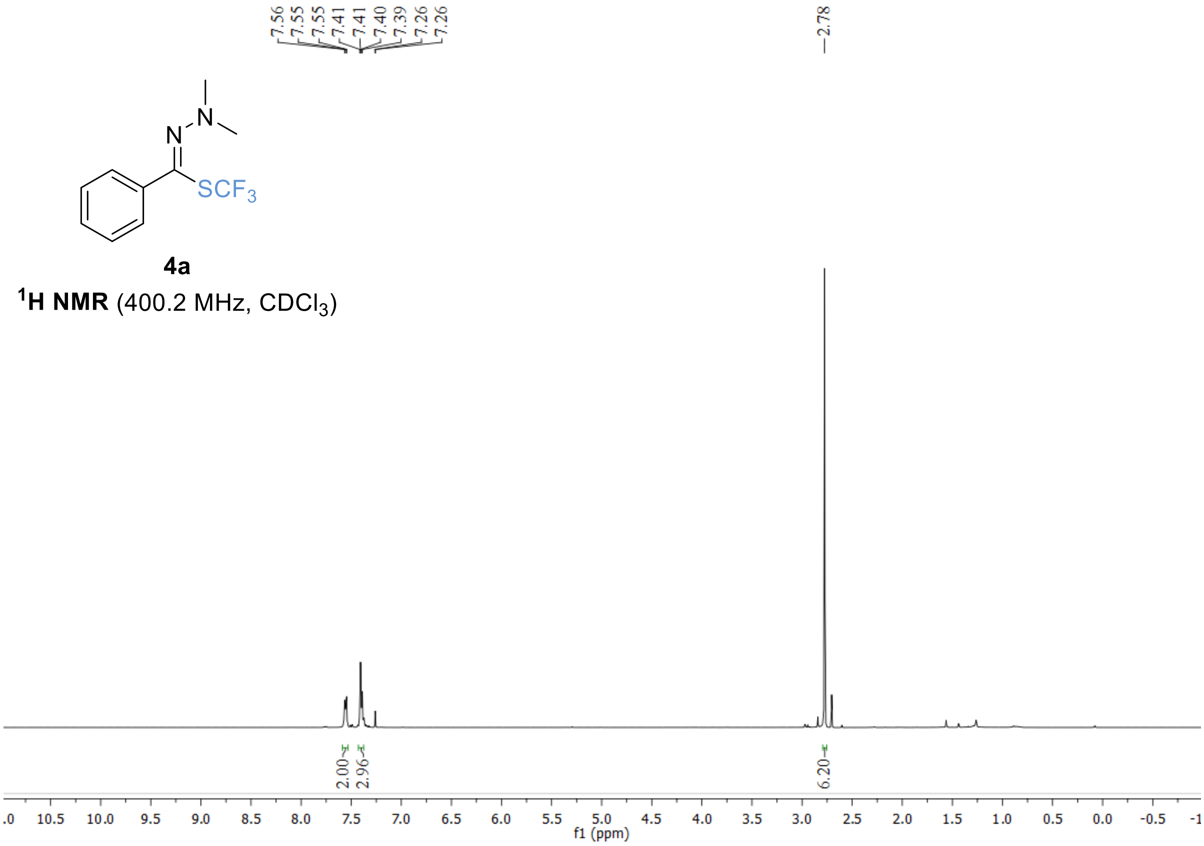

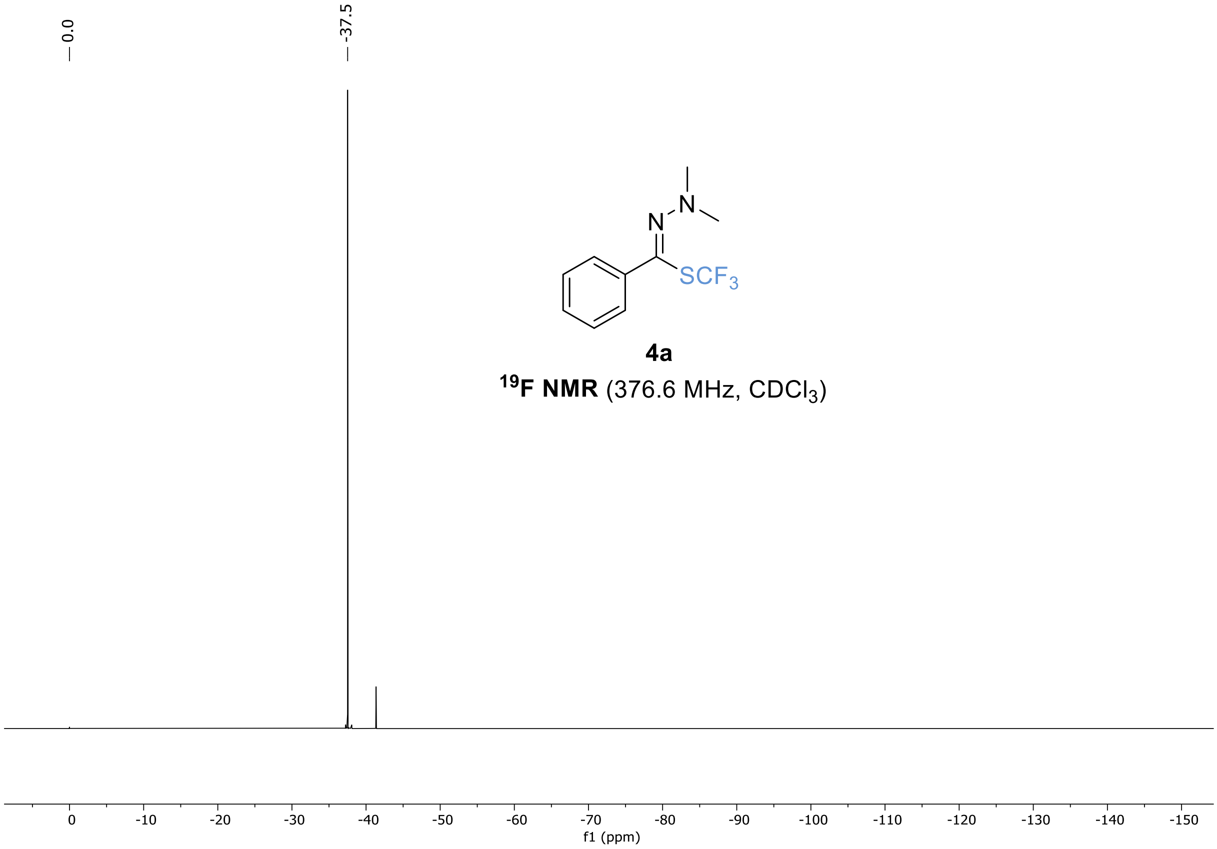

4a

^{19}**F NMR** (376.6 MHz, $CDCl_3$)

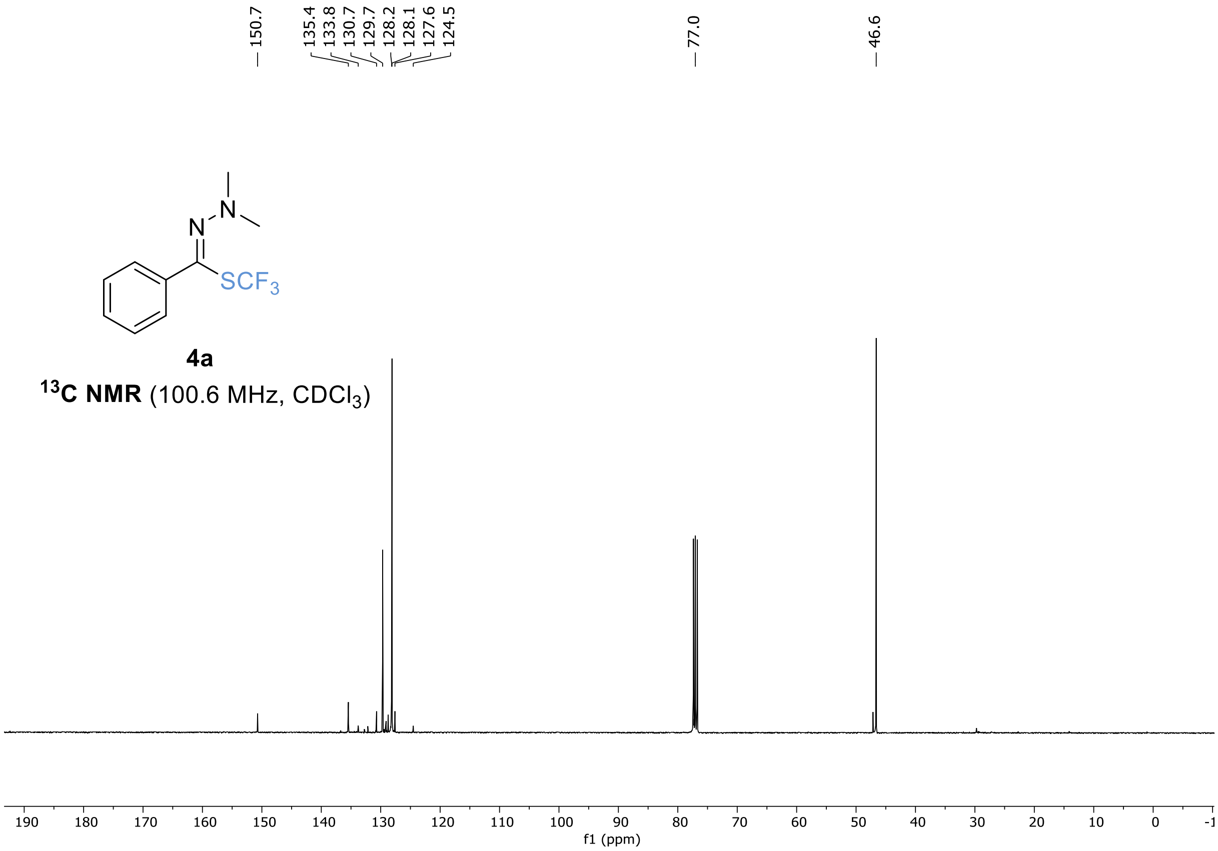

4a

^{13}C NMR (100.6 MHz, $CDCl_3$)

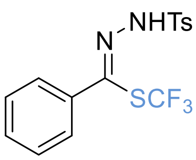

5a

^{1}H NMR (400.2 MHz, $CDCl_3$)

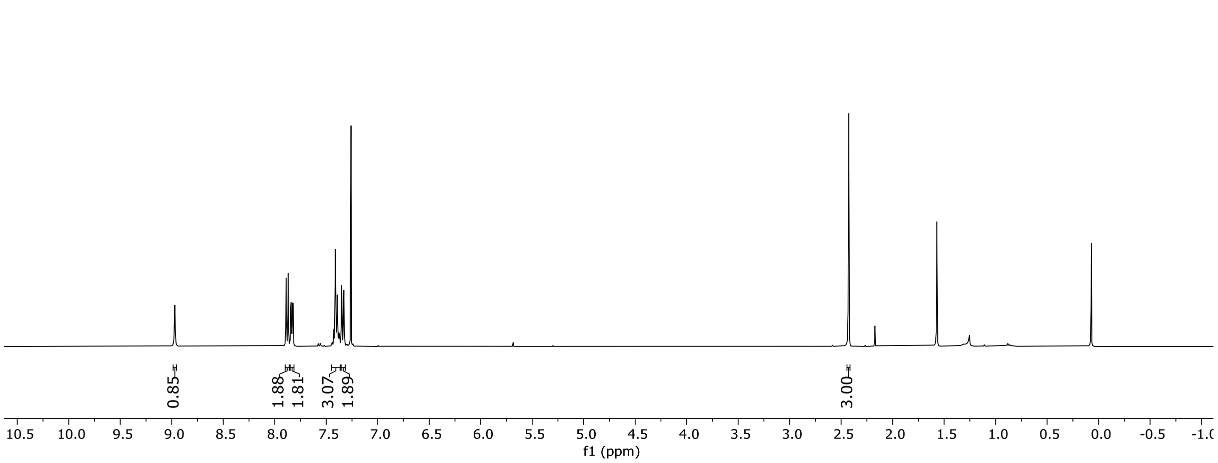

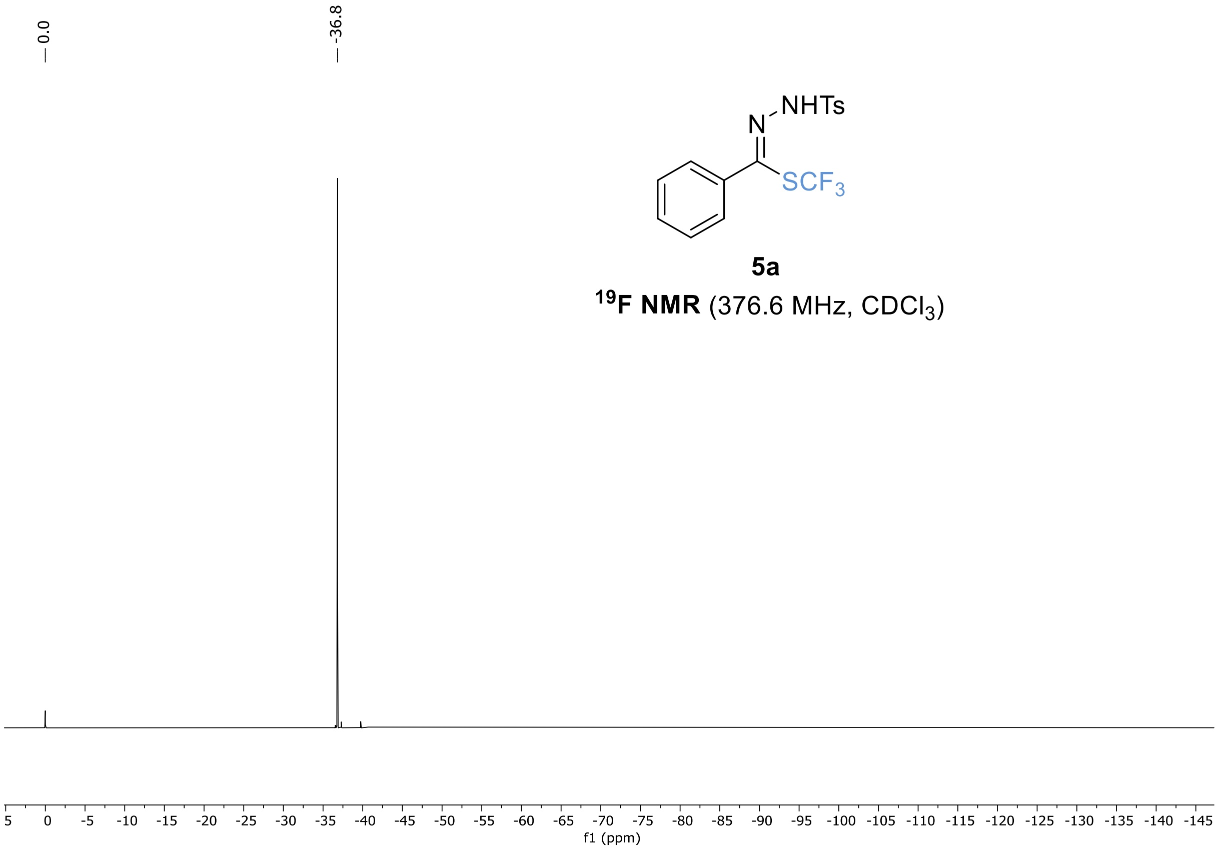

5a

^{19}F NMR (376.6 MHz, $CDCl_3$)

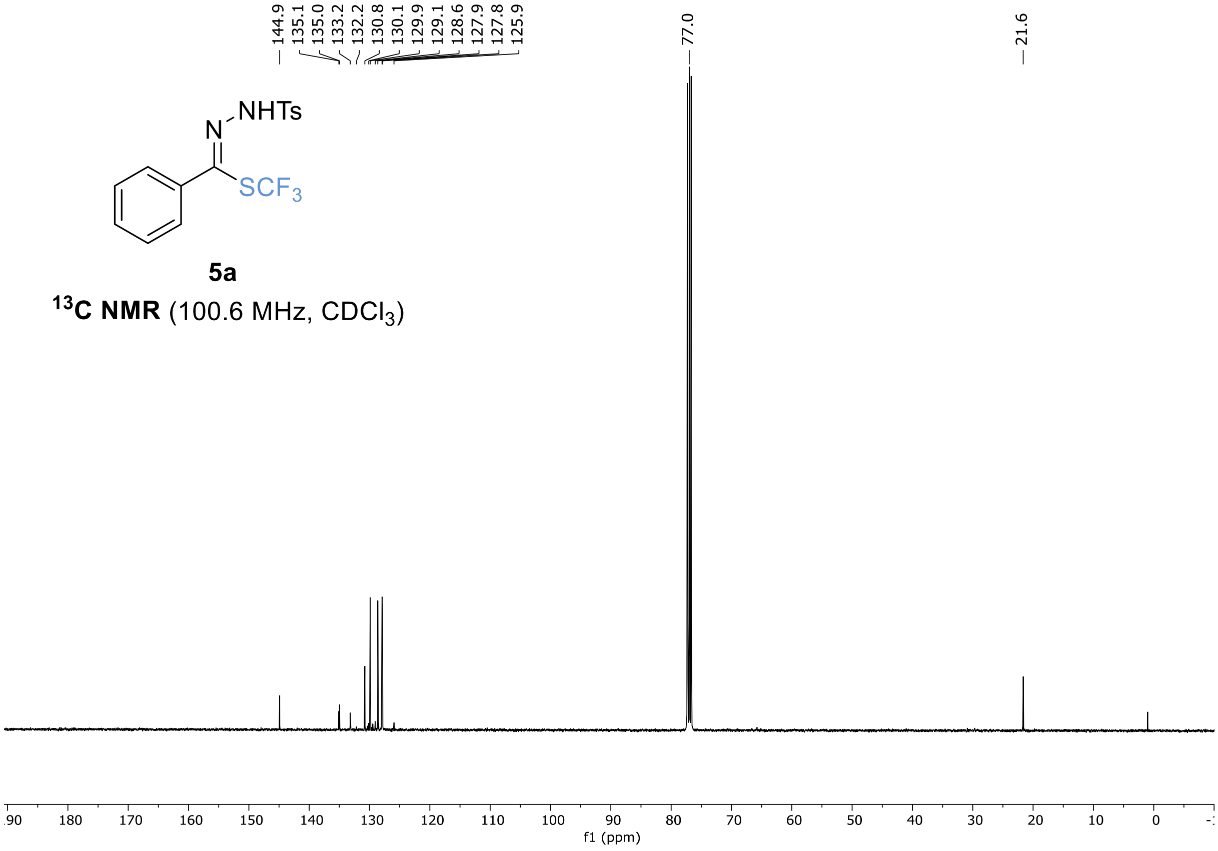

5a

^{13}C NMR (100.6 MHz, $CDCl_3$)

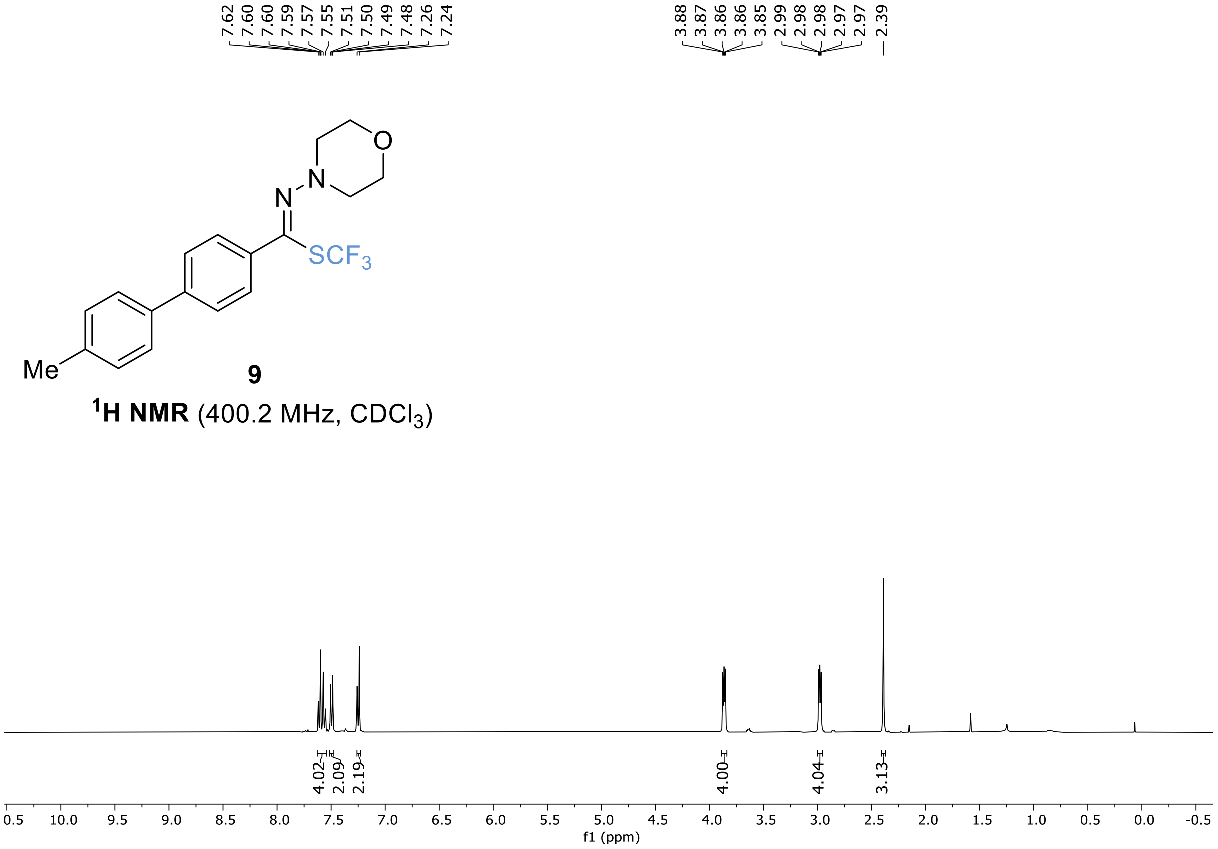
7.62
7.60
7.60
7.59
7.57
7.55
7.51
7.50
7.49
7.48
7.26
7.24
3.88
3.87
3.86
3.86
3.85
2.99
2.98
2.98
2.97
2.97
2.39
N
N
O
SCF3
Me
9
1H NMR (400.2 MHz, CDCl3)
4.02
2.09
2.19
4.00
4.04
3.13
f1 (ppm)

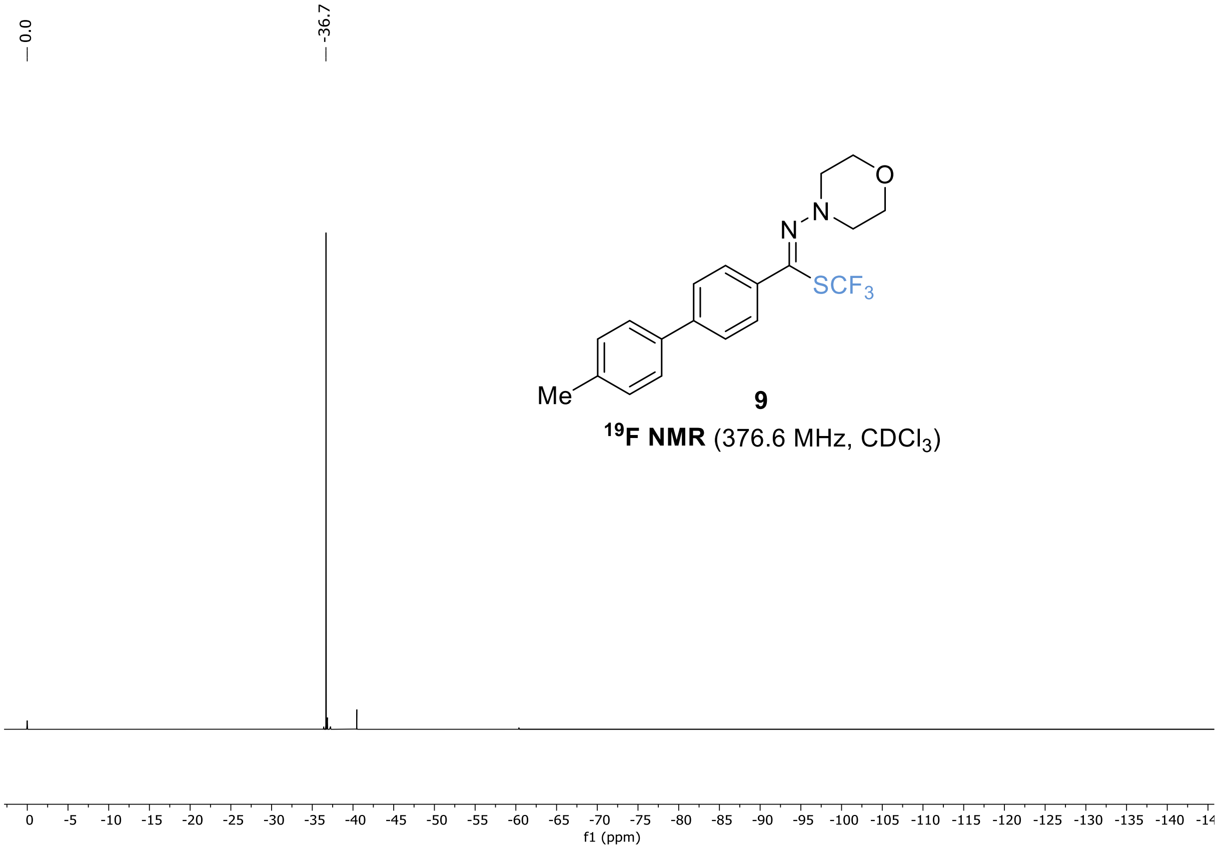
0.0
-36.7
N
N
O
SCF3
Me
9
19F NMR (376.6 MHz, CDCl3)
f1 (ppm)

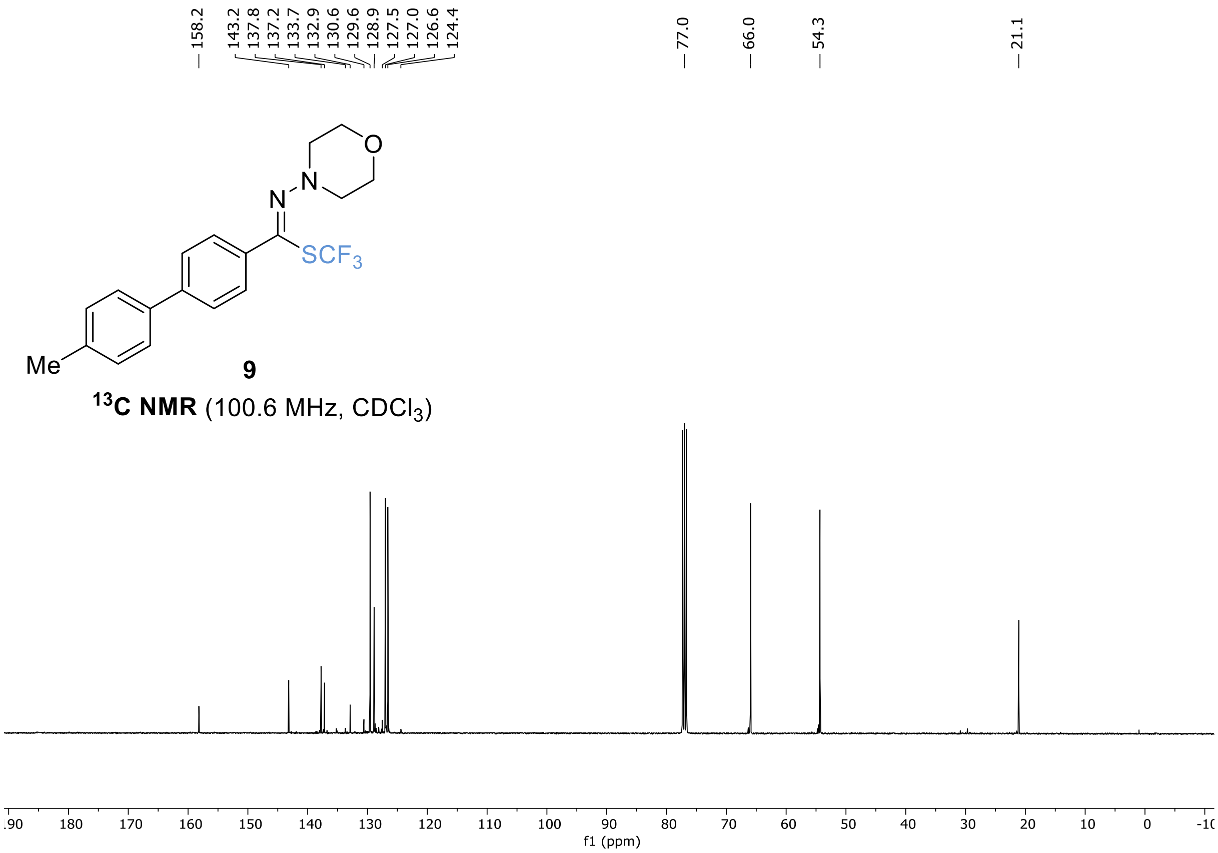

^{13}C NMR (100.6 MHz, $CDCl_3$)

12. References

[1] Xiong, H.-Y.; Pannecoucke, X.; Besset, T. *Org. Chem. Front*., **2016**, *3*, 620–624.

[2] Dauben, H.; McCoy, L. *J. Am. Chem. Soc.*, **1959**, *81*, 4863–4873.

[3] Kumar Ghosh, A.; Mondal, S.; Hajra, A. *Org. Lett.,* **2020***, 22,* 2771–2775.

[4] Dubrovskiy, A.; Larock, R. *Org Lett.,* **2011**, *13*, 4136–4139.

[5] Mishra, M.; Maharana, P. K.; Karjee, P.; Punniyamurthy, T. *Chem. Commun.*, **2022**, *58*, 7090-7093.

[6] Xu, S.; Gao, Y.; Chen, R.; Wang, K.; Zhanga, Y.; Wang, J. *Chem. Comm.,* **2016**, *52*, 4478–4480.

[7] Berger, R.; Rabbat, P. M. A.; Leighton, J. L. *J. Am. Chem. Soc.* **2003**, *125*, 9596–9597.

[8] Zhao, Q.; Poisson, T.; Pannecoucke, X.; Bouillon, J-P.; Besset, T. *Org.Lett.,* **2017**, *19*, 5106–5109.
